# Comparative Transcriptomics Analysis of Testicular miRNA from Cryptorchid and Normal Horses

**DOI:** 10.3390/ani10020338

**Published:** 2020-02-21

**Authors:** Haoyuan Han, Qiuming Chen, Yuan Gao, Jun Li, Wantao Li, Ruihua Dang, Chuzhao Lei

**Affiliations:** 1 College of Animal Science and Technology, Henan University of Animal Husbandry and Economy, Zhengzhou, Henan 450046, China; 2 College of Animal Science and Technology, Northwest A&F University, Yangling 712100, Shaanxi, China; 3 Henan Genetic Protection Engineering Research Center for Livestock and Poultry, Zhengzhou 450046, Henan, China

**Keywords:** Cryptorchidism, horse, miRNA, testes, transcriptome

## Abstract

**Simple Summary:**

The testis is an important organ for mammals, and testicular microRNA expression is associated with male fertility to a certain extent. Cryptorchidism is the failure of one or both testes to descend into the scrotal sac. It is a common congenital malformation in horses. The major clinical consequence of this abnormality is impaired fertility. The expression of testicular microRNAs is influenced by many factors, including high temperature and disease, in cryptorchid horses. Here, we investigated the microRNA expression levels of normal and retained testes. Their expression patterns showed significant differences. In addition, we obtained comprehensive expression data for equine testicular microRNA, which is fundamental information for further analysis.

**Abstract:**

In the biological process of testicular spermatogenesis, the expression and interaction of many genes are regulated by microRNAs (miRNAs). However, comparisons of miRNA expression between descended testes (DTs) and undescended testes (UDTs) are rarely done in horses. In this study, we selected two UDTs (CKY2b and GU4b) from Chakouyi (CKY) and Guanzhong (GU) horses and eight DTs (GU1–3, CKY1, CKY3, CKY2a, GU4a, and GU5). Three groups were compared to evaluate expression patterns of testicular miRNA in stallion testes. Group 1 compared normal CKY horses and GU horses (CKY1 and CKY3 vs. GU1–3). Group 2 (CKY2a and GU4a (DTs) vs. CKY2b and GU4b (UDTs)) and group 3 (GU1–3, CKY1, CKY3 (DTs) vs. CKY2b and GU4b (UDTs)) compared the expression levels in unilateral retained testes to normal testes. The results show that 42 miRNAs (7 upregulated and 35 downregulated) had significantly different expression levels in both comparisons. The expression levels of eca-miR-545, eca-miR-9084, eca-miR-449a, eca-miR-9024, eca-miR-9121, eca-miR-8908e, eca-miR-136, eca-miR-329b, eca-miR-370, and eca-miR-181b were further confirmed by quantitative real-time PCR assay. The target genes of differentially expressed miRNAs in three comparisons were predicted, and the functions were annotated. The putative target genes of the 42 co-differentially expressed miRNAs were annotated to 15 functional terms, including metal ion binding, GTPase activator activity, zinc ion binding, intracellular, cytoplasm, and cancer pathways, and osteoclast differentiation. Our data indicate that the differentially expressed miRNAs in undescended testis suggests a potential role in male fertility and a relationship with cryptorchidism in horses. The discovery of miRNAs in stallion testes might contribute to a new direction in the search for biomarkers of stallion fertility.

## 1. Introduction

MicroRNAs (miRNAs) are small (approximately 22 nucleotides long) noncoding single-stranded conserved RNA molecules in eukaryotic cells that target the 3-untranslated region (3′UTR) of mRNAs [1,2]. They are encoded by miRNA genes, which are found within either introns or intergenic regions [3,4]. miRNAs play an important role in regulating gene expression as post-transcriptional factors by influencing the translation and stability of mRNAs [5]. The genes that miRNAs regulate are now known to be pertinent to nearly all fundamental cellular processes, including cell-cycle regulation and cell differentiation, development, and proliferation [6,7,8], and a total loss of miRNA function represses spermatogenesis [9,10]. 

Cryptorchidism is a common congenital abnormality resulting in male infertility [11]. It has been reported that changes in spermatozoal expression of transcriptional and antiapoptotic genes may result in poor seminal parameters in cryptorchid males [12]. Undescended testes (UDTs) in mammals is strongly related to future oligospermia [13]. Understanding the molecular mechanism in male horse testes will provide new insights regarding male fertility and contribute to marker assistant selection (MAS) in livestock and diagnostic and therapeutic approaches in humans. Studies in humans have shown that the sperm mRNA profile can serve as a molecular biomarker for evaluating male fertility [14,15]. A comparison of sperm mRNA expression profiles between fertile and infertile men with normal semen parameters showed profound transcriptomic discrepancies between groups, with potential diagnostic and therapeutic possibilities [16]. Bissonnette et al. [17] found that transcripts encoding a serine/threonine testis-specific protein kinase (*TSSK6*) and a metalloproteinase noncoding RNA (*ADAM5P*) are associated with high-motility status. 

The identification of equine testis-expressed genes is important to determine their gene expression profiles and the molecular pathways they are involved in. Recent studies showed the contribution of mRNAs and miRNAs to the formation and differentiation of spermatogonial stem cells [18]. A broad spectrum of mRNAs was detected in swine ejaculated spermatozoa, which were closely correlated with nucleic acid binding, structural modification, and transcriptional regulation. All of these categories are considered to have profound effects on the male reproductive system [19]. During mouse spermatogenesis, miR-122a was implied to target a regulated testis-specific gene, transition protein 2 (Tnp2), which is involved in chromatin remodeling [20]. Research in a cryptorchid rat found that miR-135a was downregulated in undescended testes. In addition, miR-135a is conducive to spermatogonial stem cell maintenance by regulating FoxO1 activity [21]. Recent reports found that some miRNA genes showed relatively high expression levels in stallion sperm [22], suggesting that, as in humans and mice, miRNAs might regulate spermatic function, fertilization, and early embryonic development in horses. 

Research regarding miRNA and its link to fertility has been performed on a few species, including humans and mice, but data on other animals, such as horses, have not yet been extensively examined. Recently, the development of deep sequencing and analysis approaches have stimulated more widespread and in-depth research of miRNAs expressed in the genital system, with unprecedented resolution and throughput [23]. This project was initiated to update the database of cryptorchidism miRNAs and assess their possible effects on stallion fertility [24]. Two undescended testes from unilateral cryptorchid horses and eight normal testes were collected, and their miRNA expression was evaluated and compared. It is anticipated that determining the expression profiles of miRNAs in equine cryptorchid testes will improve the understanding of their biological significance and provide a genetic basis for the discovery of biomarkers to evaluate stallion fertility.

## 2. Materials and Methods

### 2.1. Horse Testis Tissue Collection 

**Figure 1:**
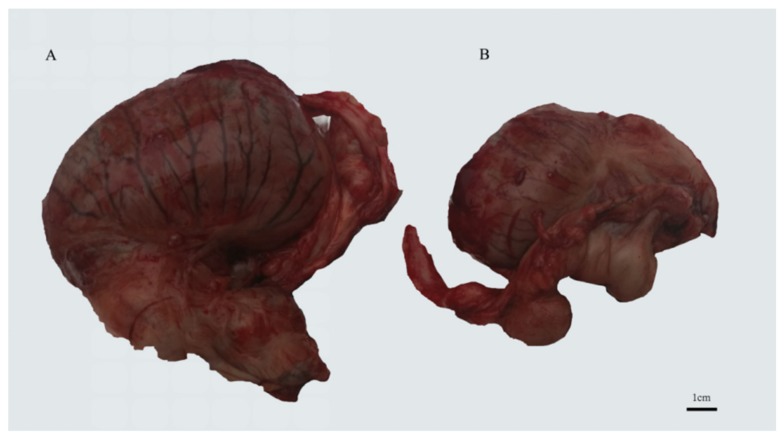
Testes of GU4 horse: (**A**) normal testis, (**B**) undescended testis.

All procedures for horse handling prior to and after castration and testis tissue collection were conducted under a protocol approved by the Animal Care and Use Committee of the Northwest A&F University. Testicular tissues from Guanzhong horses (n = 5) and Chakouyi horses (n = 3) were obtained (Table 1). One Guanzhong horse and one Chakouyi horse had unilateral cryptorchidism (Figure 1), and the 2 testes from these 2 horses, along with the remaining 6 normal testes, were collected by a veterinary surgical castration operation. The testicular tissues were immediately frozen in liquid nitrogen and stored at −80 °C prior to nucleic acid extraction in the laboratory.

### 2.2. RNA Extraction and Quality Analysis

The frozen testicular tissues were ground to fine powder and then treated by TRIzol reagent (Invitrogen, Carlsbad, CA, USA) according to the manufacturer’s protocol. RNA contamination and degradation were detected by 1% agarose gels. A NanoPhotometer® spectrophotometer (IMPLEN, Westlake Village, CA, USA) was used to monitor RNA quantity and purity. Then a Qubit® RNA Assay Kit for the Qubit® 2.0 Fluorometer (Life Technologies, Carlsbad, CA, USA) was applied to measured RNA concentration. RNA integrity was also tested via analysis using a Nano 6000 Assay Kit from the Agilent Bioanalyzer 2100 system (Agilent Technologies, Santa Clara, CA, USA) with an RIN value > 7.0. The small RNAs were used for both RNA-seq and quantitative real-time PCR (qRT-PCR) analysis. 

### 2.3. Small RNA Sequencing and Data Analysis

#### 2.3.1. Library Preparation and Small RNA Sequencing

The small RNA library was built with 3 mg of total RNA per sample. Ten libraries for the testes of 8 horses were prepared for sequencing using the NEBNext® Multiplex Small RNA Library Prep Set for Illumina® (NEB, Ipswich, MA, USA). PCR products were detected and purified on 8% polyacrylamide gel (100 V, 80 min). DNA fragments in accordance with 140–160 bp were dissolved in 8 μL of elution buffer. Finally, the Agilent Bioanalyzer 2100 system and DNA High Sensitivity Chips was used to evaluate library quality. The library preparations were sequenced on an Illumina HiSeq 2500/2000 platform, and 50 bp single-end reads were generated.

#### 2.3.2. Read Filtering and Read Mapping on the Equine Reference Genome

The 3’ adaptor sequences were trimmed by the Cutadapt v1.2.2 (http://code.google.com/p/cutadapt/) program, and reads that were shorter than 18 and longer than 35 nucleotides were removed. Reads that had a low Phred score of less than 20 for at least 80% of the bases were discarded using a FASTQ Quality Filter (FASTX-Toolkit v0.0.13.2, http://hannonlab.cshl.edu/fastx_toolkit/). Clean reads were mapped to the equine genome EquCab2.0 (GCF_000002305.2) and genes on ECAY [25] using BWA. Non-uniquely aligning reads were discarded. HTSeq-count (part of the HTSeq framework, v0.5.3p3) in “union” mode was then used to count aligned reads that overlapped with known miRBase (version 19, http://www.mirbase.org). Furthermore, reads that mapped to equine tRNA, rRNA, snoRNA, and snRNA in the Rfam RNA family database (http://rfam.sanger.ac.uk/) were also removed. Novel miRNAs were predicted using mireap (https://github.com/liqb/mireap).

#### 2.3.3. Differential Expression Analysis of miRNAs

Based on the Blast results of the miRBase database and prediction results of mireap, read counts were normalized using the DESeq2 R/Bioconductor package [26] to account for compositional bias in sequenced libraries. Three groups were compared to evaluate expression patterns of testicular miRNA in stallion testes. Group 1 compared normal CKY horses and GU horses (CKY1 and CKY3 vs. GU1–3). Expression levels in group 2 (CKY2a and GU4a (DTs) vs. CKY2b and GU4b (UDTs)) and group 3 (GU1–3, CKY1, CKY3 (DTs) vs. CKY2b and GU4b (UDTs)) were compared in unilateral retained testes to the normal testes. The miRNA differential expression (DE) was also analyzed by the DESeq2 R/Bioconductor package, and miRNAs with a threshold of |log2-fold change| > 1, padj < 0.05 were considered significantly different. Volcano and heatmap plots of testicular expressed miRNAs were produced by the DESeq2 R package. The miRBase database (http://www.mirbase.org/index.shtml) was used to predict the target genes through the human miRNAs, which are conservative to equine differentially expressed miRNAs. Function enrichment analysis (Gene Ontology (GO) and Kyoto Encyclopedia of Genes and Genomes (KEGG)) of the target genes was implemented by DAVID (https://david.ncifcrf.gov/) with a threshold of false discovery rate (FDR) < 0.05 and visualized by ggplot2 package. 

#### 2.3.4. Validation of miRNA Expression by Quantitative Real-Time PCR (qRT-PCR)

Total RNA extracted from the 10 testicular tissues used in small RNA sequencing was reverse transcribed using RR047 Reverse Transcriptase including gDNA Eraser and PrimeScript RT Enzyme (Takara, Japan). The miRNA sequences were obtained from the National Center for Biotechnology Information (NCBI) database as forward PCR primers, and the primer sequences are shown in Table S1. Typically, miRNA sequences are amplified using stem-loop RT primers, a universal reverse primer, and miRNA-specific forward primers. PCR efficiencies measured for all primer pairs were all within a range of ±10%. Each 10 μL RT-PCR reaction contained 0.8 μL of cDNA, 0.4 μL of each primer, and 5 μL of SYBR Green Real-time PCR Master Mix. PCR conditions consisted of 1 cycle at 94 °C for 30 s followed by 40 cycles at 95 °C for 5 s, 55–65 °C for 30 s, and 72 °C for 30 s, with fluorescence acquisition at 95 °C in single mode on a BioRad CFX96. All experiments were performed in triplicate. The relative deviation of Ct value was less than 10% for each sample. The comparative 2^− ΔΔCt^ method was conducted to measure the expression level of miRNA, with U6 small nuclear RNA (NR_003027) as an internal control [27]. The p-value was calculated by t-test, and *P* < 0.05 was considered significant.

## 3. Results

### 3.1. Construction of the miRNA Expression Profile

**Figure 2:**
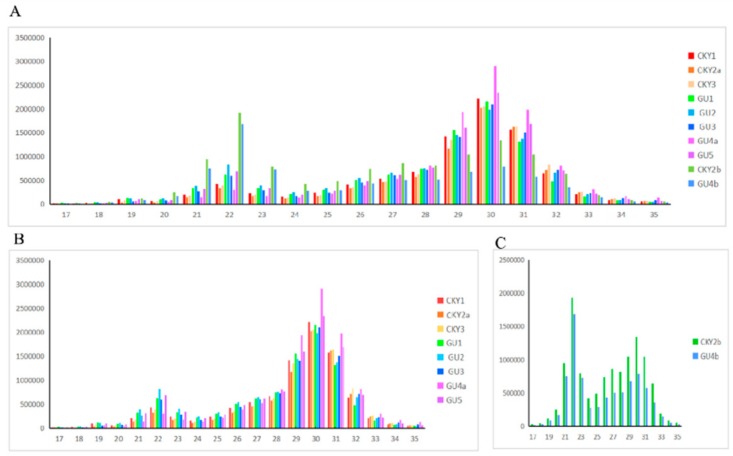
Length distribution of small RNA in equine testes: (**A**) in 10 testicular samples, (**B**) in normal testes, and (**C**) in undescended testes.

The RNA integrity number of each sample was greater than 7.0, suggesting intact RNA fragments. Deep sequencing yielded 9,818,058 clean reads in the sRNA transcriptome of the 10 equine testicular tissues on average (Table S2). It showed an obvious bimodal pattern in the size distribution of all testes reads. One was an miRNA peak (approximately 22 nt) and another was a longer piRNA-like sRNA peak (approximately 30 nt) (Figure 2). Overall, the peak in the 30 nt size class was higher and wider than that in the 22 nt size class, which indicated that piRNAs were more abundant in the normal testes (Figure 2B) and, vice versa, that piRNAs were less abundant in the undescended testis (Figure 2C). 

Our results show different expression profiles of small RNAs between DTs and UDTs. The proportion of total known miRNAs was 1,816,675 reads, which included 41,515 unique sequences on average (Table S3). The remaining 4059 reads had no matches and were considered novel miRNAs. Only the unique mapped sequences were considered as reliable miRNA molecules and used for subsequent analysis. We identified 726 miRNAs in total, including 534 commonly expressed miRNAs (Table S4). All miRNA sequences can be obtained from the Supplementary File “miRNA sequences”. A total of 646, 647, and 645 miRNAs were identified in GU1, GU2, and GU3, and 641 miRNAs expressed and showed similar expression levels in all three Guanzhong horses, with standard deviation ranging from 0.0020 to 1.0141. There were 246, 213, 205, 238, 246, 237, 225, 237, 196, and 235 novel miRNAs of CKY1, CKY2a, CKY2b, CKY3, GU1, GU2, GU3, GU4a, GU4b, and GU5. The overlapping results show that 177 miRNAs were novel and expressed in all samples. The data shown above were calculated in Excel based on Table S4 and are not displayed there.

**Figure 3:**
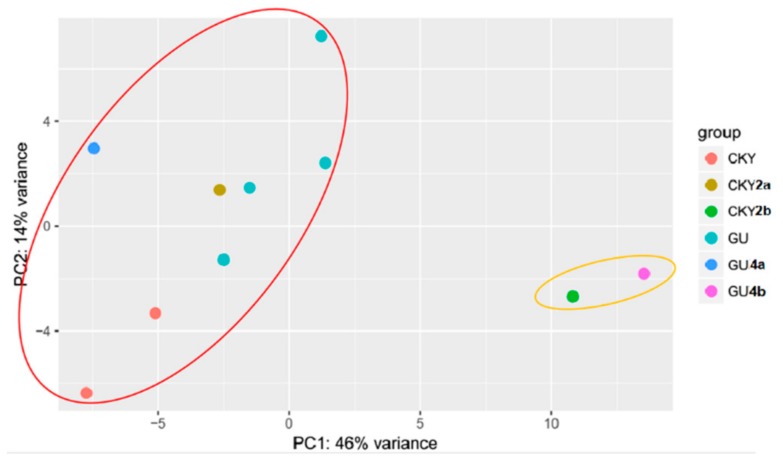
Principal component analysis (PCA) of horse testicular tissues. Colored dots represent different samples; CKY represents CKY1 and CKY3; GU represents GU1, GU2, GU3, and GU5; colored circles represent four groups clustered by PCA.

Two major classes were classified by principal component analysis (PCA). One class included eight DTs and the other included two UDTs (Figure 3). The analysis suggested different expression profiles between DTs and UDTs. 

### 3.2. Differentially Expressed miRNAs of Guanzhong and Chakouyi Horses

**Figure 4:**
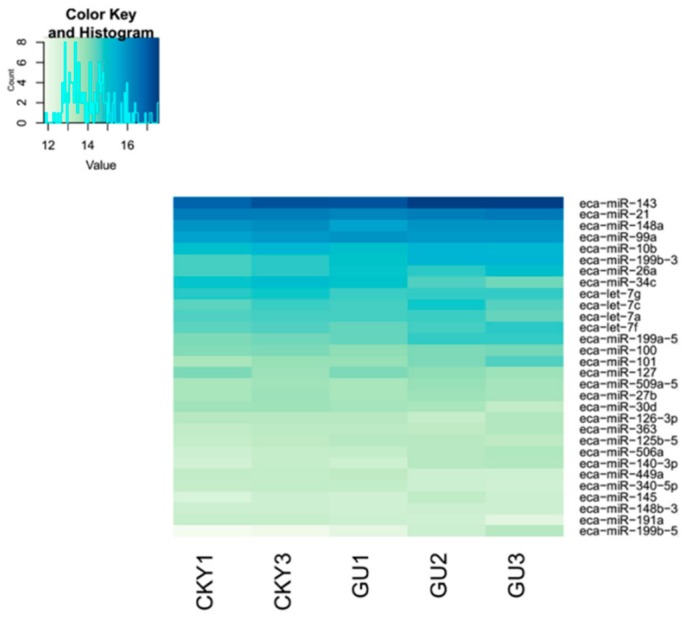
Top 30 microRNAs expressed in Chakouyi and Guanzhong horses.

**Figure 5:**
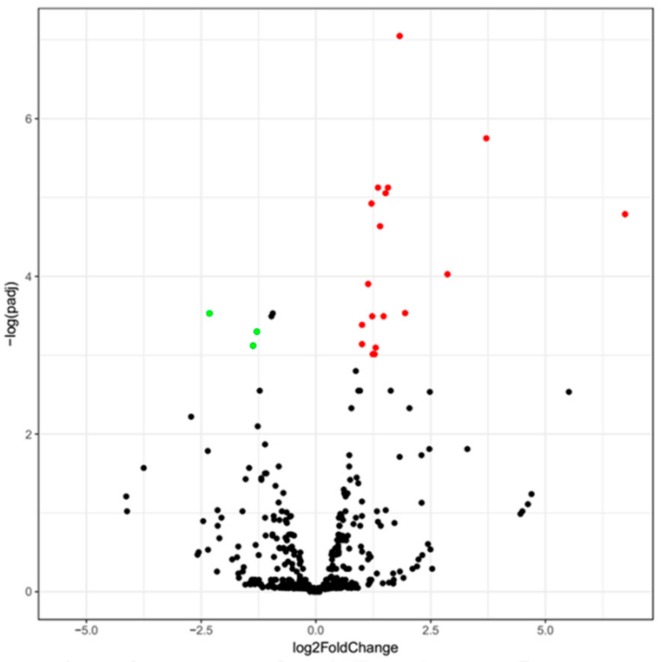
Volcano plot displaying differentially expressed microRNAs of testicular tissue between Chakouyi and Guanzhong horses. Red dots: upregulated; green dots: downregulated.

Top 30 miRNAs expressed in CKY1, CKY3, and GU1–3 are displayed in Figure 4. The transcriptome analysis identified 21 miRNAs (18 upregulated and 3 downregulated) significantly differentially expressed in Guanzhong horses compared with Chakouyi horses (Figure 5); their putative target genes were involved in axon guidance (ecb04360) and GTPase activator activity (GO:0005096). 

### 3.3. Differentially Expressed miRNAs of DTs and UDTs

The expression levels in unilateral retained testes were compared to those of normal testes. One comparison included four testes from two unilateral cryptorchid horses to compare differentially expressed miRNAs between DTs and UDTs from cryptorchid horses: CKY2a and GU4a (DTs) vs. CKY2b and GU4b (UDTs). The other comparison included five testes from normal horses and two UDTs to evaluate differential expression of testicular miRNAs between UDTs and normal horses: GU1–3, CKY1, CKY3 (DTs) vs. CKY2b and GU4b (UDTs). 

**Figure 6:**
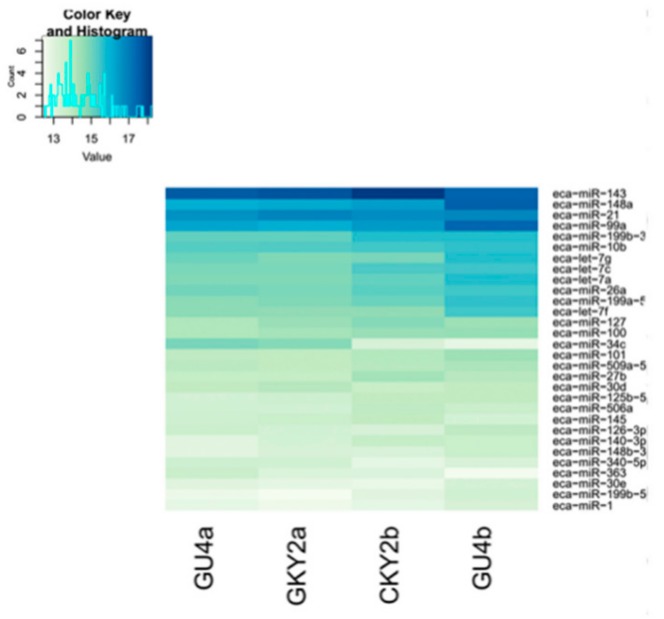
Top 30 microRNAs expressed in GU4a, GU4b, CKY2a, and CKY2b.

**Figure 7:**
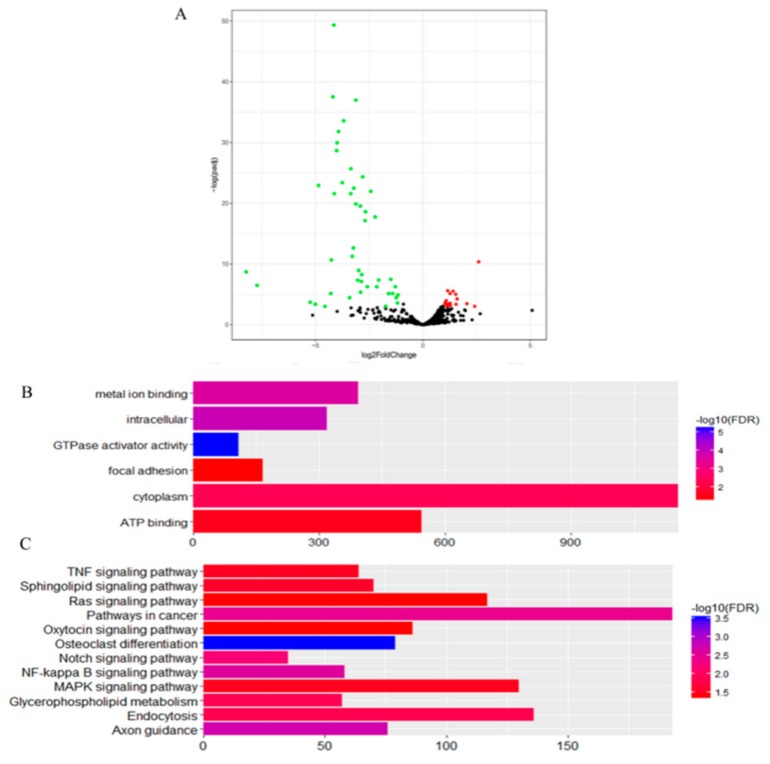
(**A**) Volcano plot displaying differentially expressed microRNAs of testicular tissue between GU4a and CKY2a compared with GU4b and CKY2b. Red dots: upregulated; green dots: downregulated. (**B**) Distribution of Gene Ontology (GO) assigned to differentially expressed microRNAs of horse testicular tissues between GU4a and CKY2a compared with GU4b and CKY2b. (**C**) Kyoto Encyclopedia of Genes and Genomes (KEGG) categories assigned to differentially expressed microRNAs of horse testicular tissues between GU4a and CKY2a compared with GU4b and CKY2b.

In the first comparison, we evaluated the miRNA expressed in CKY2 and GU4 individuals with unilateral cryptorchidism and compared the expression patterns between DTs and UDTs (Figure 6 and Figure 7). The most highly expressed miRNAs were eca-miR-143, eca-miR-148a, eca-miR-21, and eca-miR-99a (Figure 6). More downregulated miRNAs, including 46 miRNAs, were observed than upregulated mRNAs, including 16 miRNAs (Figure 7A). miRNAs primarily bind to one or more target sites on target mRNA transcripts, resulting in the degradation of mRNA and inhibition of translation, and finally regulate protein expression. Thus, the identification of target genes is an important approach to understand miRNA function. The results of the GO and KEGG annotations of the putative target mRNAs from 62 differentially expressed miRNAs are shown in Figure 7B,C. GO analysis suggested that most of the differentially expressed target genes were involved in the cytoplasm (GO:0005737), and the highest significance was found in GTPase activator activity (GO:0005096). KEGG showed 12 significant terms, with osteoclast differentiation (ecb04380) having the highest significance and pathways involving cancer (ecb05200) involving the most target genes.

**Figure 8:**
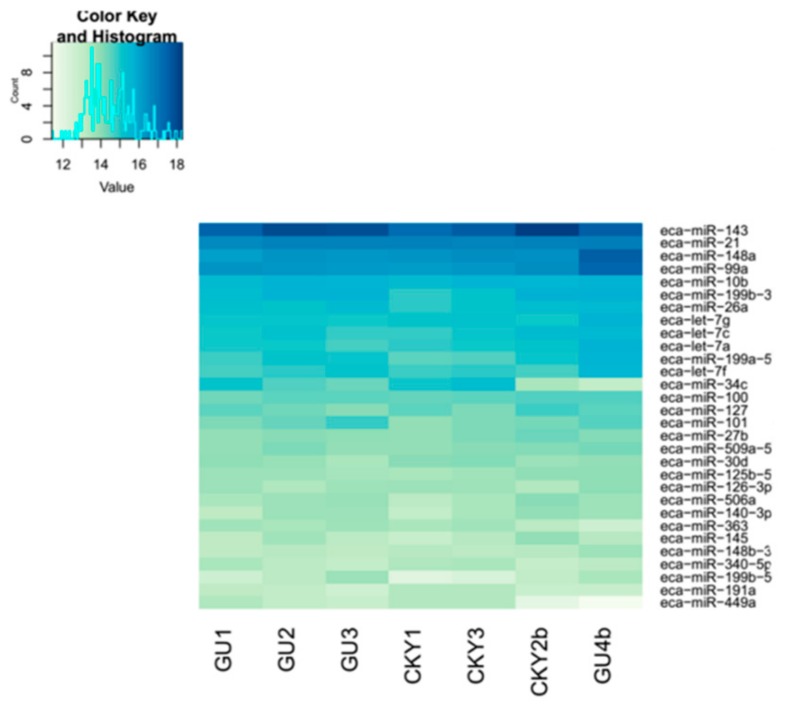
Top 30 microRNAs expressed in GU1–3, CKY1, CKY3, CKY2b, and GU4b.

**Figure 9:**
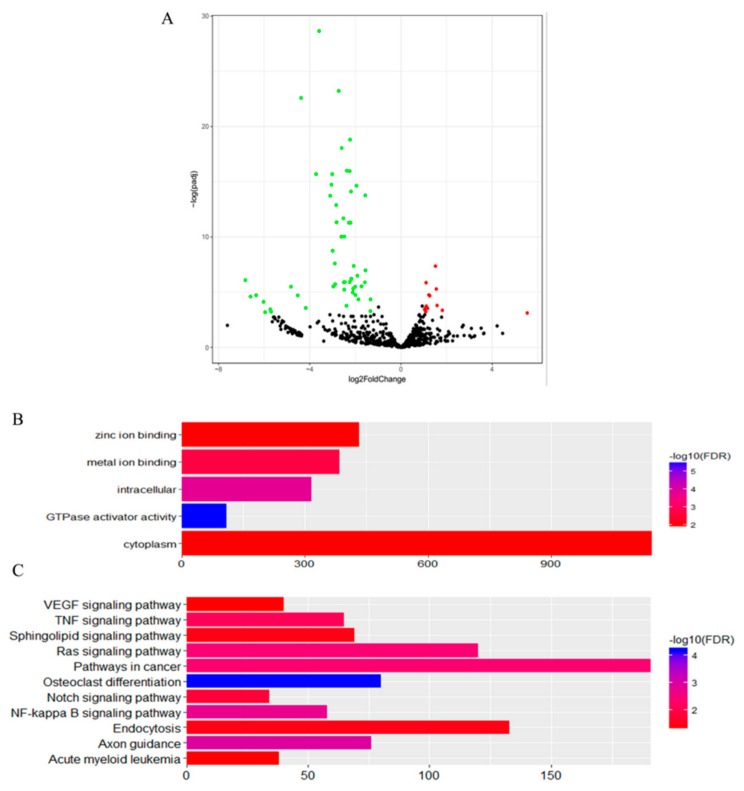
**(A**) Volcano plot displaying differentially expressed microRNAs of testicular tissue between GU1–3, CKY1, and CKY3 compared with CKY2b and GU4b. Red dots: upregulated; green dots: downregulated. (**B**) Distribution of GO assigned to differentially expressed microRNAs of horse testicular tissues between GU1–3, CKY1, and CKY3 compared with CKY2b and GU4b. (**C**) KEGG categories assigned to differentially expressed microRNAs of horse testicular tissues between GU1–3, CKY1, and CKY3 compared with CKY2b and GU4b.

In the second comparison of DTs and UDTs, the most highly expressed miRNAs were eca-miR-143, eca-miR-21, eca-miR-148a, and eca-miR-99a (Figure 8), which is consistent with the results of the first comparison. It is assumed that these miRNAs with high expression levels may be related to sperm and testis function in horses. Compared with testicular miRNAs from normal horses, 13 miRNAs were significantly upregulated and 54 downregulated in UDTs from retained testes (CKY2b and GU4b) (Figure 9A). The directly regulated transcripts were annotated in five GO terms and 11 KEGG pathways (Figure 9B,C). The annotations with the highest significance and the maximum number of involved genes included terms obtained by comparison of DTs and UDTs from CKY2 and GU4 individuals. Overall, we identified several miRNAs and mRNA transcripts that might possibly be related to testis development, cryptorchidism, and male fertility in stallions that could be regarded as candidate mRNAs in the future to investigate the mechanisms of stallion infertility.

### 3.4. Validation of Transcriptome Results by qRT-PCR

**Figure 10:**
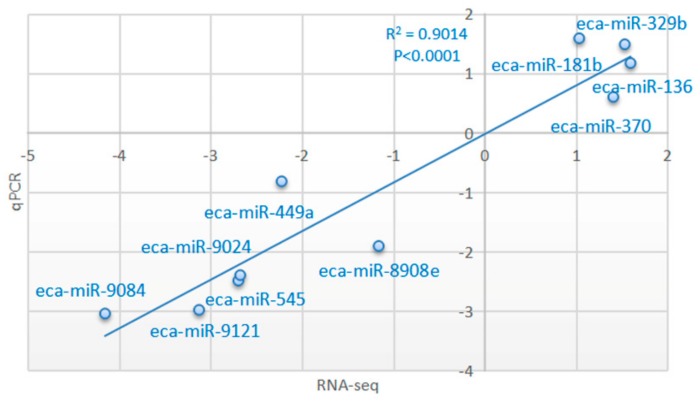
Correlations of miRNA expression levels of 10 differentially expressed miRNAs in equine testicular tissues (group 2) using RNA-Seq and qRT-PCR; the x- and y-axis show log2 (ratio of miRNA levels) measured by RNA-seq and qRT-PCR, respectively.

The expression patterns of 10 miRNAs in two comparisons (GU4a and CKY2a vs. GU4b and CKY2b; GU1–3, CKY1, CKY3 vs. CKY2b and GU4b) were analyzed using stem-loop qRT-PCR and the 2^− ΔΔCt^ method. The melt curves displayed a unique peak of each miRNA, which confirmed the specificity of the qRT-PCR amplification. The expression of six miRNAs (eca-miR-545, eca-miR-9084, eca-miR-449a, eca-miR-9024, eca-miR-9121, eca-miR-8908e) was downregulated and the expression of four miRNAs (eca-miR-136, eca-miR-329b, eca-miR-370, eca-miR-181b) was upregulated in UDTs compared to DTs, which is consistent with the expression pattern obtained by the sequencing method. To verify the accuracy of RNA sequencing results, correlation analysis was conducted, relying on the log2 value of fold change from RNA-Seq (x-axis) (group 2: GU4a and CKY2a vs. GU4b and CKY2b; group 3: GU1–3, CKY1, CKY3 vs. CKY2b and GU4b) and log2 value of 2^− ΔΔCt^ data from qRT-PCR (y-axis) method. The correlation coefficient and significance were obtained by the correlation curve (Figure 10, Figure S1), verifying the veracity and reliability of the RNA-Seq results. 

### 3.5. Expression Profiles of Commonly Differentially Expressed miRNAs in UDTs

**Figure 11:**
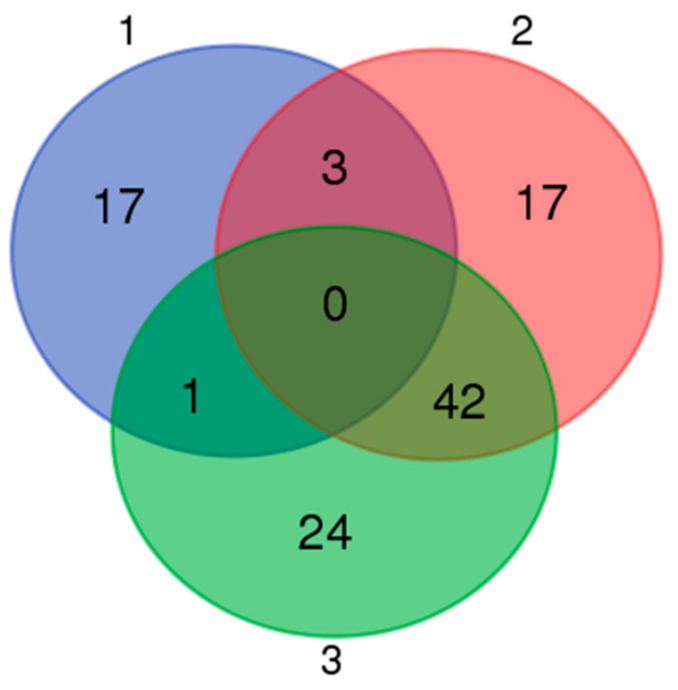
Venn map of microRNAs distributed in three groups. 1: Differentially expressed microRNAs between Guanzhong and Chakouyi horses; 2: differentially expressed microRNAs between GU4a and CKY2a compared with GU4b and CKY2b; 3: differentially expressed microRNAs between GU1–3, CKY1, and CKY3 compared with CKY2b and GU4b.

**Figure 12:**
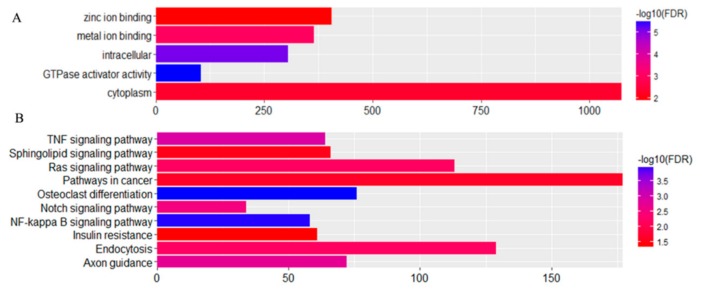
Distribution of (**A**) GO and (**B**) KEGG categories assigned to differentially expressed microRNAs of groups 2 and 3.

Based on a Venn map analysis, 42 co-differentially expressed (DE) miRNAs were discovered in both comparisons of miRNA expression in normal and retained testes (Figure 11), in which seven miRNAs were identified to be commonly upregulated by more than twofold and 35 downregulated less than 0.5-fold in UDTs compared with normal testes (Table S5). Putative miRNA target gene prediction and function enrichment showed that most of the target mRNAs were enriched in five GO terms. Regarding molecular function, the genes participated in metal ion binding, GTPase activator activity, and zinc ion binding. Regarding the cellular component, the target genes had intracellular and cytoplasm involvement (Figure 12A). These target genes were enriched in 10 pathways by KEGG analysis (Figure 12B); the cancer pathway included 177 genes, and osteoclast differentiation had the highest significance. It is tempting to speculate that the co-differentially expressed miRNAs were most likely to be related to testicular development and reproduction (Figure 12). However, the number of samples studied was too limited to draw any solid conclusions, and further investigation is needed.

## 4. Discussion

We focused on miRNAs because emerging evidence suggests that miRNAs have the potential to inhibit protein expression and degrade mRNA expression in germ cell proliferation and development in a post-transcriptional way [28,29]. It may be that as stallions sexually mature, a certain number of some miRNA transcripts are needed to regulate processes related to sexual development. A total of 10 samples were selected; one (GU5) that was expected to be undescended was identified to be clustered with descended testes based on PCA. The other testes of GU5 might have retracted into the groin because of the stress and irritation caused by castration surgery. To avoid the effects of this individual, the other nine samples were included in the analysis of differentially expressed miRNAs. Based on the miRNA expressed in these nine testes, our study identified miR-143, miR-21, miR-148a, miR-99a, and miR-10b as highly expressed in horse testes. A total of 3059 target miRNAs were identified, and they were annotated to five GO terms and five KEGG pathways (Figure 12), most of which are involved in the transcription process and cancer. Previous research found that miR-21 regulated the self-renewal of mouse spermatogonial stem cells [30]. Thus, we supposed that the other three highly expressed miRNAs might also have a potential role in testicular function. 

Cryptorchidism results in spermatogonial stem cell disturbance [13] in clinical and animal models. It is speculated that cryptorchidism is a partial cause of stallion infertility. The differential expression of miRNAs regulates the process of spermatogenesis [31,32]. Here, we compared the expression levels of miRNAs and their target mRNAs between normal and cryptorchid horses and discovered that 42 miRNAs were co-differentially expressed in two comparisons of UDTs and DTs. These commonly expressed miRNAs were upregulated or downregulated in UDTs, which may regulate the basic molecular and cellular functions in testicular tissue or putative miRNAs that regulate gene expression and protein stabilization in spermatogenesis. 

Among the co-differentially expressed miRNAs, miR-34b/c and miR-449a/b have been studied previously. miR-34b/c and miR-449 are thought to be regulators of *NOTCH1* gene expression, which is involved in the survival and proliferation of germ cells in testes [33]. Our results show that miR-34b/c and miR-449a/b were significantly downregulated in UDTs compared with DTs. miR-34c was found to be responsible for regulating genes required for the first cleavage division [34], thus, the lower expression levels of miR-34b/c in UDTs might lead to failure of spermatogenesis. BCL2 is predicted by miR-449 and mediates spermatogonial apoptosis. High levels of the BCL2 protein in male germinal cells result in highly abnormal adult spermatogenesis accompanied by sterility [35]. It is tempting to speculate that the lower number of miR-449 transcripts in the undescended testes of subfertile stallions might be related to the role of this miRNA in downregulating gene expression. 

A single miRNA can directly suppress many target genes, and certain mRNAs can have binding sites for multiple miRNAs within a 3’UTR [36]. Among the predicted target genes, *NR6A1* is expressed predominantly in developing germ cells and has functions in transcriptional regulation during meiosis and the early haploid phase of spermatogenesis [37,38]. We found that among the co-differentially expressed miRNAs, three miRNAs (miR-149, miR-8957, and miR-9049) targeting the *NR6A1* gene were downregulated. In addition, the *NR6A1* gene was annotated with a zinc ion binding function, suggesting a molecular function that might be related to male fertility in horses. *Sox5* and *Sox6* genes are highly expressed in adult mouse testes and may play a similar role in regulating gene expression in the process of spermatogenesis in adult mice [39]. However, they were not found to be related to cryptorchidism in our study. Osteoclast differentiation had the highest significance between DTs and UDTs based on KEGG analysis in our study. It was found that testosterone takes part in the process of testicular descent [40]. Testosterone and the androgen receptor (AR) are related to cryptorchidism [41]. Studies of orchidectomy-induced bone loss showed that it is associated with androgen and results in osteopenia [42]. Testosterone affects bone marrow stromal cells and osteoblasts through AR and inhibits the release of osteoclast differentiation promoting cytokines [42]. We speculate that the fluctuating testosterone of cryptorchid horses influences their bone loss, which is associated with osteoclast differentiation, and the osteoclast differentiation is regulated by the NF-kappa B signaling pathway [43], which is also a significant pathway in our study.

A novel set of small RNAs extracted from testes, called piwi-interacting RNAs (piRNAs), are slightly larger in size than miRNAs (26–31 nt) and are abundantly produced in male germline cells [44], strongly suggesting that they might play a vital role in the regulation of spermatogenesis [45]. It is worth noting that in this study, we found that there were fewer piRNAs in undescended testes compared with normal ones; this supports their key function in spermatogenesis regulation. However, the interaction between piRNA and cryptorchidism was not tested in this study, because no equine piRNA sequence has been reported or deposited in the public database [46], and piRNA sequences are not conserved in mammals [47], so further analysis should be done.

## 5. Conclusions

We combined next-generation sequencing technology with bioinformatics analysis to successfully and effectively identify and comprehensively analyze the microRNAs of normal and undescended testes in horses. Our report contributes to the understanding of miRNA expression profiling of small RNAs in cryptorchidism. The knowledge of miRNA functions in the mammalian genome might lead to new biomarkers for male fertility in horses.

## Figures and Tables

**Table 1:** Sample information of horse testes.

Sample Name	Breed	Age	Testis
GU1	Guanzhong	2 years	Descended
GU2	Guanzhong	2 years	Descended
GU3	Guanzhong	2 years	Descended
GU4a	Guanzhong	2 years	Descended
GU4b	Guanzhong	2 years	Undescended
GU5	Guanzhong	2 years	Descended
CKY1	Chakouyi	5 years	Descended
CKY2a	Chakouyi	3 years	Descended
CKY2b	Chakouyi	3 years	Undescended
CKY3	Chakouyi	4 years	Descended

## Data Availability

The full set of raw data from this study was deposited in the National Center for Biotechnology Information’s Sequence Read Archive (SRA) and is accessible through the SRA accession no. SAMN12229075-12229084.

## Supplementary Materials

The following are available online at https://www.mdpi.com/2076-2615/10/2/338/s1.
