# Supplementary material for: Comparative Transcriptomics Analysis of Testicular miRNA from Cryptorchid and Normal Horses
Source: Animals (Basel). 2020 Feb 21;10(2):338. doi: 10.3390/ani10020338 (PMC7070967; doi:10.3390/ani10020338)
Supplement: Supplementary file 1 [file animals-10-00338-s001.zip › supplement/supplement.docx]

Table S1 Information of primers used in qPCR

| microRNA | Genbank NO. | F | Tm |
| --- | --- | --- | --- |
| eca-miR-136 | NR_033006.1 | ACTCCATTTGTTTTGATGATGG | 55℃ |
| eca-miR-329b | NR_127956.1 | AACGAACCTGGTTAACCTCTTTT | 55℃ |
| eca-miR-545 | NR_033109.1 | TCAACAAACATTTATTGTGTGC | 55℃ |
| eca-miR-9084 | NR_128107.1 | TGTTTTCTCAAAGACTGATCCT | 55℃ |
| eca-miR-449a | NR_032979.1 | TGGCAGTGTATTGTTAGCTGGT | 55℃ |
| eca-miR-370 | NR_033015.1 | GCCTGCTGGGGTGGAACCTGGT | 60℃ |
| eca-miR-181b | NR_033049.1 | AACATTCATTGCTGTCGGTGGGT | 65℃ |
| eca-miR-9024 | NR_128038.1 | CAGGGACTCTGCCATGTTCTCCATT | 65℃ |
| eca-miR-9121 | NR_128152.1 | CCTTCACGGTTTCAGGGTCTTGTAC | 65℃ |
| eca-miR-8908e | NR_128186.1 | TACCCAGCAAGGTACTGATCGCTTA | 65℃ |

Note: F means the the forward PCR primers which was obtained from NCBI databases. Tm means the annealing temperature of qRT-PCR.

Table S2 Small RNA reads of equine testes

| Sample | Number of raw reads | Data size of raw reads | Number of reads after excluding adaptor | Data size of reads after excluding adaptor | Number of reads (N>5%) | Data size of reads (N>%5) | Number of clean reads | Data size of clean reads |
| --- | --- | --- | --- | --- | --- | --- | --- | --- |
| GU1 | 10,819,158 | 540,957,900 | 10,068,984 | 281,561,909 | 444 | 10,387 | 9,728,506 | 272,100,672 |
| GU2 | 11,539,348 | 576,967,400 | 10,623,266 | 295,431,074 | 428 | 10,137 | 10,256,723 | 285,304,483 |
| GU3 | 10,888,765 | 544,438,250 | 10,049,865 | 285,649,205 | 309 | 7,899 | 9,700,636 | 275,772,495 |
| GU4a | 12,800,270 | 640,013,500 | 11,478,544 | 334,736,305 | 399 | 10,751 | 11,091,037 | 323,504,284 |
| GU4b | 9,073,423 | 453,671,150 | 8,562,922 | 220,533,862 | 288 | 6,388 | 8,155,165 | 209,911,939 |
| GU5 | 11,780,354 | 589,017,700 | 11,033,795 | 311,903,029 | 449 | 11,015 | 10,677,917 | 301,913,484 |
| CKY1 | 10,858,839 | 542,941,950 | 9,902,199 | 282,822,091 | 353 | 8,475 | 9,335,775 | 266,605,497 |
| GKY2a | 9,333,423 | 466,671,150 | 8,628,667 | 250,405,923 | 302 | 8,139 | 8,343,080 | 242,160,183 |
| CKY2b | 12,959,576 | 647,978,800 | 12,233,747 | 321,700,534 | 295 | 5,844 | 11,844,274 | 311,391,494 |
| CKY3 | 10,044,669 | 502,233,450 | 9,359,332 | 269,959,650 | 342 | 8,694 | 9,047,465 | 261,029,122 |
| average | 11,009,783 | 550,489,125 | 10,194,132 | 285,470,358 | 361 | 8,773 | 9,818,058 | 274,969,365 |

Note: N>5% means the ratio of N on one read > 5%

Table S3 Reads alignment of small RNA in equine testes

| SampleID | Number of Uniq reads (Alignment to genome) | Number of total reads (Alignment to genome) | Number of Uniq reads (Alignment to miRBase) | Number of total reads (Alignment to miRBase) | Number of Uniq reads (tRNA, Alignment to Rfam) | Number of total reads (tRNA, alignment to Rfam) | Number of Uniq reads (rRNA, Alignment to Rfam) | Number of total reads (rRNA, alignment to Rfam) | Number of Uniq reads (snoRNA, Alignment to Rfam) | Number of total reads (snoRNA, Alignment to Rfam) | Number of Uniq reads (snRNA, Alignment to Rfam) | Number of total reads snRNA, Alignment to Rfam) | Number of Uniq reads (novel miRNA) | Number of total reads (novel miRNA) |
| --- | --- | --- | --- | --- | --- | --- | --- | --- | --- | --- | --- | --- | --- | --- |
| CKY1 | 656,515 | 6,457,057 | 39,341 | 1,105,558 | 6,028 | 174,334 | 10,677 | 93,871 | 4,925 | 100,599 | 2,182 | 15,455 | 439 | 3,944 |
| GKY2a | 592,292 | 5,688,144 | 33,950 | 886,680 | 5,696 | 171,615 | 9,606 | 84,821 | 5,105 | 104,405 | 1,995 | 14,518 | 218 | 1,932 |
| CKY2b | 612,119 | 8,170,751 | 58,264 | 3,954,415 | 9,718 | 354,734 | 30,269 | 302,452 | 9,593 | 233,069 | 3,060 | 18,143 | 690 | 9,012 |
| CKY3 | 607,453 | 6,495,078 | 35,341 | 1,302,485 | 7,590 | 357,132 | 11,145 | 98,435 | 4,894 | 101,481 | 2,108 | 15,886 | 330 | 2,596 |
| GU1 | 660,251 | 6,867,052 | 42,094 | 1,493,795 | 6,777 | 224,668 | 11,480 | 101,807 | 5,503 | 118,099 | 2,439 | 17,873 | 497 | 4,714 |
| GU2 | 654,806 | 7,209,196 | 45,919 | 2,083,244 | 8,725 | 373,436 | 12,548 | 107,470 | 6,815 | 149,204 | 2,480 | 16,671 | 498 | 5,191 |
| GU3 | 660,669 | 6,531,197 | 42,031 | 1,502,823 | 8,137 | 269,400 | 21,359 | 169,835 | 7,457 | 163,494 | 2,630 | 16,844 | 312 | 3,073 |
| GU4a | 744,594 | 7,842,013 | 40,231 | 1,021,940 | 10,111 | 406,599 | 11,684 | 117,079 | 6,191 | 143,985 | 2,870 | 25,852 | 304 | 2,289 |
| GU4b | 359,606 | 6,035,139 | 40,618 | 3,459,817 | 8,010 | 314,313 | 14,322 | 130,272 | 6,756 | 203,986 | 1,717 | 10,531 | 503 | 6,366 |
| GU5 | 701,361 | 7,559,737 | 44,369 | 1,852,617 | 6,266 | 254,234 | 12,229 | 105,769 | 5,914 | 142,906 | 2,482 | 17,165 | 451 | 4,182 |
| Average | 626,963 | 6,934,202 | 41,515 | 1,816,675 | 7,946 | 314,255 | 13,532 | 118,667 | 6,219 | 146,165 | 2,389 | 17,260 | 414 | 4,059 |

Note: Uniq means unique.

Table S4 The expression level of identified microRNA in 10 sanples

|  | CKY1 | CKY2b | CKY3 | GKY2a | GU1 | GU2 | GU3 | GU4a | GU4b | GU5 |
| --- | --- | --- | --- | --- | --- | --- | --- | --- | --- | --- |
| eca-miR-758 | 8.39765393 | 8.350765582 | 8.393331573 | 8.629620122 | 8.659448494 | 8.041847907 | 7.739650379 | 8.4614037 | 8.334857264 | 8.37277884 |
| eca-miR-9040 | 1.938419382 | 1.883897488 | 2.412256932 | 2.487530447 | 2.183728931 | 2.407924945 | 1.83959509 | 1.427026939 | 1.8739813 | 2.068027807 |
| eca-miR-8917 | 3.790437449 | 2.237895237 | 3.601511306 | 2.840035012 | 2.681247819 | 2.720954468 | 3.528273523 | 4.47512078 | 2.101558475 | 3.48757041 |
| eca-miR-329b | 6.838741407 | 7.731184957 | 6.985910884 | 6.880521543 | 7.223427624 | 7.110526463 | 6.863898924 | 6.801550751 | 7.576798509 | 6.973552216 |
| eca-miR-196a | 4.298685924 | 1.643008031 | 2.202360014 | 1.387595754 | 1.793589772 | 1.71694706 | 1.17533539 | 5.747162903 | 1.326522621 | 1.089497992 |
| eca-miR-502-3p | 8.472189125 | 8.71463977 | 8.829393358 | 8.690308679 | 8.589056933 | 8.607483225 | 8.297292652 | 8.71254676 | 8.791790478 | 8.700698485 |
| eca-miR-20b | 12.08184581 | 11.66318488 | 12.03229322 | 12.01280859 | 12.04726253 | 12.10043806 | 12.70129295 | 12.27563596 | 10.9059404 | 12.13643261 |
| eca-miR-450b-5p | 10.60720023 | 11.89868063 | 11.31638826 | 11.6173017 | 11.53242361 | 12.01504901 | 12.07838853 | 11.70873963 | 11.90062831 | 11.70429292 |
| eca-miR-532-5p | 11.65920669 | 11.26411469 | 11.71980157 | 11.28221305 | 11.34046849 | 11.35000302 | 11.32591795 | 11.58227163 | 11.54093464 | 11.50118431 |
| eca-miR-125b-5p | 13.58964045 | 13.76237854 | 13.70532802 | 13.64043183 | 13.78615351 | 13.72128474 | 13.58976116 | 13.56224897 | 13.72415289 | 13.67102335 |
| eca-miR-8995 | 2.710804005 | 3.779085746 | 1.952889762 | 3.079675589 | 4.08250145 | 2.660614873 | 2.580101365 | 3.59796315 | 3.479622789 | 3.382958613 |
| eca-miR-708 | 8.202882944 | 7.624151521 | 7.991404109 | 7.797359576 | 7.237791103 | 7.333535511 | 7.74555786 | 7.313786366 | 6.728623758 | 7.518325227 |
| eca-miR-495 | 8.418989134 | 7.916089094 | 7.382952984 | 7.747842914 | 8.262768045 | 7.748727175 | 7.357028011 | 7.76990138 | 8.312945606 | 7.721127978 |
| eca-miR-29b | 8.489501225 | 7.489600686 | 8.606459786 | 8.054476323 | 7.995993815 | 7.783326835 | 7.999104471 | 8.276945574 | 8.082358092 | 7.921878096 |
| eca-miR-500 | 6.340219678 | 6.025879882 | 6.542720926 | 6.560618358 | 6.164958859 | 6.21394566 | 6.255123799 | 6.583415127 | 6.404373623 | 6.870200034 |
| eca-miR-1291b | 3.84564608 | 3.501885984 | 4.245751367 | 4.903488127 | 3.844775505 | 4.836795652 | 5.487365893 | 3.83897326 | 2.573912341 | 3.797182565 |
| eca-miR-139-3p | 0.243872161 | 0.783788924 | 0.25821663 | 0.269087756 | 0.846248868 | 0.931471326 | 0.22640925 | 0.27966765 | 0.449585276 | 0.854442769 |
| eca-miR-223 | 2.428592178 | 4.514743074 | 3.304370625 | 4.458517626 | 3.954092969 | 4.994207674 | 3.941678267 | 3.23758933 | 4.435951243 | 4.598076691 |
| eca-miR-92b | 6.518095517 | 5.622150679 | 6.839448291 | 6.723709932 | 6.879926829 | 6.209092038 | 5.812322917 | 6.353396297 | 5.143050807 | 6.547985979 |
| eca-miR-8936 | 0.926853358 | 1.34233123 | 2.327336811 | 2.814916346 | 0.842808152 | 1.839551131 | 2.312774329 | 2.101679204 | 0.72914148 | 1.783118056 |
| eca-miR-545 | 7.819814648 | 6.106150032 | 8.185200416 | 7.952421897 | 7.461001085 | 7.428451751 | 7.667428544 | 8.31665727 | 5.929508429 | 7.903946567 |
| eca-miR-190a | 5.903486106 | 5.752836576 | 5.887806499 | 6.12419271 | 6.134252761 | 6.230282959 | 6.910860295 | 6.371855132 | 5.731783334 | 6.379783912 |
| eca-miR-149 | 6.225325071 | 3.294807392 | 6.132090552 | 5.065975287 | 5.282928628 | 6.186713832 | 6.594190062 | 6.667181533 | 3.39637412 | 5.922522591 |
| eca-miR-379 | 12.11257672 | 12.81123746 | 12.04068725 | 12.30276756 | 12.19307253 | 12.64711088 | 12.96888564 | 12.22821561 | 12.84152549 | 12.27802595 |
| eca-miR-590-5p | 2.952846668 | 3.002148629 | 3.067419696 | 2.5847477 | 2.219101977 | 2.890382495 | 3.385522064 | 3.5011034 | 2.88271472 | 3.293490053 |
| eca-miR-9005 | 0.825763479 | 0 | 0 | 0.525770245 | 0 | 0 | 0 | 0 | 0 | 0.960290689 |
| eca-miR-9003 | 4.905471489 | 4.640721251 | 4.292374089 | 6.898978937 | 5.69416903 | 5.154659846 | 5.371564162 | 6.183688842 | 3.719156312 | 4.904829428 |
| eca-miR-532-3p | 5.096136855 | 5.603839919 | 5.338161607 | 5.141748744 | 5.011537052 | 5.614821922 | 5.215094511 | 5.460609155 | 5.562839459 | 5.76620124 |
| eca-miR-423-5p | 10.1115819 | 10.19173397 | 10.22392301 | 10.17376821 | 10.14626982 | 10.07650418 | 9.586762668 | 10.0564373 | 10.19176095 | 10.12915655 |
| eca-miR-197 | 4.819388357 | 5.569205047 | 4.621315422 | 5.012818016 | 5.446663155 | 4.733038069 | 5.452348088 | 4.415893751 | 5.214043705 | 5.794383458 |
| eca-miR-340-3p | 7.893399978 | 6.754401304 | 7.89358112 | 7.522158378 | 7.745653427 | 7.292273118 | 6.953840662 | 8.138242001 | 7.518352568 | 7.288169498 |
| eca-miR-3958 | 9.265662271 | 9.34815396 | 8.977404535 | 9.200584647 | 9.000195784 | 8.970654723 | 8.897032872 | 8.917832307 | 9.147609924 | 8.998308067 |
| eca-miR-191b | 4.269883902 | 3.938392528 | 4.478334236 | 3.736522208 | 4.374766299 | 3.611326073 | 2.766511662 | 4.340077716 | 3.716236452 | 3.720441721 |
| eca-miR-487a | 8.604373273 | 7.299887789 | 8.1284091 | 8.093903713 | 8.052007416 | 7.686270385 | 7.689866206 | 8.56309011 | 7.473190932 | 7.686627748 |
| eca-miR-1298 | 4.670239573 | 7.422360752 | 5.472926876 | 6.612124204 | 5.325968235 | 6.819287935 | 7.385068989 | 6.380798663 | 8.543063568 | 7.098153926 |
| eca-miR-504 | 5.71779002 | 6.317202817 | 6.145327785 | 6.240493825 | 5.897805035 | 5.549596692 | 4.803500531 | 5.500642997 | 5.794333036 | 5.882879296 |
| eca-miR-30c | 12.27010744 | 12.27976764 | 12.3284195 | 12.44531671 | 12.33956611 | 12.28307067 | 12.25984552 | 12.42088063 | 12.33149264 | 12.47377116 |
| eca-miR-8971 | 6.611734418 | 4.81968864 | 6.076470066 | 6.735130954 | 5.554748026 | 5.283465863 | 6.125963563 | 7.130438951 | 3.966662389 | 5.765713146 |
| eca-miR-8998 | 6.278175858 | 4.518001536 | 6.486164274 | 5.80026486 | 4.891553969 | 5.33325946 | 6.186387659 | 6.718209441 | 4.164985537 | 5.649630474 |
| eca-miR-671-3p | 3.416702659 | 3.343022041 | 3.546274262 | 3.113750396 | 3.45735358 | 3.003779845 | 2.441930551 | 3.494569574 | 3.166110085 | 3.407795427 |
| eca-miR-8908l | 7.278390934 | 5.341243631 | 6.253207945 | 5.851878824 | 5.947392861 | 6.136602174 | 5.482415438 | 6.060661041 | 4.705422568 | 5.752397686 |
| eca-miR-410 | 7.232102159 | 7.093232636 | 6.745885328 | 7.268679732 | 7.057529394 | 6.96143408 | 7.08892828 | 6.716849259 | 7.202779359 | 6.892527596 |
| eca-miR-424 | 10.51663395 | 11.34232647 | 10.69162871 | 11.17676468 | 10.94599415 | 11.351585 | 11.54002811 | 11.21424604 | 11.44959382 | 11.13113944 |
| eca-let-7a | 15.01235897 | 14.91846683 | 15.04404761 | 14.85649706 | 14.96653686 | 14.96986234 | 14.71249058 | 14.94561599 | 15.41717881 | 14.78609296 |
| eca-miR-1197 | 6.213308835 | 5.854196743 | 5.9316582 | 5.877957148 | 5.584498944 | 5.36776274 | 4.964123364 | 5.064031382 | 5.762854068 | 5.308282386 |
| eca-miR-8984 | 10.10894139 | 8.644628416 | 9.937670746 | 11.00559698 | 9.974371246 | 10.03933077 | 10.03362723 | 11.44961523 | 8.749514268 | 10.48820226 |
| eca-miR-675 | 3.773627925 | 4.390923356 | 2.972520951 | 4.302090613 | 2.9486722 | 4.363258164 | 3.5939328 | 3.121171256 | 4.112456298 | 3.520333862 |
| eca-miR-1468 | 5.793799268 | 5.326109347 | 4.851111936 | 5.598321907 | 5.362076663 | 4.670379201 | 4.97133562 | 4.829192301 | 5.346608705 | 5.467698213 |
| eca-miR-23b | 10.4126985 | 10.3880174 | 10.70042983 | 10.34375778 | 10.43185057 | 10.57446215 | 10.37481414 | 10.52895002 | 10.42364914 | 10.58886994 |
| eca-miR-8999 | 0 | 0 | 0 | 0 | 0 | 0 | 0.103817384 | 0 | 0 | 0.011610071 |
| eca-miR-1543 | 0.130404737 | 0 | 0.19980834 | 0 | 0 | 0 | 0.044174531 | 0 | 0 | 0 |
| eca-miR-425 | 5.066959366 | 5.06021038 | 4.809844618 | 4.849659099 | 4.91490346 | 4.896624884 | 5.183923213 | 5.246418653 | 4.808678427 | 4.833593256 |
| eca-let-7d | 9.460014776 | 9.608439153 | 9.631194799 | 9.59164818 | 9.607505916 | 9.782417059 | 9.679831039 | 9.391375596 | 9.77425957 | 9.494495607 |
| eca-miR-485-5p | 5.309465363 | 5.779490253 | 4.732464529 | 5.500032077 | 5.469762403 | 5.313766568 | 5.475551252 | 4.328292394 | 5.333196197 | 5.279606837 |
| eca-miR-34b-5p | 8.353335569 | 6.492490471 | 8.575375713 | 8.404403516 | 8.452327818 | 7.898811512 | 7.857207909 | 8.629998873 | 5.933391104 | 8.100590597 |
| eca-miR-802 | 2.418049057 | 0.66237184 | 1.058586597 | 3.46355365 | 2.560077918 | 1.860205554 | 1.658822697 | 3.799516755 | 0.712795432 | 1.766933576 |
| eca-miR-551b | 0.785005272 | 0.510045482 | 0.854564728 | 0.209813388 | 0.155383895 | 0.732782187 | 0.698572842 | 0.219685931 | 0.599822168 | 0.622938032 |
| eca-miR-485-3p | 5.395379623 | 5.629812009 | 5.305885766 | 5.666402993 | 5.597723139 | 5.040704149 | 4.91591794 | 5.314286021 | 5.507431498 | 5.555856563 |
| eca-miR-214 | 10.25408638 | 10.94790547 | 10.29421539 | 10.59794018 | 10.47451975 | 10.8763507 | 10.64546112 | 10.22760688 | 11.1787169 | 10.69283302 |
| eca-miR-8986a | 3.231178902 | 2.467388784 | 3.472462634 | 3.146856564 | 2.922737635 | 3.194406952 | 2.471335869 | 3.767205262 | 3.809522665 | 3.440219502 |
| eca-miR-181b | 8.84810076 | 9.742659326 | 9.176139358 | 9.358895353 | 9.257484303 | 9.593259237 | 9.223638131 | 9.32721341 | 9.885371144 | 9.294086973 |
| eca-miR-539 | 0.493508671 | 1.511565396 | 0.512612213 | 0.526989883 | 0.942581435 | 1.439875812 | 0.470028379 | 1.309317578 | 0.918833387 | 0.449824268 |
| eca-miR-9152 | 3.024638906 | 1.956644956 | 2.918653919 | 2.25970314 | 2.22854671 | 1.831196755 | 2.365670621 | 3.547463055 | 2.322150416 | 2.605677919 |
| eca-miR-27b | 14.00615977 | 14.18532634 | 14.17314097 | 13.91990258 | 13.91374073 | 14.01893468 | 13.92018458 | 13.89121941 | 13.8535092 | 13.94491034 |
| eca-miR-8908d | 0.891472018 | 1.716881145 | 2.014500736 | 0.936093388 | 1.904777359 | 1.595250105 | 2.048884768 | 1.715590414 | 1.186961402 | 1.337358824 |
| eca-miR-200a | 6.544718108 | 6.563668416 | 6.344093975 | 6.409951668 | 6.742734566 | 6.659411335 | 7.323407952 | 6.544656255 | 6.804686299 | 6.824371464 |
| eca-miR-3548 | 2.862765496 | 2.320556197 | 1.373975524 | 1.399967057 | 2.220591573 | 1.234549977 | 1.858417964 | 2.413667287 | 2.407873065 | 2.55076945 |
| eca-miR-142-5p | 8.263721413 | 8.598204966 | 8.30604556 | 9.316729235 | 8.368831805 | 9.169342584 | 8.694253972 | 8.949476816 | 10.0361919 | 8.735237123 |
| eca-miR-19a | 9.289438686 | 8.314867502 | 9.283040112 | 9.263797775 | 9.106389175 | 8.954745132 | 9.514579985 | 9.706999486 | 9.174891899 | 9.154217792 |
| eca-miR-326 | 0.698434667 | 1.559208481 | 0.726879064 | 0.747980806 | 1.188765975 | 0.611746307 | 0.662764352 | 1.599231775 | 2.317508407 | 1.196994865 |
| eca-miR-218 | 7.421306102 | 7.449343344 | 7.470146561 | 7.278363271 | 7.3231061 | 7.48043798 | 7.914397118 | 7.543576926 | 8.245215479 | 7.320354583 |
| eca-miR-450c | 11.20818845 | 11.96642517 | 11.70486408 | 11.99488193 | 12.21575 | 12.33604361 | 12.13907702 | 12.44838907 | 12.62975382 | 11.95179415 |
| eca-miR-99b | 11.93606755 | 11.92903067 | 11.96331131 | 12.08731555 | 11.6772875 | 12.04670582 | 11.95668664 | 11.93553685 | 11.60518797 | 11.99152071 |
| eca-miR-514b | 0 | 0.544959897 | 0 | 0 | 0 | 0.212261658 | 0.687856234 | 0 | 0.491244268 | 0 |
| eca-miR-9052 | 3.245573177 | 1.963774116 | 2.522290446 | 3.696320173 | 3.582316174 | 3.364684417 | 2.959791194 | 3.981765207 | 2.095229699 | 4.156089818 |
| eca-miR-338-3p | 3.189267313 | 3.643803309 | 3.154142331 | 3.953059188 | 3.366804246 | 3.028042622 | 3.269649525 | 3.475131353 | 3.845622148 | 2.45924934 |
| eca-miR-345-5p | 6.843809862 | 6.563276538 | 7.192130871 | 7.4083642 | 6.759738003 | 6.785522782 | 6.913718121 | 7.432056927 | 6.355457045 | 7.232647522 |
| eca-miR-16 | 11.73670005 | 11.06697834 | 11.81205801 | 11.65804168 | 11.6411332 | 11.57839365 | 11.7467917 | 11.6456064 | 10.39843674 | 11.89461358 |
| eca-miR-34c | 15.09076679 | 13.23669941 | 15.2894422 | 14.86056103 | 15.07225182 | 14.58237634 | 14.28099781 | 15.10188846 | 12.72189137 | 14.91558445 |
| eca-miR-146b-5p | 10.69981469 | 9.455447257 | 10.49001492 | 10.59604569 | 10.0206279 | 11.33840103 | 9.873113461 | 10.24133357 | 10.09079381 | 10.01730553 |
| eca-miR-22 | 11.51108574 | 10.93580745 | 11.45770227 | 10.92298833 | 11.29393579 | 10.70500958 | 10.79742585 | 11.4169862 | 10.62203941 | 10.78715194 |
| eca-miR-671-5p | 4.531623144 | 3.604381807 | 4.51347112 | 3.841816631 | 4.187193027 | 4.251401712 | 3.759557852 | 4.437586208 | 3.985692502 | 4.333161163 |
| eca-miR-133a | 7.087285764 | 8.268867047 | 7.582509638 | 8.24456482 | 7.815599993 | 8.136749377 | 8.460461638 | 7.803935685 | 7.448255551 | 8.344556721 |
| eca-miR-8939 | 0.971611008 | 0.130154465 | 1.810061786 | 0.303894203 | 0.214966833 | 1.200556719 | 0.871839606 | 1.166504046 | 0.501116741 | 0.216984996 |
| eca-miR-376a | 6.911141395 | 7.525311572 | 6.398988238 | 7.106356381 | 6.831908552 | 7.063075815 | 7.426310115 | 7.03314098 | 7.378541842 | 7.151370756 |
| eca-miR-1 | 12.40195723 | 12.66331767 | 12.48507611 | 12.61410773 | 12.41750372 | 12.88079996 | 13.00102984 | 12.87065877 | 12.98819099 | 12.65293586 |
| eca-miR-130b | 0 | 0.488468781 | 0 | 0 | 0 | 0 | 0.331453602 | 0 | 0.946539887 | 0 |
| eca-miR-135b | 8.747994897 | 8.962265223 | 8.870983619 | 8.87504284 | 8.986716645 | 9.175330574 | 9.473723847 | 9.324589586 | 9.1163349 | 8.980573357 |
| eca-miR-9079 | 8.270780564 | 5.761427024 | 8.029537765 | 7.938371362 | 7.809307892 | 7.458946768 | 7.789323554 | 8.580134786 | 5.871833522 | 7.853684463 |
| eca-miR-409-3p | 10.54732139 | 11.07022049 | 10.22960014 | 10.90582006 | 10.44212134 | 10.56159026 | 10.37132998 | 10.44409857 | 10.90417942 | 10.37286329 |
| eca-miR-93 | 11.5747125 | 10.9079372 | 11.48101014 | 11.3423524 | 11.34466279 | 11.3181583 | 11.46026353 | 11.53220221 | 10.8533721 | 11.23221399 |
| eca-miR-8921 | 4.191994941 | 3.604604926 | 4.063494668 | 4.445240243 | 4.679024598 | 5.236342418 | 5.911625214 | 5.177827607 | 3.094011009 | 5.087161949 |
| eca-miR-328 | 7.478695768 | 7.276374821 | 7.569793515 | 7.466523571 | 7.27973295 | 6.865373502 | 6.719550604 | 7.294484762 | 7.080397426 | 7.405095762 |
| eca-miR-192 | 7.304450337 | 6.985553496 | 6.645418767 | 6.922712235 | 6.88955585 | 6.418319919 | 5.988222039 | 6.837915311 | 6.401466712 | 6.749530004 |
| eca-miR-8908i | 11.14059492 | 9.476661253 | 10.72427345 | 10.3821259 | 8.342335107 | 10.15245386 | 10.33132874 | 11.08239889 | 9.671013788 | 10.22703692 |
| eca-miR-9164 | 2.581061831 | 3.401397012 | 4.115752523 | 3.599978189 | 2.67952855 | 3.289720779 | 4.353132435 | 3.416220984 | 3.804157162 | 3.150973063 |
| eca-miR-1839 | 9.268801685 | 9.235864361 | 9.599145677 | 9.176070802 | 9.010153099 | 9.445827774 | 9.413024835 | 9.22911633 | 9.098839823 | 9.155004425 |
| eca-miR-23a | 10.31940211 | 10.02186995 | 10.50729069 | 9.786195652 | 10.3007531 | 10.12824829 | 9.970809493 | 10.10837027 | 9.836481117 | 10.40880077 |
| eca-miR-9084 | 8.5946125 | 6.007495156 | 8.491882899 | 8.905060924 | 7.31685752 | 6.982248824 | 8.013771128 | 9.195209013 | 5.738412068 | 7.867275001 |
| eca-miR-9002 | 3.228104105 | 2.072468632 | 2.888647485 | 2.810196763 | 2.886351442 | 2.518185605 | 3.056502512 | 3.333025487 | 2.043098614 | 2.355885894 |
| eca-miR-204b | 9.220632989 | 8.740110415 | 9.053313413 | 8.454086315 | 8.865677678 | 8.870382339 | 9.084674414 | 8.673516902 | 8.705786676 | 8.643852091 |
| eca-miR-187 | 6.581683638 | 5.850693726 | 6.582871815 | 5.796546517 | 6.285475485 | 5.823457885 | 5.898230292 | 6.174852328 | 5.809322773 | 6.19130953 |
| eca-miR-9185 | 3.670972351 | 3.934708178 | 3.443507588 | 2.340667146 | 3.641008914 | 4.306521418 | 4.40515968 | 4.356479002 | 3.653717299 | 3.346480628 |
| eca-miR-488 | 4.177896453 | 4.407372278 | 4.919636689 | 4.730009205 | 4.832526162 | 4.364214988 | 4.710143104 | 5.238488646 | 4.92003336 | 5.343622027 |
| eca-miR-363 | 13.46122988 | 12.91511577 | 13.52631607 | 13.14434735 | 13.47786689 | 13.37382567 | 13.58136529 | 13.73171336 | 12.29829904 | 13.38349211 |
| eca-miR-508-5p | 3.537130204 | 4.689234541 | 3.781882977 | 3.618487531 | 3.962135265 | 4.25304543 | 4.44776507 | 3.703389467 | 4.250761772 | 4.340073806 |
| eca-miR-138 | 6.389691691 | 4.391107343 | 6.460868629 | 5.254838842 | 5.27384299 | 3.435398979 | 4.630841967 | 4.774510761 | 3.641330321 | 4.900546616 |
| eca-miR-9049 | 7.50711154 | 5.708471065 | 7.349914687 | 7.778099389 | 6.626494367 | 7.002820343 | 7.404714758 | 8.061998081 | 5.20399293 | 6.695961677 |
| eca-miR-3959 | 8.567599913 | 7.832465491 | 8.214922255 | 8.257158701 | 8.653422951 | 7.769059055 | 8.115651901 | 8.365585314 | 8.122774535 | 8.085676615 |
| eca-miR-122 | 6.072739418 | 5.898396672 | 4.302570305 | 4.199157283 | 4.502581783 | 4.515701 | 3.898982315 | 4.034475149 | 4.583406743 | 4.475855848 |
| eca-miR-34a | 6.924482287 | 6.607543346 | 6.937614859 | 6.296692379 | 6.261691113 | 6.580622909 | 6.522915626 | 6.076432811 | 6.133809964 | 6.869835208 |
| eca-miR-423-3p | 11.05233678 | 11.38569362 | 11.31937468 | 11.43613262 | 11.21427622 | 11.19629503 | 11.11199764 | 11.2431571 | 11.00757109 | 11.32198049 |
| eca-miR-872 | 7.725108441 | 7.583178361 | 7.939645942 | 7.844373856 | 8.133910858 | 8.031122806 | 7.97761889 | 8.396168024 | 7.408657024 | 8.012803478 |
| eca-miR-378 | 10.91966596 | 10.07056944 | 11.04122201 | 10.83447926 | 10.86725725 | 10.4708989 | 10.45927577 | 11.67410667 | 10.30342682 | 10.70874494 |
| eca-miR-514 | 10.65115429 | 10.98672119 | 10.85096473 | 10.79856177 | 10.99614637 | 11.24310549 | 11.6413412 | 11.1299792 | 10.86550935 | 10.69587009 |
| eca-miR-140-5p | 10.17235157 | 10.29518022 | 10.41578114 | 10.37352024 | 10.37750693 | 10.47970805 | 10.74272793 | 10.35406701 | 10.4590482 | 10.4540341 |
| eca-miR-9080 | 0 | 0 | 0 | 0 | 0.100611919 | 0 | 0.017615418 | 0 | 0 | 0 |
| eca-miR-145 | 12.99621264 | 13.69089284 | 13.34171737 | 13.67861781 | 13.16945696 | 13.59646118 | 13.24566003 | 13.58043038 | 13.08017891 | 13.87890175 |
| eca-miR-211 | 4.791729416 | 6.063839309 | 4.875560609 | 5.174347608 | 5.012716453 | 6.299977405 | 4.998904537 | 3.785133896 | 5.327473766 | 4.724521128 |
| eca-miR-9121 | 7.025531757 | 5.109016549 | 7.04854524 | 7.560202867 | 6.68990024 | 6.24414891 | 6.35645148 | 7.751403836 | 4.418236977 | 6.285249373 |
| eca-miR-1180 | 6.164959272 | 6.214252945 | 6.472797438 | 5.876736525 | 6.258472115 | 6.431378077 | 5.911257996 | 6.156466618 | 5.843265147 | 6.315071872 |
| eca-miR-219-5p | 1.477460601 | 1.562847028 | 0.905091861 | 0.921361022 | 1.732137016 | 1.547514784 | 1.97876861 | 0.937197219 | 1.707566388 | 1.30298141 |
| eca-miR-195 | 10.82225985 | 11.10368856 | 11.01994155 | 10.87854668 | 11.03139767 | 11.03701319 | 11.43684306 | 10.8021145 | 10.71329267 | 11.04533905 |
| eca-miR-9119 | 3.513931952 | 2.363057282 | 4.060197855 | 3.405706135 | 3.887242009 | 3.689300429 | 2.367651144 | 4.040438089 | 1.891419561 | 3.655735829 |
| eca-miR-103 | 12.87295152 | 12.26115011 | 13.05595236 | 12.80693402 | 12.71629786 | 12.72346321 | 12.50961302 | 12.89292041 | 12.19726906 | 12.81795412 |
| eca-miR-582-5p | 0 | 0.07091913 | 0 | 0 | 0 | 0 | 0 | 0 | 0.168280331 | 0.098449368 |
| eca-miR-146b-3p | 3.817363515 | 3.105403903 | 3.142782492 | 2.802183394 | 3.367030341 | 4.494662147 | 1.953491366 | 2.871245483 | 3.533413078 | 3.207753048 |
| eca-miR-29c | 7.680528777 | 6.901808851 | 7.273105197 | 7.057270706 | 7.360138826 | 6.941219554 | 7.55141193 | 7.292990877 | 6.953833814 | 7.504674757 |
| eca-miR-30d | 14.12580979 | 13.58549562 | 14.1197298 | 14.10035961 | 13.95219053 | 13.83532954 | 13.49329331 | 14.04760576 | 13.67892553 | 13.94579694 |
| eca-miR-342-5p | 0 | 0.277891857 | 0 | 0 | 0.107097212 | 0 | 0 | 0 | 0 | 0.421239809 |
| eca-miR-9159 | 1.480885038 | 1.665139509 | 0.913924747 | 0.929929555 | 1.300833319 | 1.415009781 | 1.57316328 | 0.945519887 | 1.584586908 | 1.743876535 |
| eca-miR-451 | 10.10277654 | 9.64388668 | 9.264869001 | 9.1865091 | 9.431820689 | 9.036654849 | 8.993697739 | 9.794492119 | 9.234962561 | 9.540720342 |
| eca-miR-98 | 8.996777472 | 8.976740204 | 8.937965134 | 8.680158364 | 8.793128248 | 8.968900473 | 9.429776918 | 9.111775622 | 9.556991775 | 8.747356999 |
| eca-miR-551a | 0 | 0.157813973 | 0.008329436 | 0.988182319 | 0.453315275 | 0 | 0.730798782 | 0.04923533 | 0.336343759 | 0 |
| eca-miR-889 | 9.397581127 | 9.127563926 | 9.075468512 | 9.172344835 | 9.223597179 | 9.087575928 | 8.84205834 | 8.875302054 | 8.944208989 | 9.091733527 |
| eca-miR-9032 | 0.370033569 | 0 | 0 | 0 | 0.196836376 | 0 | 0 | 0.54588735 | 0 | 0.382124833 |
| eca-miR-184 | 3.036685861 | 1.032148745 | 2.471022063 | 3.601503091 | 2.576857533 | 1.427465678 | 0.916419058 | 1.075679958 | 1.431021277 | 0.866708518 |
| eca-miR-9088 | 0 | 0 | 0 | 0 | 0.111648845 | 0 | 0.569751702 | 0.468624774 | 0 | 0.118628707 |
| eca-miR-136 | 9.814556596 | 9.629675355 | 9.578741026 | 9.530641859 | 9.470436917 | 9.457221334 | 9.814977833 | 9.755499332 | 11.01497547 | 9.219417159 |
| eca-miR-324-3p | 3.767623606 | 4.703629284 | 4.200795713 | 4.546188192 | 4.16920964 | 4.674432309 | 4.55652675 | 4.483798643 | 4.280826386 | 4.374991536 |
| eca-miR-128 | 11.82940477 | 11.40652554 | 11.84021506 | 11.58489699 | 11.76233245 | 11.26349201 | 11.31971018 | 11.92334487 | 11.542196 | 11.50500413 |
| eca-miR-449a | 13.40022758 | 11.5901452 | 13.28457361 | 13.0152943 | 13.34193702 | 12.94402515 | 12.80095232 | 13.35712402 | 11.07910496 | 13.09608666 |
| eca-miR-125a-5p | 12.02505387 | 12.59995982 | 12.11976837 | 12.2924222 | 12.26258845 | 12.28373522 | 12.06594858 | 12.15944117 | 12.39754804 | 12.33040861 |
| eca-miR-8930 | 0 | 0 | 0 | 0 | 0.216670515 | 0.302572874 | 0 | 0 | 0.349214715 | 0 |
| eca-miR-33a | 1.656267198 | 1.556275777 | 1.089517809 | 1.789367853 | 2.118298108 | 1.584949412 | 1.904147453 | 1.125655702 | 2.136468186 | 1.792603674 |
| eca-miR-130a | 7.419569797 | 8.091481101 | 7.631904239 | 7.927444794 | 7.792148432 | 8.421989942 | 8.573560623 | 8.067757186 | 8.870702812 | 7.965731459 |
| eca-miR-154b | 0.964500702 | 1.706662103 | 1.647194255 | 1.704529213 | 0.908347713 | 1.33681875 | 1.823956204 | 1.023974268 | 1.525029497 | 1.95263553 |
| eca-miR-8929 | 0.207161686 | 1.447745837 | 0.225328025 | 1.012849617 | 0.163523296 | 0.153203464 | 0.988581303 | 1.067479131 | 0.862601109 | 0.165206155 |
| eca-miR-338-5p | 1.507014293 | 1.643349228 | 0.933783363 | 0.95047384 | 1.636858153 | 1.279882686 | 1.891738534 | 0.966716031 | 1.676074235 | 1.331346586 |
| eca-miR-8993 | 0 | 0 | 0 | 0 | 0 | 0 | 0 | 0.131357862 | 0 | 0 |
| eca-miR-8949 | 2.944042134 | 0.639901968 | 0.916959502 | 1.753395235 | 1.3795134 | 1.517688747 | 2.181358567 | 2.486124361 | 0.672248965 | 2.151098161 |
| eca-miR-431 | 1.162596513 | 2.232877711 | 1.200841493 | 1.229122384 | 1.9813556 | 2.371650934 | 1.732346245 | 1.25613415 | 2.997415209 | 1.828121848 |
| eca-miR-216b | 2.452665324 | 1.651786821 | 1.159006806 | 1.182616193 | 1.050939404 | 2.508735749 | 2.031514949 | 1.205339576 | 2.75907961 | 1.911580599 |
| eca-miR-206 | 2.112280479 | 0.433113751 | 0.020293433 | 0.037114384 | 0 | 0 | 0 | 0.069102995 | 1.016655233 | 0 |
| eca-miR-1249 | 1.756506053 | 2.353463308 | 1.15048133 | 1.173662747 | 1.554021714 | 1.504256004 | 1.653516218 | 2.205515646 | 1.990121466 | 1.562051234 |
| eca-miR-9038 | 6.010364871 | 4.129225522 | 5.938224184 | 5.983748507 | 5.279305011 | 5.564854349 | 5.005376121 | 6.467991493 | 4.105483985 | 5.801379325 |
| eca-miR-10b | 15.51864269 | 15.36563534 | 15.68144354 | 15.47356328 | 15.40305872 | 15.64243332 | 15.60806239 | 15.44659043 | 15.4581055 | 15.52408407 |
| eca-miR-9143 | 2.960734872 | 2.506929362 | 2.662368238 | 2.310553572 | 3.180506777 | 2.20725555 | 2.045563071 | 3.387547377 | 1.756065521 | 2.919489505 |
| eca-miR-181a | 11.38193078 | 11.96060596 | 11.4753953 | 11.74432059 | 11.69086956 | 12.12004866 | 11.95389912 | 11.73693851 | 12.40480933 | 11.81649048 |
| eca-miR-224 | 0.449953035 | 0.28981745 | 1.485532018 | 0.492785304 | 0.389769522 | 0.906005579 | 1.055164642 | 0.510254686 | 2.413255131 | 1.326044934 |
| eca-miR-126-5p | 9.433493891 | 8.764010342 | 9.177455738 | 9.269787537 | 9.431269097 | 9.145304179 | 9.456408622 | 9.260680806 | 8.720445368 | 9.737430237 |
| eca-miR-9055 | 3.471693623 | 4.375023827 | 3.939453296 | 3.524482148 | 4.284551175 | 3.80275305 | 3.728888397 | 4.482559133 | 4.147441617 | 4.17173956 |
| eca-miR-15a | 7.272089283 | 6.790818413 | 7.242639591 | 7.314324142 | 7.115401097 | 7.356955532 | 7.492325956 | 7.403092894 | 6.650705062 | 7.312444646 |
| eca-miR-507a | 10.98574592 | 11.15835079 | 11.13682286 | 10.96520302 | 11.18255854 | 11.40615654 | 11.35973177 | 11.26555731 | 11.13301625 | 10.99063889 |
| eca-miR-125a-3p | 4.282785686 | 3.808759308 | 4.867956457 | 5.08110546 | 4.871569759 | 4.541115603 | 4.613329511 | 5.490158993 | 3.704891603 | 4.237504471 |
| eca-miR-95 | 7.972784406 | 8.866031582 | 8.139045325 | 8.527374631 | 7.785214516 | 8.290238536 | 7.638186942 | 8.271715234 | 8.403210472 | 8.234440885 |
| eca-miR-9012 | 1.956934749 | 0.460269984 | 1.901704064 | 1.981448205 | 1.521705535 | 0.564586318 | 1.476966052 | 2.057730042 | 0.48544207 | 0.584870537 |
| eca-miR-2483 | 0.7083018 | 0 | 0 | 0.255086199 | 0 | 0 | 0 | 0 | 0 | 0 |
| eca-miR-148b-5p | 6.162202662 | 6.035123536 | 6.190279875 | 6.001998352 | 6.105253914 | 6.083870531 | 5.990007032 | 6.077391261 | 6.03593627 | 5.868160526 |
| eca-miR-132 | 6.111068082 | 5.003547499 | 5.632081183 | 5.341346477 | 5.964585967 | 5.268643049 | 4.439502104 | 5.750485627 | 4.809323879 | 4.977436957 |
| eca-miR-8989 | 0 | 0 | 0.271587093 | 0 | 0 | 0 | 0 | 0.645413026 | 0 | 0.022638256 |
| eca-miR-628a | 4.870604067 | 5.083865922 | 5.1190198 | 4.747081294 | 5.122699617 | 5.402656366 | 5.019094205 | 4.91500706 | 5.177484581 | 4.902871958 |
| eca-miR-664 | 2.914354642 | 3.220303542 | 3.021318583 | 3.392024443 | 3.075273116 | 3.149884875 | 3.236399507 | 3.176429608 | 2.589800084 | 3.514597108 |
| eca-miR-106b | 8.771034839 | 8.840912947 | 8.874523715 | 8.967003043 | 8.923795694 | 8.99536026 | 9.269112019 | 8.89912751 | 9.01364149 | 9.040527463 |
| eca-miR-299 | 6.353186244 | 7.38923698 | 6.595744468 | 6.834604061 | 7.139439456 | 7.366335099 | 7.584915559 | 6.794332252 | 7.491096552 | 6.894872258 |
| eca-miR-148a | 16.5316658 | 16.30670046 | 16.61488659 | 16.31359447 | 16.16069721 | 16.37999402 | 16.37051246 | 16.28289437 | 17.31950622 | 16.20985575 |
| eca-miR-215 | 2.59695608 | 2.575446688 | 3.026531332 | 3.110625601 | 2.841498586 | 3.209966077 | 2.768176674 | 3.3326813 | 2.295627197 | 2.36374849 |
| eca-miR-196b | 2.127503609 | 2.898154591 | 1.445130901 | 1.48691852 | 3.836268884 | 1.202493976 | 1.313666596 | 4.721175249 | 2.50527674 | 1.876336008 |
| eca-miR-8914 | 4.747977785 | 4.277037753 | 4.571007359 | 4.741514833 | 3.539430636 | 4.512307473 | 4.716795522 | 4.47594588 | 4.511849224 | 4.175606217 |
| eca-miR-9100 | 0.074480591 | 0 | 0 | 0 | 0 | 0 | 0 | 0 | 0 | 0 |
| eca-miR-153 | 1.101016326 | 0.568060639 | 1.920420856 | 0.462511615 | 0.372867428 | 0.86106537 | 0.398155008 | 1.551943414 | 0.641204066 | 0.915914154 |
| eca-miR-105 | 3.74886732 | 2.850176781 | 3.295666487 | 3.388959309 | 3.564687845 | 2.763001937 | 2.877839288 | 4.102370228 | 1.896131999 | 4.048189565 |
| eca-miR-497 | 9.838283472 | 10.01761709 | 9.805295061 | 9.799022319 | 9.863015394 | 9.979860304 | 10.46767919 | 9.870725922 | 10.16508633 | 9.931268537 |
| eca-miR-9065 | 1.875044597 | 0.783208532 | 1.735691706 | 2.561557024 | 1.633333341 | 1.856502153 | 1.752618254 | 2.320005393 | 0.809297903 | 2.06178815 |
| eca-miR-25 | 11.48596307 | 11.68000546 | 11.4899551 | 11.25683214 | 11.35586466 | 11.46313458 | 11.31268992 | 11.37334891 | 11.47064074 | 11.19968288 |
| eca-miR-148b-3p | 13.36566091 | 13.08771764 | 13.28285892 | 13.30777587 | 13.1192454 | 13.2317261 | 13.16950499 | 13.08361207 | 13.45574587 | 13.18601542 |
| eca-miR-505 | 5.532476088 | 6.259602453 | 5.45442853 | 5.60805515 | 5.636700285 | 5.879676021 | 5.48676246 | 5.755879664 | 5.699642706 | 5.751672262 |
| eca-miR-142-3p | 8.410892763 | 8.236375483 | 8.792973407 | 9.745176658 | 8.437077482 | 9.085029312 | 8.958874593 | 9.182133926 | 9.568101754 | 8.770442226 |
| eca-miR-28-5p | 8.857256908 | 9.18535091 | 9.103081916 | 8.893535952 | 9.189705631 | 9.245950607 | 9.196918017 | 9.066792797 | 9.17560291 | 9.134399055 |
| eca-miR-9171 | 0 | 0 | 0 | 0.703741387 | 0 | 0 | 0 | 0 | 0 | 0 |
| eca-miR-26a | 15.08303412 | 15.16565025 | 15.20393517 | 15.03164572 | 15.28477769 | 15.14695678 | 15.41981599 | 15.17024694 | 15.29548199 | 15.27244054 |
| eca-miR-369-3p | 9.426194158 | 9.734578866 | 9.150874787 | 9.430106562 | 9.368412777 | 9.369725021 | 9.253857491 | 9.145489895 | 9.555781753 | 9.2846128 |
| eca-miR-8962 | 7.815129567 | 6.048135425 | 7.764226049 | 8.318543327 | 7.45140385 | 7.051149997 | 7.201025834 | 8.320659888 | 5.234713718 | 7.776229261 |
| eca-miR-1296 | 2.142098355 | 3.90525166 | 3.590500297 | 3.985102705 | 3.4710181 | 3.379761413 | 3.192632167 | 3.507877992 | 3.456216313 | 3.844063264 |
| eca-miR-9077 | 7.998221661 | 5.212442698 | 7.506519679 | 7.954150858 | 6.643183876 | 6.492683829 | 7.238456992 | 8.009918953 | 4.819766395 | 6.652196044 |
| eca-let-7f | 14.88040174 | 14.55568083 | 14.93642885 | 14.70642692 | 14.71098914 | 14.9276091 | 15.10031308 | 14.88212106 | 15.26975341 | 14.64502189 |
| eca-miR-20a | 11.83641139 | 11.8941471 | 11.91635321 | 12.09899088 | 12.02241169 | 12.2000663 | 12.58524473 | 12.26024421 | 11.48128255 | 12.19331994 |
| eca-miR-9021 | 3.770536323 | 2.935225247 | 3.987788313 | 3.50191199 | 3.412978565 | 3.935464377 | 2.527202298 | 2.863802738 | 3.209720556 | 3.680834616 |
| eca-miR-199b-3p | 15.07193485 | 15.56750781 | 15.21437744 | 15.2398132 | 15.34761127 | 15.6808422 | 15.68002758 | 15.34497423 | 15.49671557 | 15.52882057 |
| eca-miR-1912 | 0 | 0 | 0 | 0.547567307 | 0 | 0.180982789 | 0 | 0 | 0 | 0 |
| eca-miR-873 | 0 | 0.285475782 | 0 | 0 | 0.401182382 | 0 | 0 | 0 | 0.738113909 | 0 |
| eca-miR-24 | 11.55281514 | 11.64168906 | 11.78082311 | 11.34537457 | 11.68056794 | 11.61874449 | 11.61875901 | 11.62671331 | 11.78020596 | 11.50696998 |
| eca-miR-323-3p | 9.774757564 | 9.170187726 | 9.222060076 | 8.988309525 | 9.254165937 | 8.357284539 | 7.85899695 | 8.820695683 | 8.640685528 | 8.790705388 |
| eca-miR-370 | 8.591165514 | 8.910448944 | 8.173395123 | 8.369557573 | 8.976046146 | 8.375911202 | 8.616661097 | 8.417622227 | 9.304624302 | 8.578299268 |
| eca-miR-9142 | 2.896433116 | 1.803005314 | 2.313058036 | 2.151794019 | 2.573193767 | 2.491313869 | 1.902063059 | 2.650590727 | 2.013990106 | 2.27066286 |
| eca-miR-542-5p | 3.963724473 | 4.770347035 | 3.345190644 | 4.640211773 | 4.447612202 | 5.112805394 | 4.995296676 | 4.388689955 | 5.460971135 | 4.272090154 |
| eca-miR-18b | 3.707876888 | 2.864798135 | 3.342967874 | 3.670842982 | 3.272454844 | 3.10360939 | 4.054995707 | 3.52129481 | 2.592664091 | 3.205997685 |
| eca-miR-9176 | 8.419752018 | 6.022959757 | 8.998754029 | 8.609343812 | 7.822386327 | 8.360116948 | 8.982678508 | 8.234507945 | 5.846612312 | 8.050165151 |
| eca-miR-499-5p | 7.098512688 | 5.719322482 | 7.006129923 | 6.935566476 | 7.243520567 | 6.732604188 | 6.433741577 | 7.144622096 | 6.068934566 | 7.11547085 |
| eca-miR-9182 | 0 | 0 | 0 | 0 | 0 | 0.906740091 | 0.152979861 | 0.436544007 | 0 | 0 |
| eca-miR-8922 | 0 | 0.003883427 | 0 | 0.717268003 | 0 | 0 | 0.030037284 | 0 | 0 | 0 |
| eca-miR-502-5p | 3.859472724 | 4.019961735 | 4.683772473 | 4.530444876 | 4.541353555 | 4.399309796 | 4.627809784 | 4.629375007 | 4.172342989 | 4.219869575 |
| eca-miR-506a | 13.17956675 | 13.67239827 | 13.36237896 | 13.28008769 | 13.34990667 | 13.57492501 | 13.71059371 | 13.45163233 | 13.31803603 | 13.30853167 |
| eca-miR-7 | 10.97019089 | 10.08535422 | 10.90261744 | 11.10972031 | 10.85694945 | 10.59326339 | 10.66046497 | 11.47682326 | 10.13514157 | 10.85977882 |
| eca-miR-450b-3p | 2.67834283 | 3.45239684 | 3.15249522 | 3.389973999 | 2.768545437 | 3.534876245 | 3.875270697 | 3.472215534 | 3.582452177 | 3.299708353 |
| eca-miR-19b | 11.44128588 | 10.83193178 | 11.52657576 | 11.66465481 | 11.4299614 | 11.38993911 | 11.99114415 | 11.78466345 | 10.79074888 | 11.68462336 |
| eca-miR-509b | 5.670590863 | 6.002691726 | 5.441488777 | 6.066228079 | 5.664262345 | 6.115409396 | 6.446670508 | 6.062794888 | 5.403548858 | 5.809516081 |
| eca-miR-8908g | 9.974374352 | 9.633264889 | 10.22755892 | 10.47047758 | 9.860378872 | 9.53844226 | 9.521195244 | 9.985132622 | 8.834024621 | 10.30983713 |
| eca-miR-9149 | 6.678903625 | 3.614826043 | 4.860902441 | 5.152496667 | 6.402171032 | 5.514232312 | 5.183039068 | 6.454928731 | 4.056187416 | 6.365735127 |
| eca-miR-493b | 5.374827712 | 5.637019919 | 4.718744003 | 4.487196941 | 4.858175784 | 5.021682022 | 4.173961256 | 5.29510859 | 5.538519802 | 4.839789132 |
| eca-miR-1842 | 1.696519635 | 1.438287789 | 1.776587777 | 1.120214123 | 2.074799451 | 0.985557794 | 1.035015051 | 1.893613606 | 2.045828511 | 2.372104656 |
| eca-miR-126-3p | 13.83910391 | 13.14137714 | 13.7495733 | 13.466335 | 13.72858092 | 13.3897141 | 13.71179472 | 13.77926775 | 13.74215544 | 13.73503205 |
| eca-miR-514a | 8.00289421 | 8.523055732 | 8.247287889 | 7.913159025 | 8.244033089 | 8.342329459 | 8.242567528 | 8.409345526 | 8.356053175 | 8.247195294 |
| eca-miR-194 | 6.289959128 | 5.981293028 | 5.957713827 | 6.102962829 | 6.085374425 | 6.022393455 | 5.744690399 | 6.269790409 | 5.196611448 | 5.953800579 |
| eca-miR-205 | 2.976659812 | 4.875373144 | 3.610653862 | 4.169856315 | 4.580728234 | 4.93691436 | 4.2367196 | 3.215421 | 3.775742595 | 5.093769629 |
| eca-miR-1388 | 9.758591975 | 10.2151616 | 9.888532449 | 9.832360144 | 9.945395484 | 10.2789414 | 10.08453032 | 9.922305015 | 10.51894047 | 9.852193873 |
| eca-miR-1271a | 6.410133186 | 6.891732509 | 6.533904119 | 6.16276686 | 6.525262225 | 6.587159422 | 6.353453611 | 6.101565618 | 6.651840406 | 6.464588823 |
| eca-miR-31 | 2.704041755 | 3.429365666 | 2.977803217 | 3.349273852 | 2.729748266 | 3.24477837 | 2.870991197 | 3.293306167 | 3.151156115 | 3.046814484 |
| eca-miR-9158 | 1.883818424 | 4.291822079 | 3.953363369 | 3.12741478 | 2.272392532 | 3.007484985 | 4.221525636 | 4.315144987 | 3.477635543 | 3.112221792 |
| eca-miR-146a | 10.80426263 | 11.58952138 | 11.17428166 | 11.77171939 | 11.18984058 | 12.01351321 | 11.91104088 | 11.60894465 | 11.92249603 | 11.61655704 |
| eca-miR-127 | 14.64726052 | 14.60629727 | 14.23351475 | 14.5631782 | 14.53273849 | 14.26710258 | 14.07628249 | 14.32052841 | 14.24998809 | 14.29151267 |
| eca-miR-454 | 4.954835236 | 5.69657099 | 5.429502508 | 5.696656906 | 5.226769212 | 5.458135575 | 5.728602187 | 5.249741449 | 5.386018048 | 5.486245795 |
| eca-miR-509a-5p | 13.92226006 | 13.83704758 | 13.99979364 | 13.8711788 | 13.82289594 | 14.06361294 | 13.81316378 | 14.06408345 | 13.90202219 | 14.34450293 |
| eca-let-7c | 14.92851313 | 15.21119409 | 15.09643255 | 14.94013971 | 14.97372302 | 15.15304312 | 14.82976325 | 14.99755295 | 15.2482175 | 14.82050671 |
| eca-miR-450a | 8.109384132 | 8.864126732 | 8.381308573 | 9.103106579 | 8.995711716 | 9.19896397 | 9.06122498 | 9.394218258 | 9.449684736 | 8.818307804 |
| eca-miR-432 | 0.472249711 | 1.35918885 | 0.490996526 | 0.505110468 | 0.921129063 | 0.877001424 | 0.449218984 | 1.286542315 | 1.355329922 | 0.429412786 |
| eca-miR-486-5p | 9.922860273 | 9.755175139 | 9.308617511 | 9.072706123 | 9.275352268 | 8.979922501 | 8.970441676 | 9.620828083 | 9.139702267 | 9.470325237 |
| eca-miR-15b | 7.212682588 | 7.214503647 | 7.567315469 | 7.216119821 | 7.412266991 | 7.226854642 | 6.499396205 | 7.34441097 | 6.494020053 | 7.248770991 |
| eca-miR-490-3p | 0.094529432 | 0.886362 | 0.114314686 | 0.129015572 | 0.046255063 | 0.034672529 | 0.069799813 | 0.152774928 | 1.040245314 | 0.626455205 |
| eca-miR-382 | 7.12882051 | 7.677593545 | 7.341312306 | 7.118357782 | 7.379547743 | 7.260072039 | 7.239201687 | 7.29239778 | 7.641676412 | 7.110319535 |
| eca-miR-361-5p | 6.187142482 | 6.816040031 | 6.715844633 | 6.178682891 | 6.497430352 | 6.742931081 | 6.812137679 | 6.182367482 | 6.716112769 | 6.766539789 |
| eca-miR-9060 | 4.160745927 | 3.065416233 | 3.538796419 | 3.03213202 | 3.827372072 | 3.575020895 | 2.884574723 | 2.876541678 | 2.932905503 | 3.629913866 |
| eca-miR-8931 | 7.953096902 | 6.07034963 | 6.694077011 | 8.735338376 | 7.515726813 | 7.94960691 | 7.54913964 | 9.462002252 | 6.344152976 | 8.659597037 |
| eca-miR-8908f | 5.838569765 | 3.392071295 | 5.391873777 | 4.420238669 | 4.551369097 | 3.758660528 | 4.839669168 | 5.157074618 | 2.72080443 | 4.739682466 |
| eca-miR-8942 | 0 | 0 | 1.078338278 | 0.036594151 | 0 | 0 | 1.045670995 | 1.438339198 | 0 | 0.925039475 |
| eca-miR-371-5p | 0 | 0 | 0 | 0 | 0 | 0 | 0.049529826 | 0 | 0 | 0 |
| eca-miR-183 | 1.366321986 | 2.458627137 | 1.397840683 | 2.103561108 | 2.425408985 | 2.595415993 | 1.862360009 | 2.161324296 | 2.568635404 | 2.221933485 |
| eca-miR-342-3p | 2.377900543 | 2.25744605 | 1.434533638 | 1.458991002 | 2.247663594 | 2.704133664 | 2.087695586 | 2.631729067 | 2.606039179 | 1.982250994 |
| eca-miR-493a | 6.833950838 | 4.564075219 | 6.744687115 | 6.982869918 | 5.720817788 | 6.091667956 | 6.578579321 | 7.467097162 | 3.77392094 | 6.127991012 |
| eca-miR-362-3p | 3.187584182 | 3.253026858 | 3.657186526 | 2.837174106 | 3.200970292 | 3.345143878 | 3.534007339 | 3.608190412 | 3.454607051 | 3.751476728 |
| eca-miR-10a | 10.01281596 | 9.318859328 | 9.236034819 | 9.154497687 | 9.090186012 | 8.873829876 | 9.348393381 | 9.335465375 | 8.898413637 | 9.525637455 |
| eca-miR-507b | 6.701331203 | 6.374369057 | 7.232571037 | 7.297000277 | 7.305797809 | 7.571770382 | 7.756887805 | 7.689442068 | 7.249065499 | 6.38970291 |
| eca-miR-483 | 4.221361389 | 7.845503848 | 4.189230012 | 6.359050554 | 5.407512216 | 6.344493875 | 6.436790881 | 4.504146935 | 6.948608623 | 6.076912803 |
| eca-miR-9054 | 6.543477616 | 3.881699993 | 6.382710795 | 6.49183235 | 5.78257317 | 5.363395263 | 5.8700974 | 7.253366464 | 3.097842136 | 5.697468581 |
| eca-miR-200b | 4.149089779 | 5.268133534 | 4.7487813 | 4.978675393 | 4.335500494 | 4.463417524 | 5.856107366 | 4.115280432 | 5.389505483 | 5.105544438 |
| eca-miR-212 | 1.638886649 | 1.663931801 | 2.30857642 | 1.782178801 | 1.435435796 | 1.561492928 | 0.947655252 | 2.298542691 | 0.818469951 | 0.915229388 |
| eca-miR-143 | 17.38536986 | 18.07356851 | 17.67083641 | 17.81114229 | 17.50498547 | 17.94048277 | 17.89204462 | 17.74056211 | 17.32662452 | 17.91776754 |
| eca-miR-151-5p | 9.009878013 | 9.063188594 | 9.142306445 | 8.983014906 | 9.292097369 | 9.159831259 | 9.511308287 | 9.041999623 | 8.881685161 | 9.244864047 |
| eca-miR-154a | 5.349436385 | 5.851128685 | 5.540798424 | 5.727727865 | 5.583576382 | 5.410273843 | 5.322512614 | 5.500295099 | 5.165455505 | 5.56068788 |
| eca-miR-345-3p | 4.690533264 | 5.638767544 | 4.532009731 | 4.794948189 | 4.754991806 | 5.301293356 | 5.991250874 | 4.723889447 | 6.021911573 | 5.192727634 |
| eca-miR-324-5p | 3.924820023 | 3.77713377 | 4.061165079 | 4.071440462 | 3.576802657 | 3.994749437 | 3.484673929 | 3.695985427 | 3.66267776 | 3.794410213 |
| eca-miR-409-5p | 10.050941 | 10.19457754 | 9.966256819 | 10.29222572 | 10.03679235 | 9.849387434 | 9.073419553 | 9.839895469 | 9.771472387 | 9.833433438 |
| eca-miR-1248 | 0.924399857 | 0.975524645 | 0.335287175 | 1.04857416 | 0.752302398 | 0.710835856 | 0.30182262 | 0.357842009 | 0.97806085 | 0.759026361 |
| eca-miR-30e | 12.65829638 | 12.53836253 | 12.71935761 | 12.84420813 | 12.85588694 | 12.88449178 | 13.2844059 | 13.08795699 | 13.03489133 | 12.6791023 |
| eca-miR-193a-5p | 3.33541827 | 3.986129739 | 3.460179262 | 4.036855219 | 3.432499618 | 3.631266824 | 2.669920061 | 2.352853682 | 4.188481381 | 4.545717079 |
| eca-miR-660 | 9.887835067 | 9.300158366 | 9.939804455 | 9.655907573 | 9.55417611 | 9.5704431 | 9.760870128 | 9.79104312 | 9.173830838 | 9.866142129 |
| eca-miR-8976 | 1.779115006 | 1.073869473 | 1.633911017 | 1.696758982 | 2.798861989 | 0.758648354 | 1.441951263 | 2.232889201 | 0.672842443 | 1.707252254 |
| eca-miR-8908n | 3.166141721 | 3.269498123 | 3.44059881 | 3.672783694 | 4.333703202 | 4.613562857 | 5.076037161 | 3.452096837 | 3.836451882 | 4.105938275 |
| eca-miR-361-3p | 8.490546606 | 8.608026817 | 8.732784472 | 8.864508219 | 8.572687592 | 8.599346676 | 8.206923504 | 8.723769171 | 9.47694205 | 8.996347398 |
| eca-miR-377 | 6.288558792 | 5.820855619 | 6.187163321 | 6.304334458 | 6.06193959 | 5.633121031 | 6.165895191 | 6.307811471 | 6.522513228 | 6.110535349 |
| eca-miR-186 | 12.24393466 | 11.92933967 | 12.27239284 | 12.16043537 | 12.02347257 | 12.13567369 | 12.49175709 | 12.16459571 | 12.21159046 | 12.08101818 |
| eca-miR-590-3p | 6.784326333 | 6.897421025 | 6.977799927 | 7.253719754 | 7.040205085 | 7.052495937 | 7.549515927 | 7.044582633 | 6.762876528 | 7.411896576 |
| eca-miR-3613 | 0 | 0 | 0 | 0 | 0.246126979 | 0 | 0 | 0 | 0 | 0.115212996 |
| eca-miR-208a | 1.230851363 | 0.08520415 | 0.201450272 | 0.213783444 | 0.146014294 | 1.0496565 | 0.164875124 | 0.226226566 | 1.160178964 | 0.842039393 |
| eca-miR-8908j | 11.30843887 | 10.22550463 | 10.95975241 | 10.77048833 | 10.40275276 | 10.37432609 | 10.20384598 | 10.698209 | 9.473489914 | 10.63338882 |
| eca-miR-9179 | 0 | 0 | 0.487703568 | 0.540127844 | 0.245608159 | 0 | 0.329598837 | 0 | 0 | 0.252349736 |
| eca-miR-656 | 8.912051355 | 8.630131983 | 8.541360359 | 8.950646521 | 8.82105105 | 8.022351363 | 7.943594218 | 8.697576757 | 8.140920859 | 8.63716128 |
| eca-miR-380 | 6.399210604 | 6.771239627 | 5.690473685 | 6.244250198 | 6.62184537 | 6.462950555 | 6.354591873 | 6.059459551 | 6.665745687 | 6.270116018 |
| eca-miR-362-5p | 8.539394509 | 8.033318566 | 8.473781144 | 8.220718737 | 8.221891534 | 8.259504743 | 8.364269017 | 8.7625946 | 7.720793566 | 8.416089872 |
| eca-miR-744 | 7.099756407 | 6.983952559 | 6.998369715 | 7.042531008 | 6.820327121 | 6.817522639 | 6.578011468 | 7.038533143 | 7.31054628 | 7.058849554 |
| eca-miR-193b | 5.034071849 | 4.326326883 | 5.14509351 | 4.94228099 | 4.709668202 | 4.529116088 | 5.495041516 | 4.594453783 | 4.326582166 | 5.315910239 |
| eca-miR-494 | 11.04652278 | 11.15582846 | 10.68581932 | 11.15321375 | 10.87579037 | 10.28482073 | 9.93924625 | 10.70334197 | 10.60161349 | 10.65409648 |
| eca-miR-8979 | 6.534196636 | 5.072769366 | 7.561607841 | 7.878152633 | 6.752141724 | 6.250261787 | 6.585204652 | 8.384165175 | 4.844471797 | 6.872583954 |
| eca-miR-8950 | 4.113078286 | 2.316491321 | 2.794435207 | 4.117343827 | 3.901879367 | 2.484230134 | 4.065715134 | 4.716127384 | 1.412458598 | 2.849898139 |
| eca-miR-487b | 8.797365832 | 9.196833776 | 8.668916087 | 8.638609254 | 8.614991638 | 8.723595845 | 8.15705467 | 8.480066396 | 8.733588107 | 8.341733001 |
| eca-miR-374a | 11.17132329 | 11.13074532 | 11.36952568 | 11.33400929 | 11.42127272 | 11.28369775 | 11.79418796 | 11.5821772 | 11.44561256 | 11.65337565 |
| eca-miR-8915 | 0 | 0.454975966 | 0 | 0 | 0 | 0 | 0.06495365 | 0 | 0 | 0 |
| eca-miR-106a | 12.25914857 | 11.98708807 | 12.12705497 | 12.25617564 | 12.32821865 | 12.3529985 | 12.93818938 | 12.47642593 | 11.5189817 | 12.46308253 |
| eca-miR-340-5p | 13.49533116 | 12.72473707 | 13.49553147 | 13.4547216 | 13.43491021 | 13.13912135 | 13.01366116 | 13.59083001 | 12.96093209 | 13.56053205 |
| eca-miR-491-5p | 0 | 0.221276581 | 0 | 0.694416932 | 0 | 0.52447465 | 0.488456458 | 0.02361908 | 0.296287757 | 0 |
| eca-miR-9014 | 7.128958165 | 6.028929598 | 7.727919411 | 8.502646882 | 6.478189168 | 6.203771024 | 6.394854462 | 7.537134643 | 4.477027085 | 6.366429551 |
| eca-miR-676 | 7.738192361 | 8.196245354 | 8.118227042 | 7.875588105 | 7.882763422 | 7.894595027 | 7.979311709 | 7.858013955 | 8.173077506 | 7.676149342 |
| eca-miR-9004 | 4.798908645 | 3.364955581 | 5.04803348 | 4.372101964 | 4.654828599 | 3.987967463 | 5.061474843 | 5.714959266 | 3.907421895 | 4.888656722 |
| eca-miR-8957 | 7.214705641 | 5.216664804 | 7.822852619 | 8.032318141 | 6.798945374 | 6.537411648 | 7.183857399 | 7.863320302 | 4.717723688 | 7.976804674 |
| eca-miR-30b | 11.32169429 | 10.86452824 | 11.30105325 | 11.10108898 | 11.17619565 | 11.06720872 | 11.35351948 | 11.1025741 | 10.73786653 | 11.28766986 |
| eca-miR-1185 | 0 | 0.214749166 | 0 | 0 | 0 | 0.274887026 | 0.393772468 | 0 | 0.66880499 | 0 |
| eca-miR-9043 | 4.503816088 | 2.944344752 | 2.850173578 | 4.616628727 | 4.570884007 | 3.462783473 | 4.513795461 | 4.94182217 | 2.105935276 | 4.316861647 |
| eca-miR-9024 | 8.169265077 | 6.432053552 | 7.802146602 | 8.254225948 | 7.812859651 | 7.70873302 | 7.999502247 | 8.678981691 | 6.216102727 | 7.694071122 |
| eca-miR-433 | 5.915279522 | 6.465714352 | 5.451099776 | 5.921422439 | 6.120908629 | 6.28399568 | 5.068834673 | 5.95801034 | 6.220674961 | 5.514445521 |
| eca-miR-222 | 7.673234525 | 7.040306301 | 7.826331804 | 6.73003975 | 7.14542684 | 6.750503955 | 7.248765516 | 7.240245589 | 6.857577965 | 7.62875776 |
| eca-miR-9170 | 0.796080129 | 1.011053035 | 1.94036936 | 0.839150803 | 1.582083551 | 1.197906636 | 1.851812429 | 2.250133164 | 1.21218238 | 1.437258124 |
| eca-miR-129b-5p | 5.660844558 | 6.077002731 | 5.661636375 | 5.940680137 | 5.09182734 | 4.777790314 | 4.696119271 | 5.408042536 | 5.417032553 | 5.691934075 |
| eca-miR-429 | 2.537672927 | 2.065872985 | 2.649273219 | 2.381278758 | 2.128764141 | 1.92242159 | 2.627912687 | 2.210363678 | 2.016069348 | 2.487147637 |
| eca-miR-486-3p | 5.982065824 | 6.450864816 | 5.918495869 | 5.114769745 | 5.594516017 | 5.752565421 | 5.699391721 | 6.088722684 | 5.424212726 | 5.653199093 |
| eca-miR-1379 | 7.361305694 | 7.289821142 | 7.227494871 | 6.829351253 | 7.4565906 | 7.318177596 | 6.971783588 | 7.463120467 | 7.355021183 | 7.126165452 |
| eca-miR-508-3p | 11.95729104 | 12.72733124 | 12.50787102 | 12.41003701 | 12.78664658 | 12.94680147 | 12.95415866 | 12.72371775 | 11.98789468 | 12.62506422 |
| eca-miR-1307 | 8.006561913 | 8.067596771 | 8.018007249 | 8.204600763 | 8.105381138 | 7.969750126 | 7.770976809 | 8.21669398 | 8.511547083 | 8.043504122 |
| eca-miR-28-3p | 11.20226665 | 11.4286824 | 11.25017772 | 11.24598308 | 11.20145676 | 11.42686374 | 11.32807205 | 11.18816383 | 11.41976506 | 11.34830459 |
| eca-miR-92a | 12.57927426 | 12.93654207 | 13.04418253 | 12.99074205 | 12.96745458 | 12.94190439 | 13.18216994 | 13.13559746 | 12.24011738 | 13.03545602 |
| eca-miR-101 | 13.99560201 | 14.02110109 | 14.23371182 | 14.03085932 | 14.15257424 | 14.42517797 | 14.84185886 | 14.13798918 | 14.31842797 | 14.32002792 |
| eca-miR-8908h | 9.663025747 | 7.970839436 | 9.451332425 | 9.072980559 | 8.752388906 | 8.61197501 | 8.790684453 | 8.94005097 | 7.472537764 | 8.760750262 |
| eca-miR-221 | 10.00339689 | 8.641803932 | 9.740879148 | 8.817195837 | 9.352261019 | 8.339579179 | 8.50488596 | 8.902493607 | 8.241336031 | 8.995131818 |
| eca-miR-8908b | 10.09854425 | 8.443758055 | 9.634944587 | 9.556289348 | 9.264940074 | 8.776829819 | 9.379203131 | 9.734865778 | 8.142576324 | 9.183822865 |
| eca-miR-376c | 4.439377258 | 6.080820214 | 5.271248266 | 5.659424767 | 5.444522928 | 5.532627283 | 6.605615018 | 5.535210995 | 6.184313029 | 5.684313109 |
| eca-miR-135a | 6.086773302 | 7.13215979 | 6.566477157 | 7.007611481 | 6.575934011 | 7.118772713 | 7.948103863 | 6.766742638 | 7.251186528 | 6.932043757 |
| eca-miR-503 | 9.242432536 | 10.32777234 | 9.161950005 | 10.38867725 | 9.797057754 | 10.34275167 | 10.79389678 | 10.21251484 | 10.52724363 | 9.693679535 |
| eca-miR-129b-3p | 3.347766437 | 3.763604449 | 3.337462951 | 3.874188464 | 3.395063369 | 3.075731989 | 3.640461314 | 3.345266375 | 3.007495504 | 3.162348208 |
| eca-miR-8909 | 10.99137267 | 9.170486686 | 10.61114249 | 10.9954554 | 10.3736689 | 10.42614202 | 11.00975789 | 11.31528246 | 8.55220597 | 10.55871425 |
| eca-miR-144 | 6.264544019 | 4.944945221 | 4.661963487 | 4.400212304 | 5.712889228 | 5.01224812 | 4.968838857 | 5.917749744 | 6.074683246 | 4.65919327 |
| eca-miR-383 | 8.268773878 | 7.889931843 | 8.389432671 | 7.777536839 | 7.981572743 | 8.04455221 | 7.428333395 | 7.911168428 | 7.761587908 | 7.653637354 |
| eca-let-7g | 15.23029027 | 14.81699655 | 15.24159702 | 14.91407811 | 15.02499102 | 15.01419107 | 15.00858057 | 15.21859774 | 15.45622465 | 14.88659888 |
| eca-miR-652 | 6.810506913 | 6.687309325 | 6.782477148 | 6.33736886 | 6.639665841 | 6.699604183 | 6.580944164 | 6.5447564 | 6.16072555 | 6.595309708 |
| eca-miR-2114 | 6.347004766 | 6.846367864 | 6.471501293 | 6.284726845 | 6.64769034 | 6.88811005 | 6.828734859 | 6.617829583 | 6.504980684 | 6.502279018 |
| eca-miR-190b | 4.749928826 | 4.048322611 | 4.857490427 | 4.042888502 | 4.263484047 | 4.288012603 | 3.996344556 | 4.340438516 | 3.859263794 | 4.133166766 |
| eca-miR-96 | 1.738807752 | 2.971106547 | 2.41475442 | 3.051725066 | 2.289186037 | 3.02499146 | 3.03840336 | 2.967985292 | 3.32391891 | 2.975594555 |
| eca-miR-496 | 0.103727688 | 1.024911652 | 0.792181382 | 0.12771478 | 0.720291039 | 0.064231786 | 0.087075439 | 0.147428521 | 0.706373739 | 0.556204997 |
| eca-miR-7177b | 1.436508687 | 2.877151215 | 2.128700104 | 1.497757732 | 2.295517111 | 2.099733487 | 2.435477151 | 2.250154808 | 2.373908832 | 2.306895382 |
| eca-miR-9147 | 0 | 0 | 0 | 0.969657706 | 0 | 0 | 0 | 0.317285164 | 0 | 0 |
| eca-miR-34b-3p | 8.702966823 | 7.022501107 | 8.796126728 | 8.084472851 | 8.440974759 | 7.877794206 | 7.398569884 | 8.181625886 | 6.416861901 | 8.260956466 |
| eca-let-7e | 11.12441648 | 11.38875388 | 11.06109023 | 11.11643569 | 11.20379688 | 11.27003324 | 10.96286842 | 11.22599171 | 11.70892929 | 11.34159947 |
| eca-miR-329a | 3.908315261 | 5.119021275 | 4.036982068 | 4.131847635 | 4.62847156 | 4.411710315 | 3.744758813 | 4.222456697 | 5.207003525 | 4.645554683 |
| eca-miR-411 | 11.66086063 | 12.43726085 | 11.73648444 | 12.00334205 | 11.99205779 | 12.19300547 | 12.62339836 | 12.01603665 | 12.23180123 | 12.09660416 |
| eca-miR-767-5p | 4.114603674 | 3.526463041 | 4.404341494 | 4.580613836 | 4.712248975 | 4.117527704 | 3.113603268 | 4.973188505 | 2.61867053 | 4.838090067 |
| eca-miR-8944 | 0 | 0 | 0 | 0 | 0 | 0 | 0.143066984 | 0 | 0 | 0 |
| eca-miR-296 | 1.050481343 | 1.704623867 | 2.337941278 | 1.100938523 | 2.35023718 | 1.878139403 | 1.581996642 | 2.328946663 | 1.209214459 | 1.826461737 |
| eca-miR-568 | 1.691015614 | 0 | 0.747270011 | 0.808563122 | 0 | 0 | 0 | 0.86723163 | 0 | 0 |
| eca-miR-8908e | 10.8155177 | 8.996338007 | 10.27855404 | 9.961561841 | 10.40754201 | 9.783595247 | 9.836443986 | 10.05671138 | 8.832652679 | 9.734998085 |
| eca-miR-140-3p | 13.11600973 | 13.64806168 | 13.47781204 | 13.39198435 | 13.12763078 | 13.69146456 | 13.73589179 | 13.08386357 | 13.44277769 | 13.64327961 |
| eca-miR-592 | 7.824168191 | 6.864397606 | 7.464753099 | 7.666711827 | 6.848186222 | 7.112764371 | 6.085667773 | 6.600431598 | 6.326398729 | 7.162416056 |
| eca-miR-365 | 5.385820094 | 5.409364259 | 5.941656516 | 5.729692843 | 5.549074455 | 4.807355723 | 5.217815134 | 5.24569275 | 4.811666646 | 5.786531941 |
| eca-miR-9150 | 2.233645649 | 1.069064176 | 1.494058181 | 2.003144995 | 1.815993154 | 1.163183054 | 0.708471646 | 1.612135399 | 0.932269391 | 0.679548547 |
| eca-miR-542-3p | 10.64284142 | 11.71034871 | 10.99864941 | 11.46727665 | 11.15534972 | 12.0432541 | 12.07781563 | 11.59792055 | 12.10210834 | 11.50636227 |
| eca-miR-543 | 7.907681267 | 7.2955024 | 6.776295874 | 6.891287577 | 7.653211593 | 7.271336391 | 6.197082267 | 7.291740444 | 7.521670374 | 6.84480843 |
| eca-miR-9062 | 1.787514795 | 0.782287787 | 1.108133098 | 3.119101406 | 2.080706301 | 2.340283335 | 2.234131032 | 3.315370547 | 0.820894563 | 2.223207055 |
| eca-miR-350 | 5.160467116 | 5.455798615 | 5.356933522 | 5.228690736 | 5.882678857 | 5.259108542 | 5.586971296 | 5.484958799 | 5.187937617 | 5.999878917 |
| eca-miR-99a | 16.28884107 | 16.32378041 | 16.35917196 | 16.18250574 | 16.4224571 | 16.32255805 | 16.26946226 | 16.45675173 | 17.18485992 | 16.38031927 |
| eca-miR-301a | 3.965849003 | 2.42404501 | 2.999053878 | 3.416153519 | 3.130728642 | 3.670478717 | 3.563983214 | 3.878399798 | 3.999789343 | 3.784988173 |
| eca-miR-199a-5p | 14.59307839 | 14.87012541 | 14.71614824 | 14.8296645 | 14.74668831 | 15.07704712 | 15.07801906 | 14.80284989 | 15.37158869 | 14.90301307 |
| eca-miR-32 | 8.469530581 | 7.539985435 | 8.485532486 | 8.089347 | 8.4531944 | 8.083886747 | 8.640733556 | 8.738507758 | 8.667981885 | 8.044766049 |
| eca-miR-134 | 7.506366037 | 7.895501268 | 7.014703957 | 7.597325717 | 7.744469616 | 7.470111565 | 7.572415671 | 7.271502026 | 7.670109069 | 7.404535121 |
| eca-miR-8908c | 6.7370201 | 4.580683302 | 6.338984191 | 5.336649338 | 5.852407242 | 4.980318745 | 5.750633648 | 5.394766335 | 4.555025479 | 5.583344632 |
| eca-miR-8932 | 3.454023671 | 1.31005848 | 3.244440471 | 2.564682536 | 3.925804406 | 2.801552156 | 3.89950734 | 4.487872535 | 1.763479352 | 3.900533993 |
| eca-miR-133b | 1.216353108 | 3.250855157 | 1.255948841 | 1.285215817 | 2.407745755 | 2.868914517 | 3.219455441 | 1.31316145 | 1.352847839 | 2.042651086 |
| eca-miR-1264 | 0 | 0.810428037 | 0 | 0.009880353 | 0 | 0 | 0 | 0.02958289 | 1.174260898 | 0 |
| eca-miR-191a | 13.44939624 | 12.58601174 | 13.37670891 | 12.98389436 | 13.11133713 | 12.98018935 | 12.55690437 | 13.11582042 | 12.604329 | 13.00855129 |
| eca-miR-8908m | 4.451590708 | 2.808419441 | 4.013441537 | 3.293958153 | 3.609728411 | 3.324204005 | 3.055676747 | 3.52613801 | 2.493724408 | 3.016548944 |
| eca-miR-8941 | 8.294673351 | 5.745675074 | 6.683777828 | 8.293285943 | 8.223424693 | 7.373885746 | 8.385598574 | 9.555113585 | 6.117194185 | 7.95738059 |
| eca-miR-337-3p | 0 | 0.83231141 | 0 | 0 | 0 | 0 | 0.434485066 | 0 | 0.511791625 | 0 |
| eca-miR-874 | 6.926502338 | 7.856852052 | 7.251811489 | 7.434212505 | 7.53705583 | 7.997274601 | 8.019784204 | 7.608356006 | 7.524371705 | 7.688229051 |
| eca-miR-217 | 1.034663323 | 0.591020706 | 0.337423506 | 2.110425548 | 0.833198352 | 0.972032334 | 0.285007317 | 0.371165644 | 0.181618398 | 1.476772509 |
| eca-miR-655 | 3.944747992 | 4.206287955 | 3.545205426 | 4.182670106 | 3.968897383 | 3.762101782 | 3.59850046 | 3.558177548 | 3.804875853 | 3.608542479 |
| eca-miR-8947 | 0 | 0.13646942 | 0 | 0 | 0.160061375 | 0 | 0 | 0 | 0 | 0 |
| eca-miR-421 | 2.920811158 | 1.750533338 | 3.298571192 | 1.854708839 | 3.030469071 | 2.662718342 | 3.365309963 | 3.797486712 | 2.897871864 | 2.636108524 |
| eca-miR-9123 | 2.492242592 | 3.705186878 | 2.591256409 | 3.671272863 | 1.700399657 | 3.076247969 | 3.917663465 | 4.811575496 | 3.839741572 | 3.422930387 |
| eca-miR-155 | 9.906417651 | 9.971001345 | 10.33385495 | 10.51807291 | 9.992236781 | 10.39775541 | 10.19975686 | 10.3699703 | 10.3304128 | 10.30468881 |
| eca-miR-615-3p | 3.81462076 | 4.316585391 | 3.943681757 | 4.494115534 | 4.430780384 | 3.961782456 | 3.745966548 | 3.871020969 | 4.635859332 | 4.230534524 |
| eca-miR-372 | 0.789255495 | 2.216376266 | 0.812525753 | 1.550128707 | 1.227610299 | 1.614894392 | 1.51373936 | 0.846900557 | 1.455867862 | 1.562867618 |
| eca-miR-188-5p | 2.709247815 | 3.271423012 | 3.1380562 | 2.013061382 | 3.126561967 | 3.117317995 | 3.591109795 | 3.141938731 | 3.365593251 | 2.962089374 |
| eca-miR-369-5p | 5.03691225 | 5.347263427 | 4.881995256 | 4.716763083 | 5.179248192 | 5.017019302 | 4.749551875 | 4.444096993 | 5.442823216 | 4.57007782 |
| eca-miR-1261 | 0.582766903 | 1.118513764 | 1.37035569 | 1.431579164 | 1.082674697 | 1.031967625 | 2.625668123 | 0.6486308 | 1.420475122 | 0.519408177 |
| eca-miR-335 | 7.446556125 | 7.776244976 | 7.365577408 | 7.800125119 | 7.707151274 | 7.691881797 | 7.704510376 | 7.594093392 | 8.274464331 | 7.434587775 |
| eca-miR-1301 | 2.516766045 | 3.127020609 | 3.305570539 | 2.676259653 | 2.750089376 | 3.287263563 | 1.855892386 | 3.483869294 | 3.335236519 | 3.790283959 |
| eca-miR-8908k | 6.96457787 | 5.954727395 | 6.748408681 | 6.547691362 | 6.296561028 | 6.935479262 | 7.506537472 | 6.479657966 | 5.588189003 | 6.458530587 |
| eca-miR-182 | 4.787384583 | 4.62393668 | 4.935471717 | 4.500757214 | 4.701036083 | 4.793014871 | 5.704379573 | 5.297515928 | 4.452185919 | 5.231497112 |
| eca-miR-381 | 7.749189271 | 8.439262258 | 7.639120584 | 8.160271317 | 7.736770915 | 8.038838124 | 7.98639552 | 8.084247382 | 8.890983325 | 7.933172433 |
| eca-miR-3200 | 4.896854988 | 5.015465714 | 4.708694295 | 4.591207021 | 4.998247732 | 5.090404854 | 4.984633116 | 4.598131855 | 4.897159904 | 4.651379954 |
| eca-miR-9036 | 2.26346427 | 0.746743955 | 2.558202329 | 1.981127347 | 1.788264647 | 0.92850514 | 2.113653126 | 3.924989658 | 1.378113948 | 2.758483612 |
| eca-miR-331 | 6.235617056 | 6.42026619 | 6.377287405 | 6.306219811 | 6.407479803 | 6.243281078 | 5.918652383 | 6.048624885 | 6.519353042 | 6.391053519 |
| eca-miR-449b | 5.998134362 | 4.659019855 | 6.662292522 | 6.167203791 | 6.795675827 | 6.266440015 | 5.864602356 | 6.657205958 | 3.840889722 | 6.651795756 |
| eca-miR-199b-5p | 12.26939062 | 12.89772488 | 12.38849644 | 12.51275043 | 12.66478581 | 13.22086331 | 13.7443272 | 12.84579333 | 13.23961287 | 13.5815736 |
| eca-miR-100 | 14.53764418 | 14.42277268 | 14.54611437 | 14.39838047 | 14.3164752 | 14.45402443 | 14.49436438 | 14.33007943 | 14.40196381 | 14.47339391 |
| eca-miR-9a | 0.484311259 | 0.129287481 | 0 | 0 | 0.316592858 | 0.276256195 | 0.743331975 | 0 | 0.093324621 | 0 |
| eca-miR-27a | 11.10732243 | 10.76122169 | 11.10335195 | 10.65118635 | 11.16425307 | 10.82400262 | 10.96630852 | 10.87255896 | 11.17656154 | 10.91500492 |
| eca-miR-9156 | 0 | 0 | 0.008769925 | 0 | 0 | 0 | 0 | 0 | 0 | 0 |
| eca-miR-376b | 3.890552289 | 4.526056652 | 3.524324832 | 4.613500139 | 3.461283962 | 4.264052541 | 4.038643469 | 4.104773458 | 4.991012388 | 3.565778702 |
| eca-miR-9058b | 3.435469149 | 4.336922349 | 2.077220131 | 4.295812483 | 3.095661947 | 3.986990627 | 3.022493544 | 4.468258403 | 3.915598964 | 3.208388838 |
| eca-miR-29a | 12.69981184 | 12.24745044 | 12.74263665 | 12.29984076 | 12.67209402 | 12.41211473 | 12.69720107 | 12.49805934 | 12.17949238 | 12.52783287 |
| eca-miR-9106 | 0.228779229 | 0.497027943 | 0.983788813 | 1.289386017 | 0.905072554 | 0.849284126 | 0.205530365 | 1.354138418 | 0.126146986 | 0.185375159 |
| eca-miR-374b | 9.044503349 | 8.244180547 | 8.997956997 | 8.817389937 | 8.80126855 | 8.569098843 | 8.597842123 | 9.256836338 | 7.982597485 | 9.285139661 |
| eca-miR-185 | 0 | 0.422520235 | 0 | 0 | 0 | 0.267490209 | 0 | 0 | 0.678289924 | 0 |
| eca-miR-18a | 6.315934263 | 5.343929576 | 6.370344542 | 6.138731211 | 6.409030865 | 6.029917314 | 6.025055288 | 6.068691606 | 5.328948286 | 6.304936017 |
| eca-miR-9086 | 1.809306191 | 1.048659861 | 1.478195785 | 0.761837889 | 1.544614913 | 0.631713595 | 0.680202214 | 2.530789528 | 1.356958629 | 1.393961336 |
| eca-miR-107b | 7.978514335 | 7.947060421 | 8.674634657 | 8.498036807 | 8.05801835 | 8.217258342 | 7.87069234 | 8.685809293 | 7.833065374 | 8.45853878 |
| eca-miR-501-5p | 6.628338705 | 5.992011254 | 6.797259069 | 6.405560212 | 6.284142651 | 5.747250318 | 5.873716433 | 6.564702994 | 7.096347316 | 6.78598628 |
| eca-miR-193a-3p | 0 | 0 | 0 | 0 | 0 | 0 | 0 | 0 | 0.338086709 | 0 |
| eca-miR-9132 | 0.238620884 | 0 | 0 | 0 | 0 | 0.029237315 | 0 | 0.411025864 | 0 | 0 |
| eca-miR-21 | 16.90869306 | 16.59627904 | 16.7956493 | 16.94086798 | 16.62708755 | 16.75586828 | 16.83201 | 16.80261658 | 16.64074964 | 16.83716256 |
| Eca-m0797-3p | 0 | 0 | 0 | 0 | 0.316270738 | 0.453645149 | 0.9477677 | 0.960764147 | 0 | 0 |
| Eca-m0390-5p | 0 | 0 | 0 | 0 | 0 | 0 | 0.157733201 | 0 | 0 | 0 |
| Eca-m0431-3p | 2.296755521 | 1.050079951 | 1.921536939 | 1.009081477 | 1.880216707 | 2.026112618 | 1.875245461 | 2.059309474 | 0.797410994 | 1.756477302 |
| Eca-m0398-3p | 0 | 0 | 0 | 0.208445264 | 0 | 0 | 0 | 0.260438511 | 0 | 0 |
| Eca-m0017-5p | 0.419256384 | 0 | 0.738690816 | 0 | 0 | 0 | 0 | 0.604603251 | 0 | 0.433887447 |
| Eca-m0189-5p | 1.20967103 | 0.721445599 | 1.283521867 | 1.338759807 | 1.201471131 | 0.986112984 | 1.11764832 | 1.392104881 | 1.096364609 | 0.552327059 |
| Eca-m0223-3p | 6.033089328 | 5.78096763 | 5.696947546 | 5.945135068 | 5.665205799 | 6.211960951 | 5.998244791 | 5.678404786 | 4.820070983 | 5.722256246 |
| Eca-m0799-3p | 2.742301121 | 1.412607938 | 1.928783077 | 2.206436901 | 2.120304387 | 1.338538913 | 0.875609388 | 2.07112012 | 1.090842001 | 0.839982199 |
| Eca-m0470-5p | 2.260206676 | 0.098371657 | 0.305751749 | 0.325266683 | 0.213020787 | 0.981927413 | 1.356538635 | 1.763565827 | 0.679529288 | 0.215646448 |
| Eca-m0088-3p | 0.96164887 | 0 | 0.084563397 | 0.096815065 | 0.895221324 | 0.019875532 | 0.645890654 | 0.924687244 | 0 | 0.905165738 |
| Eca-m0524-5p | 0.642639754 | 0 | 0.715211877 | 0.025452701 | 0.465608412 | 0.422697637 | 0 | 0.821808346 | 0 | 0.472556443 |
| Eca-m0326-5p | 0.378595083 | 0 | 0.454571614 | 0 | 0 | 0 | 0.283279589 | 0.834960829 | 0 | 0 |
| Eca-m0469-3p | 1.406687336 | 1.648939743 | 0.812807233 | 1.771842115 | 0.737628471 | 0.725232278 | 1.663637238 | 0.845820765 | 1.517675944 | 1.403475582 |
| Eca-m0622-3p | 0.242505287 | 0.124382025 | 0.995924483 | 0.275540365 | 0.916971397 | 1.255191838 | 1.4723793 | 0.289150807 | 0.138869975 | 1.083835149 |
| Eca-m0025-3p | 6.760262814 | 6.628286779 | 6.461147292 | 6.381501162 | 6.057433783 | 6.506997535 | 6.370606242 | 6.674774207 | 6.641668837 | 6.720731965 |
| Eca-m0444-5p | 0.732013687 | 1.103024401 | 3.648084566 | 0.803807975 | 1.51512842 | 2.48285502 | 2.164451286 | 2.054548794 | 0.469711146 | 0.629771292 |
| Eca-m0389-3p | 0.295368829 | 0 | 0.364645463 | 0 | 0 | 0.248809172 | 0 | 0 | 0 | 0.134249394 |
| Eca-m0411-3p | 1.346339609 | 0.493189129 | 0.688170525 | 0.707795479 | 2.051577752 | 1.680461519 | 2.122724352 | 1.806806586 | 1.21599997 | 0.600249636 |
| Eca-m0810-3p | 5.31382703 | 3.05632609 | 3.805851451 | 4.121942331 | 5.007460895 | 4.399226667 | 4.005556141 | 4.994607663 | 2.374124516 | 4.597132457 |
| Eca-m0550-3p | 2.034202869 | 1.474627559 | 1.904767631 | 1.964817814 | 1.801638291 | 2.312036627 | 1.91611364 | 2.630375466 | 1.763631363 | 2.20761285 |
| Eca-m0462-3p | 1.343056747 | 0 | 0.474850611 | 0 | 0 | 0.643649781 | 0 | 0 | 0 | 0 |
| Eca-m0047-3p | 0 | 0 | 0 | 0 | 0.166643261 | 0.126781052 | 0.434800173 | 0 | 0 | 0.487461996 |
| Eca-m0021-3p | 0.008732397 | 0 | 0 | 0.131648512 | 0 | 0 | 0 | 0 | 0.088219207 | 0 |
| Eca-m0577-3p | 0.137050837 | 0.295160316 | 0.858215229 | 1.149494766 | 0.100346437 | 0.561330162 | 0.118026654 | 0.965589645 | 0.360578584 | 0.612109933 |
| Eca-m0458-5p | 0 | 0 | 0 | 0.164900841 | 0.051489744 | 0 | 0 | 0 | 0 | 0 |
| Eca-m0097-5p | 0.064107085 | 0.465787042 | 1.321588419 | 1.188052273 | 0.016036605 | 0.544209993 | 0.039492419 | 1.254751563 | 0 | 0.01791267 |
| Eca-m0688-3p | 1.323248008 | 0.163078375 | 1.001252058 | 0.302699975 | 0.742400162 | 0.697639497 | 1.031809272 | 0.315577402 | 0.176134268 | 1.218457536 |
| Eca-m0124-5p | 0.706185591 | 0.233162674 | 0.088276442 | 0.098940736 | 0.705735602 | 0.488967429 | 0.809863413 | 0.122177719 | 0 | 0.992549954 |
| Eca-m0181-5p | 0 | 0 | 0 | 0 | 0 | 0 | 0 | 0.149521477 | 0 | 0 |
| Eca-m0438-3p | 0 | 0.122189576 | 0.5394665 | 0 | 0.305915419 | 0.423030353 | 0 | 0 | 0.086979871 | 0.480132971 |
| Eca-m0752-3p | 0.556971645 | 0.105491051 | 0 | 0.678559706 | 0.55753178 | 0 | 0 | 0.000760622 | 0.1645263 | 0.395259558 |
| Eca-m0048-3p | 3.739024771 | 2.080295073 | 3.416161419 | 1.686059416 | 3.408785816 | 2.919327028 | 2.338388245 | 3.254043074 | 2.14635648 | 2.942248328 |
| Eca-m0292-5p | 0 | 0 | 0 | 0 | 0.187993246 | 0 | 0 | 0.354311967 | 0 | 0.022669926 |
| Eca-m0574-3p | 0 | 0 | 0 | 0 | 0 | 0.099714882 | 0 | 0.317492797 | 0 | 0 |
| Eca-m0392-3p | 1.408671692 | 2.216622663 | 2.136075415 | 3.077147786 | 2.535835693 | 3.041011508 | 2.140360195 | 2.714022738 | 1.771743078 | 2.96549693 |
| Eca-m0769-3p | 2.465243845 | 0.664691804 | 1.850132319 | 0.906379135 | 1.932119224 | 1.453950497 | 1.632213613 | 2.198961845 | 1.035740146 | 1.332294923 |
| Eca-m0126-3p | 0 | 0 | 0 | 0 | 0.326976144 | 0 | 0.243731693 | 0 | 0 | 0.334648082 |
| Eca-m0441-5p | 1.37844264 | 1.046022932 | 1.457542745 | 0.766607322 | 1.183541061 | 1.309923404 | 1.279200774 | 2.352855812 | 0.91342051 | 1.531916963 |
| Eca-m0166-3p | 0 | 0 | 0 | 0.119010487 | 0 | 0 | 0 | 0 | 0 | 0 |
| Eca-m0818-3p | 3.243970823 | 1.294344205 | 2.993479713 | 2.91287632 | 2.896139514 | 3.426044979 | 3.550479926 | 4.034835141 | 1.715145789 | 3.087484812 |
| Eca-m0457-3p | 4.704261514 | 3.567637535 | 4.342664174 | 4.049669134 | 4.122126064 | 4.21859809 | 3.442111726 | 4.327564831 | 3.212367374 | 3.885352224 |
| Eca-m0291-3p | 0.930913225 | 0.429112523 | 0.295700443 | 0.309014713 | 1.080101335 | 1.1448371 | 0.256152247 | 1.367089 | 0.990790166 | 0.23733984 |
| Eca-m0534-3p | 0 | 0.410219066 | 0 | 1.039374609 | 0 | 0.242842764 | 0 | 0 | 0.043295461 | 0.293274614 |
| Eca-m0294-5p | 1.686642425 | 1.056036787 | 0.87116659 | 2.038890622 | 1.740698719 | 1.405319575 | 1.73491119 | 1.665677883 | 1.144126128 | 1.291606039 |
| Eca-m0195-3p | 0.861094899 | 0.391766654 | 0.918717726 | 0.960082818 | 4.559310563 | 3.258363032 | 2.889429065 | 0.998690578 | 0.466084893 | 4.103719689 |
| Eca-m0648-3p | 0.587352689 | 0.127971525 | 0 | 0.70958303 | 0.587866091 | 0.537345401 | 0 | 0.019655768 | 0 | 0.423611264 |
| Eca-m0520-5p | 2.362870618 | 0.828861162 | 1.819829276 | 0.782980611 | 1.781874357 | 0.613044587 | 0.67799354 | 3.020942767 | 0.922614958 | 1.794255988 |
| Eca-m0285-5p | 0 | 0 | 0 | 0 | 0 | 0.043571408 | 0.157377942 | 0 | 0 | 0 |
| Eca-m0537-5p | 3.274930912 | 1.724415391 | 3.296713877 | 2.733543026 | 3.668187102 | 2.618489676 | 1.572278689 | 4.517320436 | 2.58883089 | 3.685201251 |
| Eca-m0595-5p | 0 | 0 | 0 | 0 | 0 | 0 | 0 | 0.210022243 | 0 | 0 |
| Eca-m0621-3p | 0 | 0.259011487 | 0 | 0 | 0.357009531 | 0.600203006 | 0 | 0 | 0.344216555 | 0.525765608 |
| Eca-m0597-5p | 2.172367149 | 0.551072813 | 1.978141192 | 0.800331158 | 1.239204353 | 1.667489018 | 1.342431682 | 2.467165054 | 0.576545695 | 1.445013458 |
| Eca-m0722-3p | 0.439700079 | 0 | 0 | 0 | 0 | 0 | 0 | 0 | 0 | 0 |
| Eca-m0075-5p | 0.706634956 | 0.645401083 | 0.103038869 | 0.113190275 | 0.849281355 | 0.051115619 | 0.07336102 | 0.880408731 | 0.519798144 | 0.059516781 |
| Eca-m0555-5p | 1.504698976 | 0.244753701 | 1.608806069 | 0.429571632 | 0.334938412 | 1.717186691 | 0.361801296 | 1.283140884 | 0.613836231 | 0.897634838 |
| Eca-m0718-3p | 0 | 0.574490045 | 0 | 0.914532589 | 0 | 0.096740032 | 0 | 0 | 0 | 0 |
| Eca-m0439-5p | 3.634295442 | 2.677597786 | 3.394119053 | 3.067581829 | 3.474570585 | 2.538481576 | 3.297034474 | 2.722738919 | 3.369351714 | 3.142656902 |
| Eca-m0673-5p | 0.837213303 | 0.314208187 | 0.9267819 | 0 | 0 | 0 | 0 | 0.797698088 | 0 | 0.43633284 |
| Eca-m0743-5p | 8.105945801 | 6.014457584 | 7.688485579 | 7.239880672 | 7.832959404 | 7.578817057 | 6.413296399 | 7.978001696 | 5.894540077 | 6.78909256 |
| Eca-m0130-3p | 4.185953895 | 3.200054133 | 4.182718225 | 3.904685982 | 4.517746039 | 3.657913862 | 4.423435011 | 3.392294628 | 4.251539963 | 3.461896377 |
| Eca-m0269-5p | 1.937948518 | 1.856627667 | 2.221565718 | 1.137054971 | 2.105753291 | 1.917639718 | 1.61951535 | 2.690409716 | 1.239652961 | 1.711251546 |
| Eca-m0538-3p | 1.442459681 | 0.415340052 | 1.727292562 | 0.597349898 | 1.728362351 | 1.305366334 | 0.529653112 | 1.414120659 | 0.761968891 | 1.036314218 |
| Eca-m0635-5p | 0.940046254 | 1.347432889 | 0.972580192 | 1.771870223 | 2.56351226 | 2.059098841 | 1.516723561 | 2.769039107 | 1.112181555 | 1.919273019 |
| Eca-m0708-3p | 2.216895607 | 1.607208671 | 2.084852773 | 2.149177254 | 3.25148918 | 1.905773541 | 2.260293237 | 2.837005402 | 1.827192847 | 2.985506916 |
| Eca-m0652-3p | 0.369825424 | 0.115661775 | 0 | 0 | 0 | 0 | 0 | 0 | 0 | 0.007346435 |
| Eca-m0633-5p | 1.879322434 | 0.83378787 | 0.738921891 | 0.755442149 | 1.481127942 | 1.277750275 | 1.441450615 | 1.530766855 | 1.30755508 | 1.491042328 |
| Eca-m0669-5p | 1.278942055 | 0.306282678 | 1.138981717 | 0.454738562 | 1.20929447 | 0.835385481 | 1.166778834 | 1.249154703 | 0.320170159 | 0.887315892 |
| Eca-m0151-5p | 0.074480591 | 0 | 0 | 0 | 0 | 0 | 0 | 0 | 0 | 0 |
| Eca-m0056-5p | 1.442113133 | 0.830764458 | 0.068478762 | 1.801580089 | 0 | 0 | 0.016601713 | 1.024440703 | 0 | 0 |
| Eca-m0600-5p | 0.50696141 | 0.761423925 | 2.17115042 | 1.346437011 | 1.19787898 | 0.431817044 | 0.475951745 | 1.404220138 | 0.722178393 | 1.634986363 |
| Eca-m0644-5p | 0.551141624 | 0.071320366 | 0 | 0 | 0.704145265 | 0.98002231 | 0 | 0 | 0 | 0.381337654 |
| Eca-m0192-3p | 0 | 0 | 0.724539341 | 0.79184931 | 0 | 0 | 0.308418421 | 0 | 0 | 0.224558782 |
| Eca-m0102-3p | 2.15693112 | 1.043047984 | 1.783519696 | 1.612071758 | 1.278834763 | 1.676811413 | 1.740061932 | 0.892513343 | 0.687027817 | 1.757265856 |
| Eca-m0095-3p | 0 | 0 | 0 | 0 | 0 | 0.03440014 | 0 | 0 | 0 | 0 |
| Eca-m0714-3p | 2.226222246 | 0.93249882 | 1.83850461 | 1.659398815 | 1.922099816 | 1.438403488 | 0.793368383 | 2.516609713 | 1.149656953 | 0.76075895 |
| Eca-m0646-3p | 0.622669846 | 0 | 0 | 0 | 0.559681235 | 0 | 0 | 0.580389051 | 0 | 0.22434279 |
| Eca-m0168-3p | 0 | 0 | 0.769443744 | 0 | 0.61841522 | 0.557875681 | 0.369849488 | 0 | 0 | 0 |
| Eca-m0084-3p | 0.280938609 | 0 | 0 | 0 | 0.072757998 | 0 | 0 | 0 | 0 | 0.080965387 |
| Eca-m0086-5p | 1.973310494 | 0.51274628 | 1.372250909 | 0.69060463 | 1.28532204 | 1.374760663 | 1.198289215 | 1.485310851 | 0.844662205 | 1.294287398 |
| Eca-m0404-5p | 0 | 0 | 0.393662138 | 0 | 0 | 0 | 0 | 0 | 0 | 0 |
| Eca-m0557-5p | 2.507617398 | 2.324419522 | 2.056248811 | 1.42041524 | 1.946684497 | 2.457404738 | 2.225459579 | 2.781858371 | 2.41052853 | 2.35347128 |
| Eca-m0304-3p | 0 | 0.388864705 | 0 | 0 | 0.014633804 | 0 | 0.092618525 | 0 | 0 | 0 |
| Eca-m0725-5p | 3.170096013 | 1.072345869 | 2.122638356 | 2.436916506 | 2.89018997 | 3.263803293 | 2.452138364 | 2.509594451 | 1.111358779 | 2.905128915 |
| Eca-m0806-5p | 2.341270686 | 1.530281894 | 2.018203062 | 1.329276691 | 3.260373067 | 1.837219814 | 2.59742683 | 3.073377214 | 1.530703288 | 2.548352786 |
| Eca-m0009-3p | 0.583016311 | 1.114517502 | 0.601688931 | 0.615789152 | 1.329735755 | 1.132314016 | 1.101415377 | 0.629474505 | 1.163450601 | 1.021207052 |
| Eca-m0471-3p | 5.320977259 | 3.668558945 | 5.269141998 | 5.733274801 | 5.087622668 | 5.070252328 | 4.935682751 | 5.81821056 | 3.571664295 | 5.400260681 |
| Eca-m0348-5p | 0.246341574 | 0 | 0.317166138 | 0 | 0.073991848 | 0 | 0.354558791 | 0 | 0 | 0 |
| Eca-m0272-5p | 2.109894488 | 0.679781094 | 2.366935137 | 0.922498963 | 1.686186898 | 1.868442475 | 1.645541061 | 2.002981726 | 0.704150251 | 1.696873567 |
| Eca-m0553-3p | 3.741779623 | 2.180232595 | 3.014264726 | 2.936967799 | 3.478875739 | 2.521583228 | 2.871802084 | 3.461173102 | 2.47908071 | 2.932751319 |
| Eca-m0296-3p | 1.046214325 | 0.277297971 | 1.121931692 | 0.428408539 | 1.325685493 | 0.986593937 | 0.951514757 | 1.484346527 | 0.606564628 | 0.350863529 |
| Eca-m0687-3p | 0 | 0.618311426 | 0 | 0 | 0 | 0 | 0 | 0 | 0.383245376 | 0 |
| Eca-m0259-3p | 3.627772212 | 3.469738595 | 2.672531737 | 2.738701126 | 3.280441003 | 3.263209306 | 3.280473726 | 3.650588575 | 3.664353228 | 3.368214725 |
| Eca-m0785-5p | 3.150737249 | 2.21238177 | 2.855835794 | 2.945709957 | 1.826705939 | 2.45037121 | 1.93380408 | 2.882456031 | 1.650409768 | 2.022146268 |
| Eca-m0695-3p | 0.42917505 | 0.571960717 | 1.425069784 | 1.244796108 | 1.396624657 | 0.86682622 | 0.402164019 | 1.767402016 | 0.309167358 | 0.921975043 |
| Eca-m0246-3p | 2.503720996 | 2.374028221 | 2.766672717 | 2.850436536 | 2.587382508 | 2.29673533 | 2.369245924 | 2.930623217 | 1.852901743 | 2.495837269 |
| Eca-m0581-3p | 2.471584159 | 2.104438299 | 2.42588767 | 2.503698649 | 2.305164366 | 1.992426657 | 2.32888121 | 2.379369602 | 1.126681788 | 2.20047223 |
| Eca-m0478-3p | 0.02351083 | 0 | 0 | 0 | 0 | 0 | 0 | 0 | 0 | 0 |
| Eca-m0679-5p | 0.831843163 | 0 | 0.485647717 | 0 | 0.222738786 | 0 | 0 | 0.596566756 | 0 | 0 |
| Eca-m0367-5p | 0.711234266 | 0.224013848 | 0 | 0 | 0 | 0.287495444 | 0.604660153 | 0 | 0 | 0.510467846 |
| Eca-m0074-5p | 1.603752364 | 0.84977571 | 1.464440544 | 0.777677291 | 2.144733062 | 1.143983457 | 1.487091058 | 1.57961865 | 0.600528474 | 1.672342823 |
| Eca-m0173-3p | 0.339039959 | 0 | 0.657862086 | 0 | 0.155146989 | 0 | 0 | 0.523313205 | 0 | 0 |
| Eca-m0667-5p | 0 | 0.132631485 | 0 | 0 | 0 | 0 | 0 | 0 | 0 | 0 |
| Eca-m0108-3p | 0.662291001 | 0.469433582 | 0.730605879 | 0.0901091 | 0.496811991 | 0.612140748 | 0.054363089 | 0.111573596 | 0.385761868 | 0.042048636 |
| Eca-m0423-5p | 1.336655965 | 0.172493511 | 0.300642778 | 0.314144907 | 0.754293287 | 1.153611978 | 0.844618885 | 1.124220113 | 0.18576284 | 0.944465238 |
| Eca-m0055-5p | 6.782636387 | 6.972966902 | 6.81040148 | 6.582780991 | 6.921661203 | 7.097314091 | 6.972227617 | 6.692630997 | 6.916891078 | 6.705817645 |
| Eca-m0248-3p | 1.573927075 | 0.515353602 | 0.389253771 | 0.403511142 | 1.303843612 | 0.792841557 | 0.346810598 | 1.210031509 | 0.707622708 | 1.027830672 |
| Eca-m0359-3p | 0.221409654 | 0 | 0 | 0 | 0 | 0 | 0 | 1.296761346 | 0 | 0 |
| Eca-m0528-5p | 2.941598775 | 1.519420058 | 1.071936299 | 1.097605696 | 2.122572917 | 2.562802173 | 2.145136894 | 2.188916768 | 0.83739458 | 2.011153314 |
| Eca-m0211-5p | 2.301475683 | 1.534874805 | 1.939136944 | 1.774340791 | 1.768668461 | 1.564249029 | 1.893440411 | 1.086138785 | 1.301251982 | 1.908702105 |
| Eca-m0802-5p | 5.697515257 | 4.224240449 | 4.618463493 | 5.291640189 | 4.631721498 | 4.242741817 | 4.766010337 | 4.761489955 | 4.378089104 | 4.535692616 |
| Eca-m0204-3p | 5.418388113 | 3.779016286 | 5.144808456 | 4.516237547 | 5.005233795 | 4.964404169 | 4.693082459 | 5.373574246 | 3.318221961 | 5.192002394 |
| Eca-m0741-3p | 0 | 0 | 0 | 0 | 0 | 0 | 0 | 0.056000562 | 0 | 0 |
| Eca-m0080-5p | 0 | 0 | 0.05821834 | 0 | 0 | 0 | 0 | 0 | 0 | 0 |
| Eca-m0631-5p | 0 | 0 | 1.120371431 | 0 | 0.19960518 | 0 | 0 | 0 | 0 | 0.976616571 |
| Eca-m0284-3p | 7.350353541 | 5.524510324 | 6.973852619 | 6.543875836 | 6.339535629 | 5.086119927 | 3.764297967 | 5.745183252 | 4.697498228 | 5.621267211 |
| Eca-m0067-5p | 7.571085441 | 5.153739508 | 6.972182369 | 6.806727127 | 6.675427303 | 6.178470104 | 5.746882499 | 6.734167462 | 4.954368194 | 6.243228386 |
| Eca-m0231-3p | 0 | 0 | 0 | 0 | 0.152386106 | 0 | 0 | 1.037749393 | 0 | 0 |
| Eca-m0178-3p | 0.735853798 | 0 | 0 | 0 | 0.174301541 | 0.132881073 | 0.258042662 | 0 | 0 | 0 |
| Eca-m0827-3p | 1.087630071 | 2.278458775 | 1.114809701 | 1.135234088 | 2.264513417 | 1.764906756 | 2.212944944 | 1.892297426 | 1.797023593 | 1.513828402 |
| Eca-m0510-5p | 0.34371906 | 0 | 0.651270852 | 0 | 0.348658636 | 0 | 0.253234372 | 0 | 0 | 0 |
| Eca-m0554-5p | 2.514398974 | 0.67486388 | 0.586968798 | 0.613541973 | 0.457518113 | 1.855139338 | 0.50367238 | 3.148877404 | 1.205446473 | 1.1301427 |
| Eca-m0428-5p | 0.407515929 | 1.009363554 | 0.423862514 | 0.436250855 | 1.281869266 | 0.36041602 | 0.387620938 | 1.196348964 | 1.204184213 | 0.849662368 |
| Eca-m0741-5p | 1.934765401 | 1.188942545 | 2.341053179 | 0.928382179 | 1.518527744 | 1.608924787 | 1.43232185 | 2.189856885 | 1.570397549 | 0.81602057 |
| Eca-m0587-3p | 0.678481213 | 0 | 0.984978998 | 0.048666839 | 0.4983924 | 0.770930955 | 0.00160215 | 0.075713563 | 0 | 0.841381962 |
| Eca-m0247-5p | 0.580097502 | 0.309003764 | 0.649440154 | 0.000525434 | 0 | 0.371911399 | 0.494053314 | 0.024937971 | 0.487370046 | 0 |
| Eca-m0050-5p | 1.0536445 | 0.023800404 | 0.157043299 | 0.170710939 | 0.637894764 | 0.591540286 | 0.938492523 | 1.489049334 | 0.376069669 | 0.096295933 |
| Eca-m0793-3p | 4.876284805 | 4.002696344 | 4.869938394 | 5.198181075 | 6.203457197 | 6.447746548 | 5.622573906 | 4.960921661 | 3.904115008 | 4.77302068 |
| Eca-m0229-3p | 0.229469195 | 0 | 0 | 0.358018106 | 0 | 0.00494127 | 0 | 0.410927874 | 0 | 0 |
| Eca-m0057-3p | 0 | 0 | 1.070104141 | 0 | 0 | 0 | 0.097234095 | 0 | 0 | 0.009736454 |
| Eca-m0521-5p | 0 | 0.098160587 | 0 | 0.687890771 | 0.839315307 | 0.513626226 | 0 | 0 | 0.159632748 | 0.398004519 |
| Eca-m0268-5p | 1.691739683 | 0.641423076 | 0.527460501 | 0.542510374 | 0.963327511 | 1.087341022 | 1.054121054 | 1.583988091 | 0.938695746 | 0.970629673 |
| Eca-m0093-3p | 1.949625296 | 0.84335313 | 1.470407788 | 2.142134464 | 1.789248743 | 1.320950015 | 1.290038081 | 0.790999074 | 0.587314702 | 1.20092738 |
| Eca-m0675-3p | 7.090756192 | 5.274008942 | 6.464808405 | 6.06604387 | 6.515587003 | 5.768529272 | 4.850811584 | 6.578833334 | 4.809945399 | 5.441628157 |
| Eca-m0035-3p | 4.714935547 | 2.88207086 | 4.045581915 | 4.307310698 | 4.291851549 | 3.360201545 | 3.789823442 | 4.163162258 | 2.667318864 | 3.683940043 |
| Eca-m0381-5p | 0 | 0 | 0.359683993 | 0 | 0 | 0 | 0 | 0 | 0 | 0 |
| Eca-m0399-3p | 7.664155116 | 5.100370543 | 6.842772796 | 6.086307362 | 6.87220971 | 5.768294964 | 4.950380741 | 6.30948431 | 4.387843992 | 6.487227304 |
| Eca-m0699-5p | 2.669916221 | 1.220831754 | 2.411305489 | 2.323800975 | 1.496799952 | 1.786712449 | 0.979565889 | 2.734218383 | 0.817570262 | 2.00979777 |
| Eca-m0020-3p | 0.892028345 | 0.379073608 | 1.20463248 | 0.253405205 | 0.17798856 | 1.328416412 | 0.199135105 | 0.266528026 | 0.448731976 | 1.310471644 |
| Eca-m0196-5p | 0 | 0 | 0.517992834 | 0 | 0.220861124 | 0 | 0 | 0 | 0 | 0.229198804 |
| Eca-m0787-5p | 1.189916208 | 0.592451454 | 0.365485121 | 1.106721839 | 0.974151219 | 1.185050845 | 1.080046115 | 0.390822998 | 0.55678844 | 0.309560135 |
| Eca-m0026-3p | 1.419565176 | 0.892119446 | 1.697027203 | 0.601824544 | 1.478586325 | 1.286060025 | 1.306410152 | 0.616875683 | 0.45389782 | 1.025965322 |
| Eca-m0499-5p | 5.497736669 | 3.410912277 | 5.393981798 | 3.535506935 | 4.092638014 | 5.63045717 | 5.532769425 | 4.710727295 | 3.704948927 | 6.309998749 |
| Eca-m0527-5p | 0.496156909 | 0 | 0 | 0 | 0.642819842 | 0.447211301 | 0 | 0 | 0.214034456 | 0.505814174 |
| Eca-m0578-3p | 0.765988966 | 0 | 0.858869908 | 0 | 0.336300939 | 0 | 0 | 0.993845653 | 0 | 0 |
| Eca-m0430-5p | 0 | 0 | 0 | 0 | 0.11508428 | 0 | 0 | 0 | 0 | 0 |
| Eca-m0385-5p | 1.131932029 | 2.516963392 | 2.317362724 | 1.193580065 | 1.775638308 | 1.713761677 | 1.087506688 | 2.010194314 | 1.972676596 | 1.594070064 |
| Eca-m0155-3p | 0 | 0.380690686 | 0.383522959 | 0 | 0.151023521 | 0 | 0 | 0 | 0 | 0.15742041 |
| Eca-m0682-3p | 0.492477496 | 0 | 0 | 0 | 0 | 0 | 0.376570229 | 0.717910642 | 0 | 0 |
| Eca-m0643-5p | 2.636791978 | 1.617579554 | 1.903475521 | 1.221771866 | 2.20970018 | 2.572378748 | 1.11783468 | 2.810838809 | 1.66028979 | 2.330595867 |
| Eca-m0077-3p | 1.16852859 | 0 | 0.838393811 | 0.120296394 | 0.763169548 | 1.099073493 | 0.070645749 | 0.143003524 | 0.000932789 | 0.585847783 |
| Eca-m0753-5p | 1.547070895 | 0.543146902 | 1.408494862 | 1.706677018 | 1.721503086 | 1.409866015 | 0.658088826 | 1.52213323 | 0.877617526 | 1.1476235 |
| Eca-m0072-3p | 1.91732907 | 0.854091278 | 1.52248284 | 0.535944475 | 1.825143992 | 0.944065176 | 0.461171505 | 1.660891719 | 0.350818029 | 0.433738209 |
| Eca-m0592-5p | 1.382237178 | 0.815972411 | 0.725804991 | 0.745895275 | 2.08686595 | 1.311321988 | 1.488284414 | 2.228310517 | 0.547563013 | 1.912604287 |
| Eca-m0703-5p | 4.78199139 | 3.074658025 | 3.64103505 | 4.171712655 | 4.50076161 | 3.89937145 | 3.457768484 | 5.256697339 | 3.271891619 | 4.484646051 |
| Eca-m0513-5p | 3.915045418 | 2.805989031 | 4.363776022 | 3.587825772 | 4.968079621 | 4.238091671 | 3.732005793 | 4.768884096 | 3.450192933 | 4.473224065 |
| Eca-m0046-3p | 0 | 0 | 0 | 0 | 0 | 0 | 0 | 0 | 0 | 0.175899501 |
| Eca-m0122-5p | 1.580805461 | 0.380152185 | 0.963808705 | 0.254872132 | 0.702935528 | 0.657757799 | 0.995594579 | 0.267759726 | 0.448943336 | 0.182659936 |
| Eca-m0516-3p | 0 | 0 | 0 | 0 | 0.132454601 | 0.254505558 | 0 | 0.473711528 | 0 | 0.139044686 |
| Eca-m0105-3p | 2.60127804 | 1.616110263 | 2.115702063 | 1.231202289 | 2.385174986 | 2.11041471 | 2.065718813 | 2.254021518 | 1.322037181 | 2.079847867 |
| Eca-m0198-3p | 0.695448066 | 0.32197866 | 0.992927707 | 0.087403679 | 0.695132357 | 0.643296075 | 0.045525179 | 0.11136239 | 0 | 0.527515081 |
| Eca-m0507-5p | 0.099338935 | 0 | 0 | 0 | 0 | 0 | 0.014552442 | 0 | 0 | 0.258094241 |
| Eca-m0230-5p | 5.69318078 | 3.338072864 | 5.502638627 | 4.34207702 | 4.67788866 | 5.223153121 | 5.088281094 | 4.972035934 | 3.899825325 | 4.72995837 |
| Eca-m0531-5p | 1.692003892 | 1.162451452 | 1.559376073 | 2.037800648 | 1.958809133 | 1.414306247 | 0.84037133 | 0.925857762 | 1.591058935 | 1.302475929 |
| Eca-m0767-5p | 0.814983389 | 1.623919949 | 0.140128365 | 0.153848329 | 0.077322096 | 1.158975371 | 0.098911645 | 0.173864523 | 1.031727346 | 0.079042596 |
| Eca-m0252-3p | 0.136200057 | 2.051067449 | 0.15712926 | 1.003407005 | 0.084938625 | 0.616082685 | 0.762902912 | 0.192134772 | 0.753333995 | 0.086943298 |
| Eca-m0380-5p | 1.737861366 | 1.11865517 | 1.427452659 | 0.764630402 | 1.489145763 | 1.551199375 | 1.449572573 | 1.539104451 | 0.607147909 | 1.172344603 |
| Eca-m0073-3p | 0 | 0 | 0 | 0 | 0 | 0 | 0 | 0 | 0 | 0.096834121 |
| Eca-m0297-3p | 1.230073471 | 0.776946811 | 1.824073596 | 0.362917499 | 0.278773983 | 0.267177726 | 1.114370263 | 0.377456549 | 0.89349929 | 0.822480617 |
| Eca-m0118-5p | 0 | 0 | 0 | 0 | 0.061148978 | 0 | 0 | 0.497910414 | 0 | 0 |
| Eca-m0055-3p | 4.716836987 | 5.632087688 | 5.331947309 | 4.894387246 | 5.51871906 | 5.113387464 | 6.02497827 | 5.715351801 | 5.226766925 | 5.160048765 |
| Eca-m0542-5p | 0.681070483 | 2.873686263 | 0.711968703 | 2.204827766 | 1.705845454 | 1.329917929 | 0.642061867 | 0.756528599 | 1.453548331 | 1.199218967 |
| Eca-m0317-5p | 9.117286464 | 9.433143045 | 9.420383283 | 9.404098384 | 9.192523547 | 9.74240157 | 9.542029395 | 9.429279924 | 9.610605481 | 9.506911 |
| Eca-m0111-3p | 1.975578491 | 2.567645888 | 2.061084833 | 1.401712179 | 2.646790833 | 1.885607637 | 2.235667604 | 1.428026145 | 1.900819632 | 2.112793976 |
| Eca-m0662-3p | 1.688627014 | 0.449494405 | 0.629589374 | 0.647819599 | 1.428696942 | 1.894708307 | 1.385584907 | 1.734040897 | 0.468946132 | 1.439115847 |
| Eca-m0396-3p | 0 | 0 | 0 | 0 | 0 | 0 | 0.099301266 | 0 | 0 | 0.009085767 |
| Eca-m0612-3p | 0.419483733 | 0 | 0.489939227 | 0 | 0.422190466 | 0 | 0 | 0 | 0.019642926 | 0.430426761 |
| Eca-m0781-5p | 0.42355754 | 0.673328441 | 0.443846244 | 0.459050604 | 1.079143832 | 1.41204515 | 1.358000238 | 1.527720983 | 0.313193323 | 1.378062792 |
| Eca-m0044-5p | 0.350492073 | 0 | 0 | 0 | 0 | 0 | 0 | 0 | 0 | 0 |
| Eca-m0405-5p | 1.070499509 | 0 | 0.570529994 | 0 | 0 | 0.263534929 | 0 | 0 | 0 | 0.669507678 |
| Eca-m0825-5p | 6.569199376 | 5.69856554 | 6.43068209 | 6.663621467 | 6.391845498 | 6.204254298 | 5.964791275 | 6.78171309 | 4.563531108 | 6.274541986 |
| Eca-m0220-5p | 0 | 0 | 1.124540799 | 0 | 0.769137263 | 0 | 0.510302911 | 0 | 0 | 0.918107115 |
| Eca-m0110-3p | 0 | 0 | 0 | 0.086834082 | 0 | 0 | 0 | 0 | 0 | 0 |
| Eca-m0408-3p | 0.284139013 | 0 | 0 | 0 | 0 | 0 | 0 | 1.190590453 | 0 | 0.30601207 |
| Eca-m0492-3p | 0 | 0 | 0 | 0 | 0 | 0.074209794 | 0 | 0 | 0 | 0.123225453 |
| Eca-m0740-3p | 0 | 0 | 0 | 0 | 0.352304372 | 0.125070696 | 0.627429488 | 0.527179828 | 0 | 0 |
| Eca-m0671-3p | 0 | 0 | 0 | 0 | 0 | 0 | 0.102321032 | 0 | 0 | 0 |
| Eca-m0310-3p | 3.637401369 | 3.62119878 | 3.972431993 | 3.972684859 | 4.00032981 | 4.082352923 | 3.471183122 | 2.464731852 | 3.972792899 | 4.016456735 |
| Eca-m0630-3p | 2.636620576 | 0.851472096 | 0.774975762 | 0.798570502 | 1.888781301 | 1.194092065 | 0.702656013 | 2.501403753 | 1.208754731 | 1.901474028 |
| Eca-m0005-3p | 0 | 0 | 0.241890012 | 0 | 0.324435071 | 0 | 0 | 0 | 0 | 0.185507766 |
| Eca-m0185-5p | 1.224389094 | 0.31263312 | 0.895864828 | 0.180855638 | 1.332034609 | 0.764763319 | 0.129389539 | 0.1972101 | 0.381443287 | 0.110960886 |
| Eca-m0512-3p | 0 | 0 | 0.412388887 | 0.466897333 | 0.161036538 | 0.566602808 | 0 | 0 | 0 | 0 |
| Eca-m0217-3p | 0 | 0 | 0.556451276 | 0 | 0 | 0.350383161 | 0.16544603 | 0 | 0 | 0 |
| Eca-m0311-5p | 1.357721653 | 2.94390598 | 1.40010543 | 3.404981791 | 2.420170785 | 2.861555132 | 2.296376667 | 1.461374654 | 3.433174052 | 1.809418591 |
| Eca-m0514-5p | 1.615689364 | 0.409191612 | 1.718013414 | 0.589261636 | 1.360272004 | 1.425596546 | 1.317417476 | 0.605427039 | 0.426561095 | 1.370489979 |
| Eca-m0819-5p | 0.447989848 | 0 | 0 | 0 | 0.240535871 | 0.026110763 | 0.149390366 | 0 | 0 | 0 |
| Eca-m0509-3p | 0.795024527 | 0.034225212 | 0.148709353 | 1.165980733 | 1.078350694 | 0.084700929 | 1.068027404 | 0.172861801 | 0.045832401 | 0.618818421 |
| Eca-m0365-3p | 3.46922514 | 1.612486384 | 2.785074911 | 2.430666772 | 3.712189405 | 3.425469663 | 2.357767714 | 3.269900844 | 2.252760548 | 2.95835524 |
| Eca-m0004-3p | 1.441787483 | 0.232949816 | 1.543189769 | 0.395976276 | 1.322916611 | 0.797467155 | 1.313180408 | 0.410662823 | 0.580281387 | 0.313133282 |
| Eca-m0475-3p | 1.154758624 | 0.62845483 | 1.232359789 | 0.529394087 | 1.148044955 | 1.240001954 | 0.465240723 | 1.805483835 | 0.374418475 | 1.44832895 |
| Eca-m0340-5p | 2.990329727 | 1.637949544 | 2.372940474 | 1.465252275 | 1.819771098 | 3.009288896 | 1.930150426 | 3.860342082 | 2.201449899 | 2.755348896 |
| Eca-m0374-5p | 3.650620508 | 1.020077937 | 1.363681341 | 1.395796396 | 2.617501344 | 2.85118204 | 2.430201721 | 2.698770887 | 2.945042516 | 2.52967153 |
| Eca-m0435-5p | 0.071414884 | 0 | 0 | 0 | 0 | 0 | 0 | 0 | 0 | 0 |
| Eca-m0422-5p | 2.600623223 | 1.578917072 | 2.353687574 | 2.270115518 | 1.824962404 | 1.437793646 | 1.587011107 | 1.119918912 | 1.33288548 | 1.835494682 |
| Eca-m0795-5p | 0 | 0 | 0 | 0 | 0.170136177 | 0.106251878 | 0 | 0 | 0 | 0 |
| Eca-m0037-3p | 0 | 0 | 0.020233722 | 0 | 0 | 0 | 0 | 0 | 0 | 0 |
| Eca-m0424-3p | 1.8623465 | 0.799396268 | 0.718155192 | 0.742142079 | 2.481699437 | 2.720584937 | 0.64429367 | 1.636735663 | 0.49231003 | 1.741570785 |
| Eca-m0654-3p | 0.965885249 | 0 | 0 | 0 | 0.406351406 | 0 | 0.309067827 | 0 | 0 | 0.227644797 |
| Eca-m0440-3p | 0.286728698 | 0 | 0 | 0 | 0.117146927 | 0.379720457 | 0.392437003 | 0 | 0 | 0 |
| Eca-m0779-5p | 3.389326064 | 2.392771613 | 1.94852971 | 2.723675817 | 4.550014322 | 3.783820716 | 3.937599459 | 5.324351761 | 2.792983496 | 4.005438651 |
| Eca-m0177-5p | 0.364266298 | 0 | 0 | 0 | 0.19158363 | 0.149865964 | 0 | 0.539909999 | 0 | 0.198343888 |
| Eca-m0616-5p | 0 | 0.129498158 | 0 | 0 | 0 | 0 | 0.040900805 | 0 | 0.114605464 | 0 |
| Eca-m0734-3p | 0.92345188 | 0.014760287 | 0.78716134 | 0.118792539 | 0.71540444 | 0.501832455 | 0.627924827 | 0.13696765 | 0.528016503 | 0.064019414 |
| Eca-m0582-5p | 3.540852776 | 3.288002543 | 3.261035436 | 1.988246517 | 3.46178338 | 3.244530143 | 3.366933349 | 2.938519443 | 3.027479555 | 2.280411977 |
| Eca-m0014-3p | 0 | 0 | 0 | 0 | 0.493789833 | 0.587867443 | 0 | 0.672205119 | 0 | 0.793823441 |
| Eca-m0426-5p | 1.172361639 | 0.956982583 | 0.587838051 | 0.601406198 | 1.16471465 | 1.249214998 | 1.26880724 | 1.351770441 | 1.062082212 | 1.002958654 |
| Eca-m0786-3p | 1.569701314 | 0.954968177 | 1.435423871 | 0.776946164 | 1.840457335 | 1.294300501 | 1.457196577 | 1.546653673 | 0.921612667 | 1.18150522 |
| Eca-m0503-5p | 0 | 0 | 0 | 0 | 0.095169369 | 0 | 0 | 0.260933951 | 0 | 0 |
| Eca-m0148-3p | 0 | 0 | 0 | 0.798497597 | 0.963849788 | 0.612168561 | 0 | 1.116144332 | 0.219552486 | 0 |
| Eca-m0010-5p | 0.210002138 | 0 | 0.278233785 | 0 | 0.044894388 | 0.005216124 | 0 | 0 | 0 | 0 |
| Eca-m0750-5p | 0.012440698 | 0 | 0.028198818 | 0.815729161 | 0.501996962 | 1.020523693 | 0 | 0.869392761 | 0 | 0.695211364 |
| Eca-m0263-3p | 3.205080702 | 1.745417118 | 2.832002571 | 2.137328367 | 2.014935601 | 2.170895908 | 0.969698926 | 1.109464335 | 0.793690219 | 1.712792333 |
| Eca-m0733-5p | 4.269028912 | 2.564498666 | 4.281153572 | 3.441516466 | 3.368244595 | 2.966360816 | 1.634388616 | 2.861704916 | 1.753736571 | 3.569615967 |
| Eca-m0636-5p | 1.628213292 | 0.192885876 | 0.709973393 | 0 | 0 | 0 | 0 | 0 | 0 | 0.96409962 |
| Eca-m0483-5p | 1.59780954 | 0.054489446 | 0.959286066 | 0.214382187 | 0.880103749 | 1.332725111 | 0.155431924 | 0.228509693 | 0.070122892 | 0.695407121 |
| Eca-m0415-3p | 0 | 0 | 0.024082832 | 0 | 0 | 0 | 0 | 0.103759682 | 0 | 0 |
| Eca-m0300-3p | 2.989941421 | 2.032341272 | 2.993025653 | 2.773483167 | 3.209007032 | 2.764538251 | 2.075770975 | 3.300047014 | 1.784334294 | 2.572356572 |
| Eca-m0199-3p | 0 | 0.213789688 | 0 | 0 | 0.495152823 | 0.707385114 | 0 | 0.66894619 | 0.204052547 | 0 |
| Eca-m0045-5p | 0 | 0 | 0.562299904 | 0 | 0 | 0.200535003 | 0 | 0.428912955 | 0 | 0 |
| Eca-m0814-5p | 0 | 0 | 0.039548925 | 0 | 0 | 0 | 0 | 0 | 0 | 0 |
| Eca-m0043-5p | 0.647982572 | 0 | 0.500782743 | 0 | 0.585126246 | 0.378969586 | 0 | 0 | 0 | 0 |
| Eca-m0447-3p | 1.51621636 | 0.436346549 | 1.033143432 | 1.09029865 | 1.109706979 | 0.225787916 | 0.259067462 | 0.330246239 | 0.178013501 | 1.11981186 |
| Eca-m0171-3p | 0 | 0 | 1.449198949 | 0.037823283 | 1.177526548 | 0.8420044 | 0 | 0.933621687 | 0 | 0 |
| Eca-m0011-3p | 0 | 0 | 0 | 0 | 0.198762508 | 0 | 0.295071427 | 0 | 0 | 0 |
| Eca-m0136-5p | 2.70173885 | 1.904376997 | 2.44858722 | 2.363464672 | 2.04219476 | 0.997898446 | 1.059367078 | 2.439795329 | 1.2555823 | 2.05394627 |
| Eca-m0709-3p | 2.518452438 | 1.450386066 | 1.023676043 | 1.768455505 | 1.895760143 | 1.382188497 | 1.72547949 | 2.273436682 | 1.281990099 | 1.620088205 |
| Eca-m0732-3p | 4.50980957 | 2.05252717 | 3.51515908 | 1.433890747 | 2.489732748 | 2.517895836 | 1.943066223 | 2.803120638 | 1.013358192 | 2.916415715 |
| Eca-m0660-5p | 9.544838964 | 9.753029961 | 9.593195565 | 9.834006361 | 9.423773341 | 9.568258813 | 9.967403952 | 9.829600314 | 9.521959172 | 9.694191435 |
| Eca-m0066-3p | 3.677460755 | 3.177722699 | 3.321669846 | 2.714535698 | 3.326320284 | 2.820596671 | 3.342636549 | 3.354811863 | 3.412861053 | 3.103944519 |
| Eca-m0760-5p | 0 | 0.750633086 | 0 | 0 | 0 | 0 | 0 | 0 | 0.06781033 | 0 |
| Eca-m0602-3p | 2.477258448 | 1.549143628 | 2.115652555 | 1.952574339 | 2.286763236 | 2.299861942 | 1.711570195 | 2.010218084 | 1.462723431 | 1.949598073 |
| Eca-m0758-3p | 0 | 0 | 0 | 0 | 0 | 0 | 0 | 0 | 0.297947651 | 0 |
| Eca-m0523-3p | 0 | 0 | 0 | 0 | 0 | 0 | 0 | 0.005895279 | 0 | 0 |
| Eca-m0794-5p | 3.182075714 | 2.625693682 | 3.521314433 | 2.78005186 | 3.795144389 | 3.031472761 | 2.471770292 | 3.494376025 | 2.441091143 | 2.966065091 |
| Eca-m0339-5p | 4.596937443 | 2.91480556 | 4.198311199 | 3.661059322 | 2.746947977 | 2.667670544 | 3.250840115 | 3.186581591 | 2.812699022 | 3.091438254 |
| Eca-m0107-5p | 0.181994566 | 0 | 0 | 0.287889975 | 0 | 0 | 0 | 0.330217916 | 0 | 0 |
| Eca-m0013-3p | 0 | 0 | 0.705757361 | 0.307128554 | 0 | 0.128936472 | 0 | 0 | 0 | 0 |
| Eca-m0309-3p | 2.208642026 | 2.112850319 | 2.303913742 | 2.37462857 | 1.308639204 | 2.055833027 | 1.348451282 | 2.442543353 | 2.730127898 | 2.815450485 |
| Eca-m0293-3p | 1.866334886 | 0.930560872 | 2.832627467 | 2.473676544 | 2.266959304 | 2.294509546 | 1.965229363 | 3.244643755 | 1.468825291 | 1.654473272 |
| Eca-m0143-5p | 4.18773855 | 2.890399432 | 3.658041009 | 4.119047913 | 3.892600643 | 3.402165869 | 3.530325527 | 3.606745002 | 2.581861299 | 3.512774909 |
| Eca-m0245-3p | 4.495431881 | 3.522167999 | 3.864930865 | 4.158781696 | 3.551633899 | 4.340210267 | 3.884523317 | 5.067468644 | 3.532866213 | 3.841197945 |
| Eca-m0551-5p | 0 | 0 | 0 | 1.152702627 | 0 | 0.785104692 | 0 | 0.733829451 | 0 | 0.356884005 |
| Eca-m0504-5p | 2.207528297 | 0.479011917 | 1.980142551 | 0.368767629 | 0.252863453 | 0.821600816 | 0.286530426 | 1.580291953 | 0.566765401 | 0.255578738 |
| Eca-m0460-5p | 2.100591941 | 1.298641562 | 4.043004849 | 1.200944914 | 2.003211106 | 2.417974559 | 2.574233998 | 2.362329284 | 0.843457826 | 2.299089488 |
| Eca-m0298-3p | 0.009906203 | 0 | 0 | 0 | 0 | 0 | 0 | 0 | 0 | 0 |
| Eca-m0376-3p | 0 | 0 | 0 | 0 | 0.328815421 | 0 | 0 | 0 | 0 | 0 |
| Eca-m0563-3p | 2.926772079 | 1.503203314 | 3.165467772 | 2.512368501 | 3.018575057 | 2.173288558 | 2.523713076 | 3.105281621 | 2.267999073 | 2.096638936 |
| Eca-m0071-5p | 0 | 0 | 0.120363854 | 0 | 0 | 0.143557943 | 0 | 0 | 0 | 0 |
| Eca-m0366-3p | 0 | 0 | 0.752673591 | 0 | 0.603848 | 0.544574507 | 0 | 0 | 0.040897969 | 0 |
| Eca-m0034-3p | 1.494671878 | 0.830745733 | 0.612246328 | 1.408860588 | 0.527302778 | 2.111007055 | 0.556628117 | 1.466198894 | 0.793006665 | 1.798589412 |
| Eca-m0798-3p | 0.693569015 | 1.340692088 | 1.680694631 | 0.738369534 | 1.352393768 | 1.294352563 | 0.661612736 | 2.02565849 | 2.258665768 | 0.633705322 |
| Eca-m0607-5p | 0 | 0 | 0 | 0.452949568 | 0.330543265 | 0.276876874 | 0 | 0.505775868 | 0 | 0 |
| Eca-m0515-5p | 0 | 0 | 0.053460832 | 0 | 0 | 0 | 0.409030734 | 0 | 0 | 0 |
| Eca-m0816-3p | 0.198851086 | 0.056759508 | 1.005451391 | 0.236807272 | 1.569979098 | 0.132550523 | 0.171531917 | 1.612272715 | 0.443665818 | 1.24427317 |
| Eca-m0459-3p | 0 | 0 | 1.380325655 | 0 | 0 | 0 | 0.133542486 | 0 | 0 | 0 |
| Eca-m0329-5p | 6.174199172 | 6.600527438 | 5.878507268 | 6.119670972 | 6.330699076 | 6.734064549 | 6.751556171 | 5.952316269 | 7.059998543 | 6.119197316 |
| Eca-m0163-3p | 0.076218209 | 0.390485582 | 0.934113851 | 1.831307914 | 0.018058545 | 0.003830083 | 0.046643951 | 1.566159763 | 0 | 0.650598147 |
| Eca-m0090-3p | 0 | 0 | 0.25638406 | 0 | 0 | 0 | 0 | 0 | 0 | 0 |
| Eca-m0288-3p | 0 | 0 | 0.004099326 | 0.022499796 | 0.991495889 | 0.453734188 | 0.593420347 | 1.358574645 | 0 | 0.50776192 |
| Eca-m0629-3p | 2.255394988 | 1.193898895 | 2.367123097 | 1.208627788 | 2.353865168 | 2.368236957 | 1.090894795 | 3.119646325 | 1.429391094 | 3.106318516 |
| Eca-m0352-3p | 3.87412146 | 2.449350497 | 3.523789212 | 3.508345757 | 3.433993273 | 3.273799266 | 3.008866422 | 3.158144235 | 2.611758665 | 3.626151417 |
| Eca-m0556-3p | 0 | 0 | 0 | 0 | 0 | 0 | 0 | 0.14127389 | 0 | 0 |
| Eca-m0286-3p | 0.126322329 | 0 | 0 | 0 | 0 | 0 | 0.041440676 | 0 | 0 | 0.284833141 |
| Eca-m0446-3p | 2.263682138 | 0.451743277 | 2.110122103 | 0.716582298 | 1.176994459 | 2.468419447 | 1.504441405 | 1.602753576 | 0.479631182 | 0.588245466 |
| Eca-m0647-5p | 3.650754422 | 2.927468737 | 3.422508977 | 3.981112359 | 3.912304878 | 3.387461901 | 4.208459922 | 4.548038546 | 3.911414672 | 3.412755187 |
| Eca-m0031-5p | 1.562878307 | 0 | 0 | 0 | 0 | 0 | 0.814668243 | 0.890193847 | 0.775075611 | 0 |
| Eca-m0313-5p | 7.782543028 | 5.090373078 | 6.472550213 | 6.597900988 | 6.919769011 | 6.686549728 | 5.65424532 | 5.721546249 | 3.774910375 | 6.246890366 |
| Eca-m0820-5p | 0.089715962 | 0 | 0 | 0 | 0 | 0.180188277 | 0.006181123 | 0 | 0 | 0 |
| Eca-m0617-5p | 3.605301804 | 4.835694921 | 3.578754213 | 4.265311778 | 4.369354866 | 4.553584034 | 4.327249827 | 4.359852777 | 4.052221362 | 4.646817017 |
| Eca-m0358-3p | 1.058819283 | 0.28503013 | 1.367167423 | 1.191546813 | 1.206722531 | 0.826963855 | 0.381139142 | 1.24619579 | 0.299602746 | 0.879752803 |
| Eca-m0650-5p | 0.656982118 | 1.371098363 | 1.349072024 | 0.692657622 | 1.265046102 | 0.597393689 | 1.181254355 | 0.707510808 | 1.241952993 | 1.273680569 |
| Eca-m0144-3p | 0.33135438 | 0 | 0 | 0 | 0.127126274 | 0 | 0 | 0 | 0 | 0 |
| Eca-m0387-3p | 3.906195068 | 1.913041302 | 4.001140409 | 3.05453775 | 3.331319629 | 1.966733584 | 2.14625113 | 3.140563512 | 1.944914908 | 3.139552424 |
| Eca-m0548-5p | 2.63524828 | 1.429410061 | 2.449395654 | 2.329188435 | 1.171428345 | 2.946543803 | 1.819560411 | 3.06859576 | 1.65445685 | 2.545356503 |
| Eca-m0114-5p | 0 | 0 | 0 | 0 | 0 | 0 | 0 | 0.0201064 | 0 | 0 |
| Eca-m0370-5p | 1.788952565 | 1.330836461 | 0.981300175 | 1.714425026 | 1.840836396 | 2.301922876 | 1.479976413 | 1.770620661 | 1.243019698 | 1.721292473 |
| Eca-m0369-5p | 2.439633897 | 0.951276727 | 1.83237353 | 0.898543372 | 1.913741748 | 1.722409101 | 0.814220082 | 1.970710185 | 1.029396044 | 1.667894294 |
| Eca-m0028-5p | 0 | 0 | 0 | 0 | 0.134923563 | 0 | 0 | 0 | 0 | 0.015485209 |
| Eca-m0267-3p | 0.006241107 | 0.623339155 | 1.192413881 | 0.037908144 | 0.686142242 | 0.455281614 | 0 | 0.870029595 | 0 | 0 |
| Eca-m0069-5p | 0 | 0 | 0 | 0.918590854 | 0 | 0.282244075 | 0 | 0 | 0 | 0.345509211 |
| Eca-m0589-3p | 2.158151911 | 0.295755637 | 3.233766175 | 0.62299344 | 1.691356976 | 1.611184394 | 0.512990009 | 0.648116805 | 0.332865625 | 1.704166479 |
| Eca-m0200-3p | 3.277801201 | 1.587307962 | 2.313513093 | 2.143186617 | 2.257264397 | 2.502128305 | 1.278125474 | 3.126684343 | 1.813863728 | 2.776118015 |
| Eca-m0236-3p | 0.522659418 | 1.311951802 | 0.545999948 | 0.563407965 | 0.466077556 | 1.63880413 | 1.756527115 | 0.580135564 | 1.254967492 | 1.999901507 |
| Eca-m0112-3p | 0 | 0 | 0.375727468 | 0 | 0.310221656 | 0 | 0.222755082 | 0 | 0 | 0 |
| Eca-m0089-5p | 0.05534841 | 0 | 0 | 0 | 0.220239648 | 0 | 0 | 0.234338734 | 0 | 0 |
| Eca-m0468-3p | 0 | 0 | 0 | 0 | 0 | 0 | 0 | 0.01513462 | 0 | 0 |
| Eca-m0349-3p | 0.181655075 | 0 | 0.032902907 | 0 | 0 | 0 | 0 | 0 | 0 | 0 |
| Eca-m0706-3p | 0.287943067 | 0 | 0.580666582 | 0 | 0 | 0 | 0 | 0 | 0 | 0 |
| Eca-m0665-5p | 0.028200524 | 0.19557259 | 0.767466826 | 0.822600735 | 0.51140512 | 0 | 0.009397058 | 1.123750995 | 0 | 0.518580478 |
| Eca-m0660-3p | 6.553040685 | 6.716228676 | 6.495846394 | 6.839541308 | 6.459856284 | 6.59872162 | 6.338430107 | 5.934634014 | 6.60271469 | 6.599625851 |
| Eca-m0082-3p | 2.358457697 | 1.557974477 | 1.750582436 | 2.251921099 | 2.50618498 | 1.739087936 | 0.979745397 | 1.092293741 | 1.309620805 | 2.070702684 |
| Eca-m0410-5p | 2.242771629 | 1.737104211 | 2.161684585 | 2.231851838 | 2.332564201 | 1.60606416 | 2.111083325 | 2.666304874 | 1.793055368 | 1.663251178 |
| Eca-m0386-3p | 2.033264222 | 1.732992232 | 2.133914849 | 1.093803759 | 1.910361624 | 1.721118734 | 1.55760158 | 1.112610335 | 2.096551129 | 0.989461896 |
| Eca-m0606-3p | 1.07604581 | 0.277209187 | 0.427525857 | 1.457828207 | 1.361777606 | 1.014804984 | 0.381158626 | 1.26664399 | 0.293140102 | 1.372829181 |
| Eca-m0641-5p | 2.235135252 | 1.496685734 | 2.347763654 | 1.087317933 | 2.17662983 | 1.995044665 | 0.996537238 | 2.338351982 | 0.864377551 | 1.82816939 |
| Eca-m0517-3p | 3.659929788 | 2.380064405 | 3.337126858 | 3.000847888 | 2.883828728 | 3.382217084 | 2.666453801 | 3.521014387 | 2.607626781 | 3.437222436 |
| Eca-m0251-5p | 0 | 0 | 0 | 0 | 0.301440936 | 0 | 0 | 0 | 0 | 0 |
| Eca-m0718-5p | 0.95703143 | 1.045016004 | 1.711328177 | 1.01142017 | 0.881080425 | 2.461521203 | 1.901693146 | 2.623467792 | 2.055897229 | 0.884041607 |
| Eca-m0394-3p | 1.143363131 | 0 | 0 | 0 | 0.57338084 | 0.917118123 | 0 | 0 | 0 | 0.391187678 |
| Eca-m0169-5p | 0.229776311 | 0 | 0.317198932 | 0 | 0 | 0 | 0 | 0 | 0 | 0 |
| Eca-m0742-5p | 3.046418575 | 1.672965444 | 2.834847885 | 1.386410778 | 2.50203254 | 2.764239863 | 1.258702455 | 3.569194966 | 2.07907646 | 2.134971993 |
| Eca-m0721-5p | 1.318155302 | 0.445636347 | 1.232533777 | 0.328281011 | 0.75431413 | 0.710693814 | 1.036534621 | 0.34045492 | 0.211974836 | 0.761374913 |
| Eca-m0491-3p | 0 | 0 | 0 | 0 | 0 | 0.294060916 | 0 | 0 | 0 | 0.239353101 |
| Eca-m0170-3p | 0 | 0 | 0.450656907 | 0 | 0 | 0 | 0.422973533 | 0 | 0 | 0 |
| Eca-m0678-3p | 0 | 0 | 0 | 0 | 0 | 0.071427496 | 0 | 0.97360653 | 0 | 0.319153844 |
| Eca-m0583-3p | 0.847810565 | 0.050737515 | 0.924464275 | 0.196366397 | 0.120555107 | 1.288791187 | 0.141855679 | 1.03644475 | 0.064619877 | 1.151247074 |
| Eca-m0215-5p | 0 | 0.410486292 | 0 | 0 | 0 | 0 | 0.638639748 | 0 | 0.67096612 | 0 |
| Eca-m0564-3p | 0.130537328 | 0 | 0 | 0.258748668 | 0 | 0 | 0 | 0.311555692 | 0 | 0 |
| Eca-m0547-3p | 0.206360147 | 0 | 0 | 0 | 0 | 0.30451652 | 0 | 0.382733432 | 0 | 0 |
| Eca-m0232-5p | 0.026581951 | 0 | 0.097574887 | 0 | 0 | 0 | 0 | 0 | 0 | 0.195983298 |
| Eca-m0341-5p | 3.587708554 | 1.35769258 | 1.09134703 | 1.124192167 | 0.932204734 | 2.548669998 | 1.674862947 | 3.26350186 | 1.494420953 | 3.022339472 |
| Eca-m0203-3p | 0.137031815 | 0 | 0 | 0 | 0 | 0 | 0.240436159 | 0 | 0 | 0 |
| Eca-m0596-3p | 0 | 0 | 0 | 0 | 0 | 0 | 0 | 0.0201064 | 0 | 0 |
| Eca-m0647-3p | 2.857210063 | 2.17251657 | 2.971776172 | 1.810508229 | 3.045569549 | 2.880698877 | 3.366602603 | 3.137351683 | 3.146788153 | 2.300861208 |
| Eca-m0453-3p | 4.781013765 | 4.67632908 | 4.633957975 | 3.226172821 | 4.887373753 | 4.419208985 | 3.814987958 | 3.98847001 | 4.277859238 | 4.391461627 |
| Eca-m0505-3p | 1.547614449 | 0.867653167 | 0.570498986 | 0.584570247 | 1.159544753 | 1.245598006 | 1.075281092 | 0.598221768 | 1.351075133 | 1.315045677 |
| Eca-m0421-5p | 0.201777259 | 0 | 0 | 0 | 0 | 0 | 0.749720087 | 0 | 0 | 0 |
| Eca-m0614-3p | 1.155747047 | 0 | 0.069234126 | 0.082174197 | 0.554486448 | 0.687847321 | 0.030430678 | 1.193119375 | 0 | 0.752608875 |
| Eca-m0487-5p | 1.608007396 | 2.270742588 | 1.904457322 | 1.048941874 | 1.591699427 | 1.905237161 | 1.8598839 | 1.066731772 | 1.561029148 | 1.600525035 |
| Eca-m0006-5p | 2.332410395 | 0.185243501 | 0.398849078 | 1.275938357 | 1.29673936 | 0.286430047 | 0.336731106 | 1.61564026 | 0.209859366 | 1.457089575 |

Note: the numbers mean read counts that were normalized using the DESeq2 R package of each miRNAs.

Table S5 microRNAs of Figure 11

| Group | Number | microRNA name | Group1 | | Group2 | | Group 3 | |
| --- | --- | --- | --- | --- | --- | --- | --- | --- |
|  |  |  | log2Foldchange | padj | log2Foldchange | padj | log2Foldchange | padj |
| 1 2 shared | 3 | eca-miR-1298 | 2.865426061 | 0.017831649 | 2.59743291 | 0.0000319 |  |  |
|  |  | eca-miR-299 | 1.473225069 | 0.030390017 | 1.277709803 | 0.042804203 |  |  |
|  |  | eca-miR-9014 | -1.368418495 | 0.044122196 | -3.293665035 | 0.0000129 |  |  |
| 1 3 shared | 1 | Eca-m0793-3p | 1.943533446 | 0.029191844 |  |  | -2.504099803 | 0.002741723 |
| 2 3 shared | 42 | eca-miR-9084 |  |  | -4.153303375 | 3.75E-22 | -2.839878799 | 0.00000253 |
|  |  | eca-miR-8962 |  |  | -3.362474233 | 4.22E-10 | -2.283101061 | 0.0000125 |
|  |  | eca-miR-9077 |  |  | -3.932405066 | 1.52E-14 | -3.048456693 | 0.0000004 |
|  |  | eca-miR-329b |  |  | 1.530556358 | 0.006682766 | 1.224428108 | 0.008662547 |
|  |  | eca-miR-9024 |  |  | -2.67476056 | 8.36E-09 | -1.950749804 | 0.000000439 |
|  |  | eca-miR-370 |  |  | 1.405228707 | 0.004000216 | 1.08663323 | 0.039312653 |
|  |  | Eca-m0818-3p |  |  | -5.010912928 | 0.034248903 | -4.52674785 | 0.009051783 |
|  |  | eca-miR-8908h |  |  | -1.409374042 | 0.005818757 | -1.582064977 | 0.002765235 |
|  |  | Eca-m0471-3p |  |  | -3.038543969 | 0.000655509 | -2.16779041 | 0.002018008 |
|  |  | eca-miR-545 |  |  | -2.695294645 | 0.000000036 | -2.190725048 | 0.000000743 |
|  |  | eca-miR-9049 |  |  | -3.125090272 | 2.29E-09 | -2.21118126 | 0.0000125 |
|  |  | eca-miR-9176 |  |  | -3.355545912 | 7.05E-12 | -3.591106509 | 3.62E-13 |
|  |  | eca-miR-149 |  |  | -4.265878742 | 0.000023 | -4.381008966 | 1.55E-10 |
|  |  | eca-miR-8908b |  |  | -1.498502273 | 0.000562495 | -1.332994076 | 0.012913521 |
|  |  | eca-miR-8909 |  |  | -2.808349048 | 2.66E-11 | -2.248499267 | 0.000000117 |
|  |  | eca-miR-9149 |  |  | -2.901797046 | 0.004728072 | -2.899866572 | 0.000502805 |
|  |  | eca-miR-9121 |  |  | -3.754460342 | 6.91E-11 | -2.52270913 | 0.0000084 |
|  |  | eca-miR-8971 |  |  | -3.236622127 | 0.00000325 | -1.995241543 | 0.008715179 |
|  |  | eca-miR-8998 |  |  | -2.589161317 | 0.001902471 | -2.113514162 | 0.006914435 |
|  |  | eca-miR-34b-3p |  |  | -1.598834762 | 0.005900944 | -1.910808363 | 0.001516501 |
|  |  | eca-miR-8984 |  |  | -3.123029966 | 8.72E-17 | -1.564187159 | 0.00000105 |
|  |  | eca-miR-8908e |  |  | -1.160467005 | 0.007260424 | -1.555261791 | 0.000936155 |
|  |  | eca-miR-8931 |  |  | -3.693398345 | 2.64E-15 | -1.729379503 | 0.003959705 |
|  |  | eca-miR-136 |  |  | 1.593339417 | 0.014301437 | 1.551356936 | 0.005072392 |
|  |  | eca-miR-99a |  |  | 1.107448715 | 0.048667388 | 1.037983819 | 0.032139747 |
|  |  | eca-miR-449a |  |  | -2.222261426 | 1.99E-08 | -2.231375587 | 6.77E-09 |
|  |  | eca-miR-34b-5p |  |  | -2.910770572 | 3.22E-09 | -2.598913969 | 1.45E-08 |
|  |  | Eca-m0743-5p |  |  | -2.060129342 | 0.000634314 | -2.071853326 | 0.000624954 |
|  |  | eca-miR-493a |  |  | -4.127702582 | 4.22E-10 | -3.103576691 | 0.00000109 |
|  |  | eca-miR-214 |  |  | 1.2653259 | 0.005900944 | 1.065131508 | 0.029597964 |
|  |  | eca-miR-181b |  |  | 1.032499069 | 0.02633231 | 1.111244831 | 0.023921941 |
|  |  | eca-miR-8932 |  |  | -5.252655456 | 0.024814205 | -4.825606934 | 0.004101297 |
|  |  | eca-miR-9054 |  |  | -4.861581698 | 1.1E-10 | -3.721555119 | 0.000000153 |
|  |  | eca-miR-8941 |  |  | -4.022329994 | 3.52E-13 | -2.61747338 | 0.0000444 |
|  |  | eca-miR-9038 |  |  | -2.853207716 | 0.000257673 | -2.004633151 | 0.004101297 |
|  |  | eca-miR-381 |  |  | 1.165885615 | 0.034248903 | 1.514393567 | 0.000637265 |
|  |  | eca-miR-34c |  |  | -2.43195786 | 2.91E-10 | -2.374078851 | 0.000000112 |
|  |  | Eca-m0067-5p |  |  | -2.151905439 | 0.001941105 | -2.184720703 | 0.002018008 |
|  |  | eca-miR-449b |  |  | -2.86677868 | 0.000804322 | -2.823402177 | 0.000012 |
|  |  | eca-miR-9079 |  |  | -3.220445382 | 1.72E-10 | -2.73002899 | 8.18E-11 |
|  |  | eca-miR-8979 |  |  | -4.196174504 | 5.09E-17 | -2.486227853 | 0.0000438 |
|  |  | eca-miR-8957 |  |  | -4.000112111 | 9.77E-14 | -3.012441496 | 0.000000153 |
| 1 unique | 17 | eca-miR-450b-5p | 1.397460159 | 0.009701934 |  |  |  |  |
|  |  | eca-miR-508-3p | 1.004931813 | 0.033868644 |  |  |  |  |
|  |  | eca-miR-138 | -2.317602774 | 0.029287409 |  |  |  |  |
|  |  | Eca-m0195-3p | 6.734475316 | 0.008327176 |  |  |  |  |
|  |  | eca-miR-221 | -1.287015372 | 0.036930047 |  |  |  |  |
|  |  | eca-miR-503 | 1.825244045 | 0.000869245 |  |  |  |  |
|  |  | eca-miR-146a | 1.226994264 | 0.030390017 |  |  |  |  |
|  |  | eca-miR-424 | 1.138286747 | 0.020180593 |  |  |  |  |
|  |  | eca-miR-411 | 1.004451379 | 0.043286606 |  |  |  |  |
|  |  | eca-miR-450a | 1.352022406 | 0.005945735 |  |  |  |  |
|  |  | eca-miR-542-3p | 1.569226538 | 0.005945735 |  |  |  |  |
|  |  | eca-miR-483 | 3.712140408 | 0.003181058 |  |  |  |  |
|  |  | eca-miR-130a | 1.27902417 | 0.049105805 |  |  |  |  |
|  |  | eca-miR-874 | 1.238551141 | 0.049105805 |  |  |  |  |
|  |  | eca-miR-450c | 1.211558701 | 0.007275049 |  |  |  |  |
|  |  | eca-miR-133a | 1.301180492 | 0.045265365 |  |  |  |  |
|  |  | eca-miR-199b-5p | 1.517841617 | 0.00638239 |  |  |  |  |
| 2 unique | 17 | eca-miR-9043 |  |  | -3.417093849 | 0.011446309 |  |  |
|  |  | Eca-m0825-5p |  |  | -1.740418751 | 0.048667388 |  |  |
|  |  | eca-miR-379 |  |  | 1.154127573 | 0.003686469 |  |  |
|  |  | eca-miR-122 |  |  | 2.412069657 | 0.048590507 |  |  |
|  |  | eca-miR-378 |  |  | -1.180368322 | 0.02802928 |  |  |
|  |  | eca-miR-9003 |  |  | -2.991765464 | 0.000131729 |  |  |
|  |  | eca-miR-7 |  |  | -1.287428051 | 0.001902471 |  |  |
|  |  | eca-miR-1388 |  |  | 1.086320513 | 0.018755071 |  |  |
|  |  | Eca-m0329-5p |  |  | 1.544025358 | 0.034248903 |  |  |
|  |  | eca-miR-9062 |  |  | -7.724792246 | 0.001537469 |  |  |
|  |  | eca-miR-802 |  |  | -8.233160064 | 0.000164336 |  |  |
|  |  | eca-miR-345-3p |  |  | 2.049590696 | 0.031148242 |  |  |
|  |  | eca-miR-181a |  |  | 1.027002653 | 0.030681792 |  |  |
|  |  | eca-miR-9036 |  |  | -4.563383831 | 0.048667388 |  |  |
|  |  | eca-miR-148a |  |  | 1.268467386 | 0.029373334 |  |  |
|  |  | eca-miR-8950 |  |  | -4.290877688 | 0.005900944 |  |  |
|  |  | eca-miR-8908i |  |  | -1.239554358 | 0.011446309 |  |  |
| 3 unique | 24 | Eca-m0725-5p |  |  |  |  | -6.823714817 | 0.002268298 |
|  |  | Eca-m0460-5p |  |  |  |  | -4.173920709 | 0.0281815 |
|  |  | Eca-m0272-5p |  |  |  |  | -6.025899848 | 0.016117434 |
|  |  | Eca-m0589-3p |  |  |  |  | -6.598063931 | 0.010025303 |
|  |  | Eca-m0399-3p |  |  |  |  | -2.485304888 | 0.005365202 |
|  |  | Eca-m0687-3p |  |  |  |  | 5.538863582 | 0.044367421 |
|  |  | Eca-m0597-5p |  |  |  |  | -5.696638577 | 0.039312653 |
|  |  | Eca-m0514-5p |  |  |  |  | -5.730499133 | 0.031820327 |
|  |  | eca-miR-329a |  |  |  |  | 1.815931208 | 0.034778503 |
|  |  | Eca-m0499-5p |  |  |  |  | -2.457185289 | 0.002687797 |
|  |  | eca-miR-8908f |  |  |  |  | -2.887103829 | 0.003279203 |
|  |  | eca-miR-8908c |  |  |  |  | -1.866820037 | 0.012913521 |
|  |  | eca-miR-8949 |  |  |  |  | -5.956639385 | 0.041393731 |
|  |  | Eca-m0446-3p |  |  |  |  | -6.350392413 | 0.008902363 |
|  |  | Eca-m0810-3p |  |  |  |  | -2.963211159 | 0.003994556 |
|  |  | Eca-m0313-5p |  |  |  |  | -2.995810117 | 0.000158852 |
|  |  | eca-miR-142-5p |  |  |  |  | 1.584375309 | 0.022515285 |
|  |  | Eca-m0204-3p |  |  |  |  | -2.09265625 | 0.004908451 |
|  |  | eca-miR-95 |  |  |  |  | 1.262012386 | 0.009311251 |
|  |  | eca-miR-8908k |  |  |  |  | -1.34313282 | 0.037497317 |
|  |  | eca-miR-361-3p |  |  |  |  | 1.168827862 | 0.028958553 |
|  |  | Eca-m0230-5p |  |  |  |  | -2.245994099 | 0.002687797 |
|  |  | eca-miR-409-3p |  |  |  |  | 1.102277203 | 0.002851063 |
|  |  | eca-miR-8921 |  |  |  |  | -2.389167126 | 0.023058506 |

Note: Group 1: differentially expressed microRNAs between Guanzhong and Chakouyi horses; Group 2: differentially expressed microRNAs between GU4a and CKY2a compared with GU4b and CKY2b ; Group 3: differentially expressed microRNAs between GU1-3, CKY1, and CKY3 compared with CKY2b and GU4b. The threshold of “|log2-fold change|>1, padj< 0.05” were considered significantly different analyzed by DESeq2 R/Bioconductor package.
