# Supplementary figures and images for: Comparative Transcriptomics Analysis of Testicular miRNA from Cryptorchid and Normal Horses
Source: Animals (Basel). 2020 Feb 21;10(2):338. doi: 10.3390/ani10020338 (PMC7070967; doi:10.3390/ani10020338)

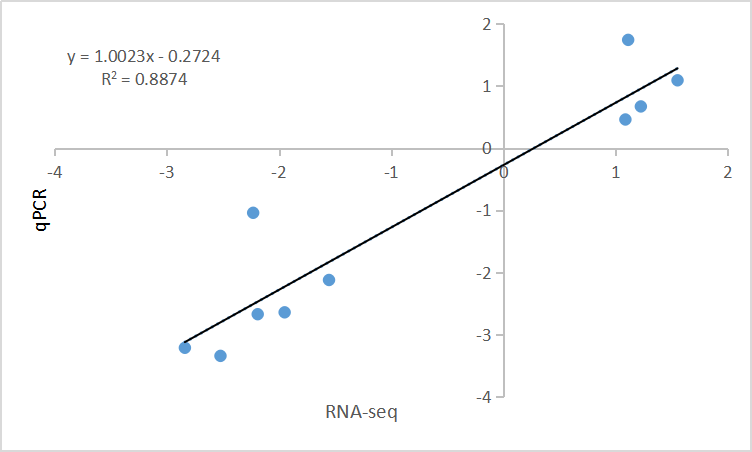

Supplement: Supplementary file 1 [file animals-10-00338-s001.zip › supplement/Figure S1 Correlations of miRNA expression level of 10 differentially expressed miRNA in equine testicular tissues (Group 3)using RNA-Seq and qRT-PCR..tif]
